# Dim artificial light at night alters gene expression rhythms and growth in a key seagrass species (*Posidonia oceanica*)

**DOI:** 10.1038/s41598-023-37261-3

**Published:** 2023-06-30

**Authors:** L. Dalle Carbonare, A. Basile, L. Rindi, F. Bulleri, H. Hamedeh, S. Iacopino, V. Shukla, D. A. Weits, L. Lombardi, A. Sbrana, L. Benedetti-Cecchi, B. Giuntoli, F. Licausi, E. Maggi

**Affiliations:** 1 Institute of Life Sciences, Scuola Superiore Sant’Anna, Piazza Martiri Della Libertà, 56127 Pisa, Italy; 2 Dipartimento di Biologia, Universita’ di Pisa, CoNISMa, Via Luca Ghini 13, 56126 Pisa, Italy; 3 Department of Biology, University of Oxford, Oxford, OX1 3RB UK

**Keywords:** Ecophysiology, Light responses, Plant ecology, Plant evolution, Plant molecular biology, Plant physiology, Plant stress responses

## Abstract

Artificial light at night (ALAN) is a globally spreading anthropogenic stressor, affecting more than 20% of coastal habitats. The alteration of the natural light/darkness cycle is expected to impact the physiology of organisms by acting on the complex circuits termed as circadian rhythms. Our understanding of the impact of ALAN on marine organisms is lagging behind that of terrestrial ones, and effects on marine primary producers are almost unexplored. Here, we investigated the molecular and physiological response of the Mediterranean seagrass, *Posidonia oceanica* (L.) Delile, as model to evaluate the effect of ALAN on seagrass populations established in shallow waters, by taking advantage of a decreasing gradient of dim nocturnal light intensity (from < 0.01 to 4 lx) along the NW Mediterranean coastline. We first monitored the fluctuations of putative circadian-clock genes over a period of 24 h along the ALAN gradient. We then investigated whether key physiological processes, known to be synchronized with day length by the circadian rhythm, were also affected by ALAN. ALAN influenced the light signalling at dusk/night in *P. oceanica*, including that of shorter blue wavelengths, through the *ELF3*–*LUX1*–*ZTL* regulatory network*,* and suggested that the daily perturbation of internal clock orthologs in seagrass might have caused the recruitment of *PoSEND33* and *PoPSBS* genes to mitigate the repercussions of a nocturnal stress on photosynthesis during the day. A long-lasting impairment of gene fluctuations in sites characterised by ALAN could explain the reduced growth of the seagrass leaves when these were transferred into controlled conditions and without lighting during the night. Our results highlight the potential contribution of ALAN to the global loss of seagrass meadows, posing questions about key interactions with a variety of other human-related stressors in urban areas, in order to develop more efficient strategies to globally preserve these coastal foundation species.

## Introduction

Artificial light at night (ALAN) has come to the fore as one of the most impactful anthropogenic factors that affect wildlife^1,2^. With a rate of 6% increase of sky brightness per year, ALAN is spreading globally, affecting over 23% of the world’s land surfaces between 75° N and 60° S^3,4^. In particular, European countries and United States are those most exposed to light-polluted nights, with 88% and almost 50% of the total surface, respectively^4^.

An alteration of the night skyglow by artificial light is expected to impact on the physiology of a wide range of organisms, including animals and plants, as well as the structure of ecological communities^1^. Most life forms on Earth have evolved complex biochemical and molecular circuits that are entrained by light/dark cycles and allow memory and anticipative adaptation to environmental fluctuations^5^. Such regulatory processes display an oscillatory pattern of about 24 h, that is termed as circadian rhythms or the internal clock^5^. In addition to the joint role that light plays for all the organisms to entrain circadian rhythms and as a source of information from the surrounding environment, light radiation in plants is also the main driving force to fix carbon into organic compounds through photosynthesis^6^. While the threat of light pollution and its effect on terrestrial ecosystems are well documented, the implication that ALAN might have in marine habitats has become a relevant issue only over the last decade^7^. Indeed, several of the world’s largest metropolises are located along the coasts, where they contribute to illuminate both the shoreline and the adjacent shallow sea^7^. In this scenario, Europe holds the record of the continent with the highest percentage (54%) of light polluted coasts^8^.

Along the coastline, ALAN is mostly identified as a localized disturbance related to urban areas and originating from fixed light sources, either municipal or private. The spatial arrangement and type of lamps, however, create a quite heterogeneous light environment at the scale of meters, with higher and lower light intensity areas of variable extent and spectrum^9^. As for the light spectrum, recent advances in lighting technologies have boosted the use of broader-spectrum artificial lights (e.g., Light Emitting Diodes/LEDs) in urban areas, due to their improved energy efficiency and enhanced visual performances^10^. Despite their benefits, broader-spectrum lights have created more complex patterns of lighting as compared to natural moonlight, especially along the water column. In fact, the scattered blue component of the spectrum penetrates deeper than the red one, mostly affecting those species that are sensitive to short wavelengths^1,8^. As an additional source of spatial variability, the artificial skyglow extends the ‘sphere of potential influence’ of ALAN far beyond the localization of the point sources^1^, by creating gradients of light intensity tens of kilometres long, while moving away from urban centres along the coastline.

Recent advances in marine ALAN research focused mostly on the effect of direct sources of LED lights on the physiology and behaviour of animals, either in intertidal or shallow subtidal systems (invertebrates and vertebrates; e.g.^11–14^, corals; e.g.^15,16^). To the best of our knowledge, effects on primary producers have been investigated only on microbial species^15,17–19^. This is at odds with the key role played by macroalgae and plants in marine habitats, both as oxygen producers and habitat-forming species (e.g., canopy-forming macroalgae, kelps and seagrasses^20,21^).

The need for studies on ALAN effects on marine plants is further stimulated by research on trees commonly planted along roads (*Populus* ssp., *Platanus* ssp. and *Salix* spp.), which revealed species-specific sensitivity to artificial night lighting^22^. For instance, the proximity to streetlamps can greatly impact plant fitness and phenology in case of *Platanus* spp., leading to the retention of leaves into winter and the anticipation of budburst in spring^23^. Studies on herbaceous plants reported temporally variable, species-specific and light source dependent outcomes, both on growth-related variables and phenological aspects^24–26^. Interestingly, experimental exposure of bean plants to a gradient of artificial night light intensity showed a positive correlation of ALAN with plant biomass, suggesting potential effects of intensities as low as those experienced far from direct light sources^27^.

The seagrass *Posidonia oceanica* (L.) Delile is the most important endemic seagrass along Mediterranean coastlines, covering about 2% of the seafloor^28^. It is a marine monocot species, whose meadows are adapted to grow across a broad depth range, from shallow waters down to 45-m depths, which expose them to different light and temperature regimes^29^. *P. oceanica* supports marine biodiversity through the provision of habitat and food and provides a wealth of services, ranging from the exportation of organic matter, carbon sequestration, sediment trapping and reduction of coastal erosion^20^. Like most seagrasses worldwide, occurrence along coastal areas poses *P. oceanica* meadows under threats related to urban/port development and activities, resulting, in the worst-case scenario, in their regression^30,31^. For those reasons, we chose *P*. *oceanica* (hereafter, *Posidonia*) as model species to study the effects of ALAN on shallow-water seagrass populations next to urban areas, taking advantage of a decreasing gradient of nocturnal light intensity along the coastline south of the city of Leghorn (Italy).

We hypothesized that continuous exposure to artificial night lighting could impact the entrainment of gene expression rhythms in *Posidonia*. Among several physiological processes, circadian rhythms regulate photoprotection and photosynthesis^32^ and induction of flowering^33^. We therefore investigated whether ALAN modified the oscillatory expression of putative circadian genes in *Posidonia*. We further extended our analysis to photosynthetic and photoprotection genes that could participate in the output of the circadian clock, and to the regulators of photoperiodic flowering in the land angiosperm *Arabidopsis thaliana*. To determine if ALAN effect on gene expression could affect performances of *Posidonia* plants, we monitored in situ the maximum photosystem II (PSII) efficiency on dark-adapted leaves along the gradient of nocturnal light intensity (as a reliable measurement of the plant sensitivity to environmental changes^34^). Finally, to assess whether these effects were long-lasting, we monitored plant fitness in terms of growth under controlled conditions and without lighting during the night.

## Material and methods

### Sites location and sampling

**Figure 1:**
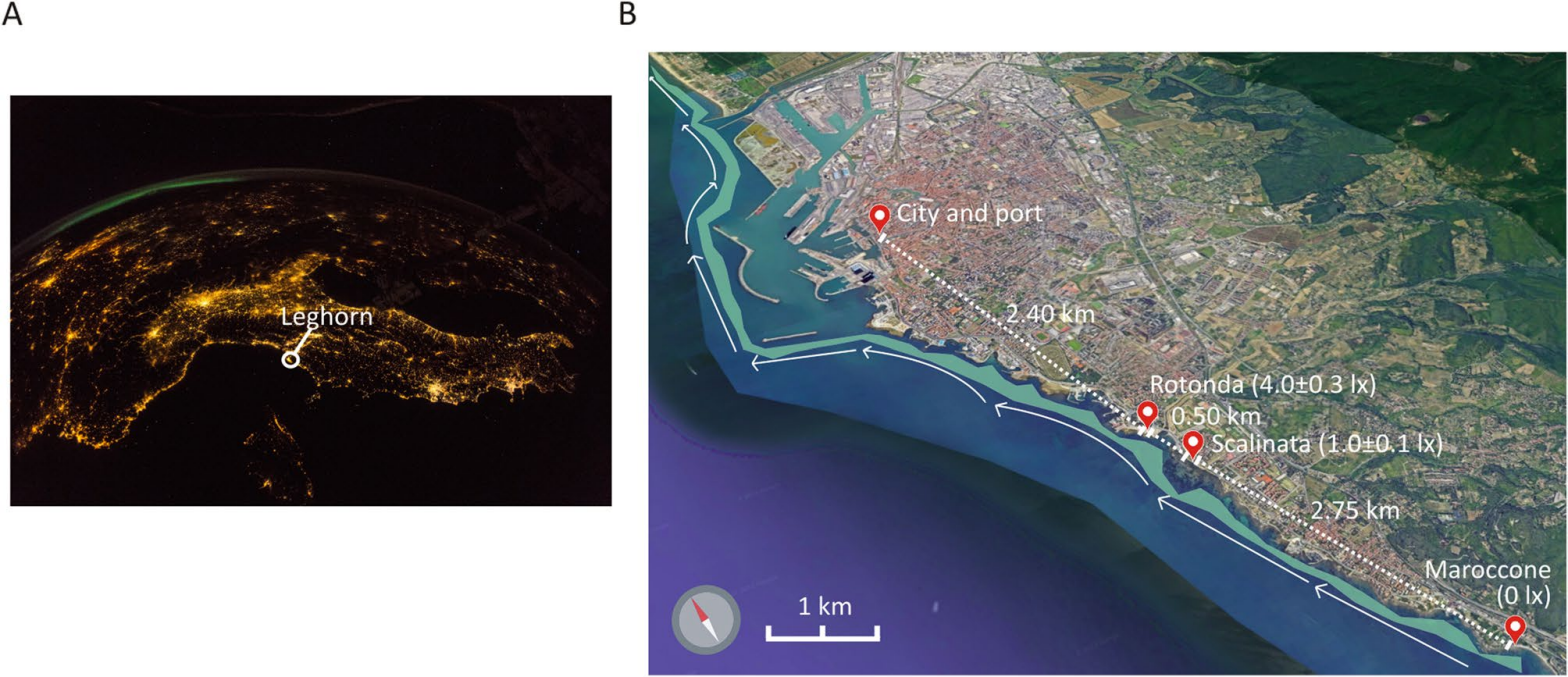
Diffusion of light pollution at night along the Leghorn coastline. (**A**) Map showing the skyglow over Europe as it appeared from space; the city of Leghorn is delimited by a white circle. Photo was taken on 13th November 2018 (credit ESA/NASA; https://www.esa.int/ESA_Multimedia/Images/2018/11/Mapping_the_night). (**B**) Satellite image of a portion of Tuscany (Italy), the distribution of *Posidonia* meadows is shown in green^36^; the circulation of the surface water along the coast is indicated by white arrows^37^. The city and the port of Leghorn are labelled on map together with the three sites where *Posidonia* plants were collected, distances between the city and sites are also reported for comparison (modified from Google Earth, version 9.152.0.1: https://earth.google.com/web/search/43.512270,+10.317117/@43.52372365,10.31830617,6.92320943a,12856.72487566d,35y,28.45564798h,37.3416394t,0r/data=CigiJgokCTrXa2BEwUVAEVRf3M-6wEVAGZUGYPbVpiRAIbgMprlboCRA). The values of illuminance detected for each site are reported in lux in brackets on the map. Images in (**A**) and (**B**) have been modified with the help of ‘CorelDRAW Graphics Suite 2021’ software.

This study was carried out at three locations located south of the city of Leghorn (Italy, Western Mediterranean Sea): Rotonda (43°30′44.2″N 10°19′01.6″E), Scalinata (43°30′28.1″N 10°19′08.1″E) and Maroccone (43°29′03.9″N 10°19′51.9″E). All locations were characterized by a gently sloping rocky seabed, with occasional presence of pebbles and small boulders. Within a distance of 10–20 m from the shoreline, the biological assemblage was dominated by turf-forming and encrusting algae, with patches of *Posidonia*; due to the shallow water depth (about 0.5–1 m), seagrass leaves could reach the surface under low tide conditions. Despite their geographical proximity, the three locations differ in (1) night sky brightness (measured through a Sky Quality Meter, SQM—Unihedron; the unit of measurement is magnitude per squared arcsecond; Maroccone: 19.945 mag/arcsec^2^, Scalinata: 18.4975 mag/arcsec^2^, Rotonda: 15.3675 mag/arcsec^2^), (2) intensity of artificial light at night (defined as illuminance; the unit of measurement is lux; Maroccone: 0 ± 0.1 lx [≈ 0  µmol/s/m^2^], Scalinata: 1 ± 0.1 lx [≈ 0.01 µmol/s/m^2^], Rotonda: 4.0 ± 0.3 lx [≈ 0.1 µmol/s/m^2^]) and (3) distance from the industrial and merchant port (Fig. 1). We measured consistent values (as indicated in (1) and (2) above) of night sky brightness and illuminance during new moon nights in mid-June in 2017 and 2019, supporting the evidence that urban nocturnal light remains constant throughout the years. Night sky brightness and illuminance levels at the three locations decreased when moving from the centre to the periphery of the city. Despite being approximately only 500 m apart from Rotonda, Scalinata was characterized by relatively low nocturnal illuminance (more similar to Maroccone than Rotonda). We also used the LANcube system to collect data on the red and blue bands of the spectrum at the two illuminated sites^35^. Data on LANcube's color blue and red bands ratios to the clear band (which are linked to the percentage of blue and red to the total visible light, respectively) showed no statistically significant differences between the two sites (mean ± 1 SE (standard error); Red/Clear: Scalinata 1.7824 (1.0060), Rotonda 2.5103 (0.0036). Blue/Clear: Scalinata 0.3810 (0.2152), Rotonda 1.2067 (0.0071) (tested through one-way ANOVA).

A total of 70 *Posidonia* samples were collected at 4-h intervals over a period of 24 h at 19:00, 23:00, 3:00, 7:00, 11:00, 15:00 and 19:00 (GTM + 1), at 1-m depth, in mid-June 2017 (number of replicates at each time point: n = 10), characterised by an average of 15 h of light and 9 h of darkness (dawn and dusk approximately at 5:00 and 21:00 GTM + 1 according to https://www.timeanddate.com/). Each replicate consisted of one tip of 2 cm-length selected from the youngest, fully mature leaves collected from the same seagrass meadow, avoiding those covered by epiphytes, and subsequently stored in RNA*later* tissue collection (Sigma-Aldrich). The study on plant complies with relevant institutional, national, and international guidelines and legislation. The collection of *P. oceanica* material was performed in line with the guidelines stated in the permit granted by the city of Leghorn to the University of Pisa in order to collect plant material for research purpose (*Determinazione N.3030 del 29/05/2020*).

### Phylogenetic analysis and choice of putative circadian genes

To the best of our knowledge, putative circadian clock genes and the daily fluctuation of the respective mRNAs have been only recently studied in two seagrass species, *Cymodocea nodosa* and *Zostera marina*^38^, but never in *P. oceanica*. Thus, we conducted a detailed phylogenetic analysis to retrieve the closest homologs of *Arabidopsis thaliana* circadian genes in *Posidonia* and compare their relatedness among various angiosperms, both monocotyledons and eudicotyledons, which thrive in terrestrial or aquatic environments. We focused on genes whose oscillatory expression leads to the establishment of the main endogenous circadian rhythm in *A. thaliana*: *CIRCADIAN CLOCK ASSOCIATED 1* (*CCA1*), *REVEILLE* (*RVE8*), *PSEUDO-RESPONSE REGULATOR 5* (the two orthologues *PRR5-1* and *PRR5-2*) and *GIGANTEA* (*GI*), which constitute the morning loop, and *LUX ARRHYTHMO* (*LUX1*), *EARLY FLOWERING 3* (*ELF3*) and *ZEITLUPE* (*ZTL*), which, instead, participate to the evening loop^39^. We did not find evidence of a *TOC1* gene in *Posidonia* as the closest homologous of the *Arabidopsis TOC1* was more similar to the *PRR5* gene family. We also performed a similar phylogenetic analysis on the flowering regulator *CONSTANS* (*CO*), whose expression is tightly controlled by the endogenous clock in terrestrial angiosperms^33^.

We selected *Posidonia* and *Zostera marina* as examples of aquatic monocots, *Nelumbo nucifera*, the so-called ‘sacred lotus’, for the aquatic eudicots, *Arabidopsis thaliana* as terrestrial eudicot and *Oryza sativa* for the group of terrestrial monocots. Because of their key phylogenetic position in the evolution of plants on land, we also included the lycophyte *Selaginella moellendorffii* and the liverwort *Marchantia polymorpha*, whose homologs of the core clock genes have been recently identified^40^. Protein sequences were retrieved applying the BLAST algorithm^41^ to search the Phytozome (http://www.phytozome.net), DDBJ/ENA/GenBank (accession GEMD01000000, BioProject ID: PRJNA315106) and NCBI (https://www.ncbi.nlm.nih.gov) databases (Table S1) and *Arabidopsis* protein sequences were used as queries. The resulting sequences were then aligned back against the *Arabidopsis thaliana* protein database to ensure that they represent its closest homologs (Supplementary Files S1–S8). CCA1, PRR5, GI, ZTL, ELF3, LUX, RVE protein sequences for *Marchantia polymorpha*, *Selaginella moellendorffii*, *Oryza sativa* and *Arabidopsis thaliana* were based on^40^ (Table S1). The phylogenetic analysis was performed using MEGAX^42^. The phylogenetic trees were generated firstly by aligning the retrieved sequences using the MUSCLE algorithm^43^ and secondly by applying the Maximum Likelihood method and a JTT matrix-based model^44^. The bootstrap analysis (500 repeats) returned the percentage of trees in which the associated taxa clustered together.

### Choice of genes involved in light-dependent processes in *Posidonia*

Given the capacity of a continuous source of light at night to modify the oscillatory expression of circadian genes in *Posidonia*, we then selected five representative genes (*Chlorophyll a/b binding protein 4*, *PoLHCA4*; *Chlorophyll a/b binding protein CP29.2*, *PoLHCB4.2*; *Chlorophyll a/b binding protein 6A*, *151*, *PoCAB-6A*, *PoCAB-151*; *Photosystem I reaction centre subunit V*, *PSAG*), from the original group already analysed in^45^, and which are known to be involved in different aspects of the photosynthetic process. To test the hypothesis that excessive night lighting could cause photo-oxidative damage and, consequently, activate photoprotective mechanisms in *Posidonia* tip leaves, we analysed the expression of two photo-protection marker genes, *Zeaxanthin epoxidase* (*PoZEP*) and *Photosystem II 22 kDa protein* (*PoPSBS*), and of one photosynthesis-related gene, *Ferredoxin-1* (*PoSEND33*), during the usual time course. Finally, we analysed the expression of *Ribulose-bisphosphate carboxylase small chain 5B*, *PoSSU5B* (encoding for Ribulose-1,5-Bisphosphate Carboxylase/Oxygenase, RuBisCO, small subunit) and the expression of *Alcohol dehydrogenase* (*PoADH*), considered as a marker for various stresses in plants.

### RNA extraction and cDNA synthesis

Total RNA was extracted from *Posidonia* leaf tips, 2 cm long, according to the procedure described in^46^. The addition of fifty milligrams of polyvinylpyrrolidone (PVP) to each sample during the grinding step was the only modification introduced to the original protocol. Half microgram of RNA was subjected to DNase treatment along with cDNA synthesis, both performed with Maxima First Strand cDNA Synthesis Kit (Thermo Scientific) for subsequent quantitative real-time PCR amplification (RT-qPCR).

### Design of primers and quantifications of gene expression via RT-qPCR

RT-qPCR primers from^47^ were used for *PoPSBS*, *PoZEP*, *PoSSU5B, PoLHCA4*, *PoPSAG* genes; primers from^48^ were used for *PoCAB-6A*, *PoCAB-151*, *PoLHCB4.2* and *PoSEND33*. RT-qPCR primers for *PoPRR5-1*, *PoPRR5-2*, *PoELF3*, *PoLUX*, *PoREV1*, *PoCCA1*, *PoZTL*, *PoGI*, *PoCO* and *PoADH* were designed on *Posidonia* transcriptome available at DDBJ/ENA/GenBank (accession GEMD01000000, BioProject ID: PRJNA315106) and their sequences are listed in Table S2. RT-qPCR was carried out with the CFX384 Touch sequence detection system (Bio-Rad), using a SYBR Green PCR Master Mix (Thermo Scientific). 12.5 ng cDNA template were used in each 10 µl amplification reaction. *Ubiquitin* (GeneBank: GO347619) and *60S ribosomal protein L23* (GeneBank: GO347779), *Posidonia* housekeeping genes described in^49^, were chosen as reference genes in our experimental set up as their raw Ct values remained constant during the time course sampling (Fig. S1). The average Ct values of *Ubiquitin* and *L23* was used as reference value to calculate gene expression of target genes. Relative quantification of the expression of each gene was performed using the comparative threshold cycle (Ct) method^50^. Data were reported as log_2_ x^−FC^, where *x* is a value based on primers efficiency *E* (x = 1 + *E*/100) and *FC* stands for expression fold change of the tested gene, calculated as difference in ΔCt from the reference (average ΔCt for samples collected in Maroccone). Primers percent efficiencies (*E*) are listed in Table S2 and *x*^*−FC*^ values are listed in Table S3. Two technical replicates were used for each of the ten biological samples.

### Maximum photosystem II efficiency

Maximum efficiency of photosystem II (PSII) of plants from Maroccone, Scalinata and Rotonda was evaluated in the field by measuring chlorophyll *a* fluorescence with a submersible fluorimeter (Diving PAM, Walz), on fully mature leaves, avoiding those covered by epiphytes, at 12:00, in mid-June 2019. Maximum quantum efficiency of PSII was determined as F_v_/F_m_, where F_v_ indicates the variable fluorescence, calculated as the difference between the maximum fluorescence after the saturation of the light pulse (F_m_) and the minimum fluorescence (F_0_) after 5 min of dark adaptation (Table S4). Measurements of ALAN and illuminance were repeated and confirmed to be consistent with those collected in 2017 (as reported above in the description of the sites location). Five biological replicates were used in the experimental setup.

### Growth analysis

*Posidonia* plants were collected from the three sites of Rotonda, Scalinata and Maroccone, in mid-June 2019, transferred in glass tanks (aquaria) where they were acclimated for 2 weeks and sequentially grown under the same and controlled environmental conditions (22 °C/18 °C day/night with a 16 h photoperiod and 80 µmol photon m^−2^ s^−1^ light intensity, no illumination during the night cycle). The aquaria were filled with water collected in Maroccone at the same time when plants were also harvested. Growth rate was measured on youngest fully mature leaves, newly emerged in the aquaria system, using a modified Zieman's method^51^. Each plant was marked by punching all leaves together with a hypodermic needle, just above the ligula of the most external leaf. Growth was monitored over 14 days, by tracking the distance of the puncture mark from the ligula. Five biological replicates (shoots) were considered for Maroccone and six for Scalinata and Rotonda plants.

### Statistical analysis

Generalized additive mixed effect models (GAMMs) were used to evaluate the effect of light intensity on time series of residuals of gene expression derived from the regression-adjusted analysis. GAMMs allow flexibly in inferring the relationship between a response variable and a number of predictors and to deal with auto-correlated observations and complex variance–covariance structures^52^. For each site, gene expression was modelled as a cubic regression spline in the fixed part of the model. To account for the relationship between gene-expression and time among locations (light intensity), Site and Time (hours) were also included in the random part as random intercept and slope, respectively. GAMMs were fitted using the gamm function of the package *gmcv*, in R 3.5.1. Model assumptions were assessed visually using plots of residuals vs. fitted values, box-plots of residuals vs. experimental conditions (Site and Time) and QQ-plots of standardized residuals vs. normal quantiles^53^. Because study sites may be characterized by additional confounding environmental factors aside from ALAN, we performed GAMM on residuals of gene expression. For this purpose, long-term means (2000–2014) of daily maxima of Surface Sea Temperature (SST), Chlorophyll *a* concentration, Photosynthetic Active Radiation (PAR), diffusion attenuation coefficient, nitrate concentrations, phosphorous concentrations, dissolved O_2_ and iron concentration were retrieved for each study site from Bio-ORACLE database^54^. We finally regressed the gene expression against the linear combination of all environmental variables and extracted the residuals that were used as response variables in the analysis with GAMM. A linear model was used to estimate differences in maximum photosynthetic efficiency and growth rate between sites characterized by different night light intensities. This analysis was performed using the lm function of the package *stats*, in R 3.5.1.

## Results

### Phylogeny of *Posidonia* putative circadian clock genes

**Figure 2:**
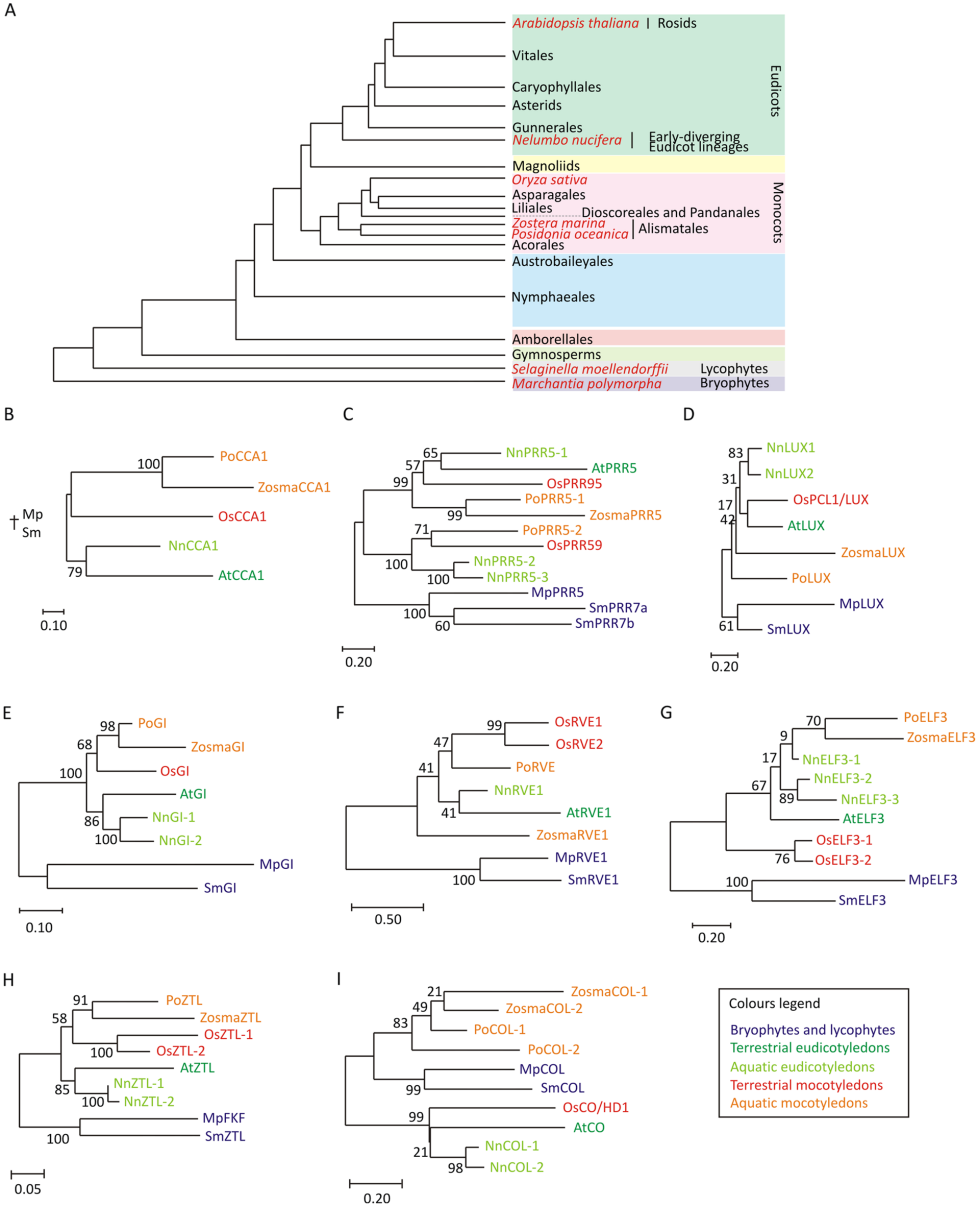
Phylogenetic analysis of circadian clock proteins and PoCO (CONSTANS) in *Posidonia*. (**A**) Phylogeny of land plants. Simplified phylogenetic tree adapted from^57^, showing the evolutionary relationship among plant lineages. Species selected for further phylogenetic analysis, which are presented in this study, are in red. (**B**–**I**) Unrooted phylogenetic trees of the main proteins regulating the morning and evening phases of the circadian rhythm (PoCCA1, PoPRR5-1, PoPRR5-2, PoLUX, PoGI, PoRVE, PoELF3 and PoZTL) and CONSTANS (PoCO) considered in this study, in aquatic monocots (*Posidonia oceanica* and *Zostera marina*) and an eudicot (*Nelumbo nucifera*), terrestrial eudicot (*Arabidopsis thaliana*) and monocot (*Oryza sativa*), lycophytes (*Selaginella moellendorffii*) and liverwort (*Marchantia polymorpha*). Phylogenetic relatedness was inferred by using the Maximum Likelihood method and JTT matrix-based model. Results of the bootstrap analysis are shown next to each branch.

Putative circadian clock proteins as LUX, RVE and ELF3 showed two main clusters that matched the distinction between angiosperms and non-angiosperms species (Fig. 2A,D,F,G). On the other hand, CCA1, GI and ZTL showed an additional sub-cluster within the group of the angiosperms, separating monocotyledon from eudicotyledon species (Fig. 2A,B,E,H). A special case was instead represented by PRR5 proteins, where the sub-cluster containing the closest *Arabidopsis* homolog showed a separation of *Posidonia* and *Z. marina* PRR5s from those of rice, lotus and *Arabidopsis* (Fig. 2C). For both *Posidonia* and *Z. marina*, whose flowering cycles are unpredictable and influenced by various environmental conditions^55,56^, we observed that their best CO homologous hits clustered separately from those of rice, *Arabidopsis* and lotus (Fig. 2A,I). Moreover, they subclustered with CO-like proteins found in *Marchantia* and *Selaginella* which do not produce flower-like structures.

### Artificial light at night impacts the expression of putative circadian clock genes in *Posidonia*

Field measurements presented in this study were performed in mid-June 2017, when in the West Mediterranean Sea days are typically characterized by an average of 15 h of light and 9 h of darkness, with dawn and dusk occurring approximately at 5:00 and 21:00 GTM + 1.

**Figure 3:**
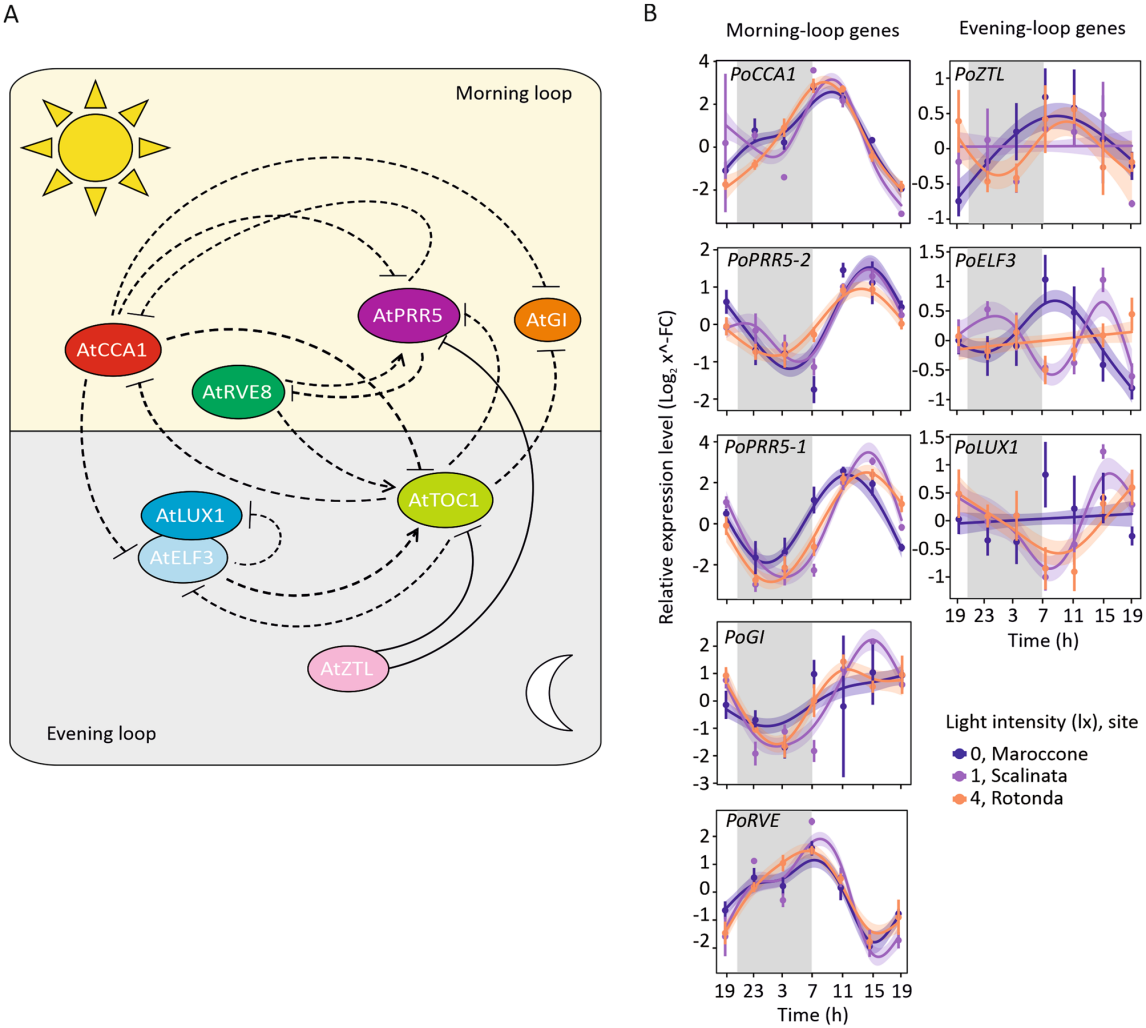
*Posidonia* circadian genes are influenced by lit nights. (**A**) Simplified representation of the relationships between the core genes of the circadian clock in the land angiosperm *Arabidopsis thaliana*. Dashed lines and solid lines indicate transcriptional or post-transcriptional regulation, respectively. The scheme is original, inspired by the text in^58,59^ and created it with the help of ‘CorelDRAW Graphics Suite 2021’ software. (**B**) Relative expression levels (Log_2_ x^−FC^) of five morning-loop genes (*PoCCA1*, *PoRVE*, *PoPRR5-1*, *PoPRR5-2* and *PoGI*), on the left, and three evening-loop genes (*PoZTL*, *PoELF3* and *PoLUX1*), on the right, measured in *Posidonia* leaf tips collected from Maroccone (dark blue), Scalinata (purple) and Rotonda (orange). Samples were collected every 4 h, starting from seven o’clock in the evening for a total period of 24 h. Data are presented as mean ± SE (number of replicates: *n* = 10) and analysed by Generalized Additive Mixed Models (GAMM) for significance.

Among the eight putative circadian clock genes evaluated (Fig. 3A), all but *PoLUX1* showed a significant oscillatory pattern of expression across 24 h under naturally dark conditions at night (S (Time): Maroccone, Fig. 3B, Table 1). Unexpectedly, *PoLUX1* showed a rhythmic expression in its transcriptional level only under dim and intense night light conditions (i.e., at Scalinata and Rotonda sites; Table 1), whereas *PoGI* expression pattern, although significantly described by a cubic spline in Maroccone, showed a different oscillatory behaviour compared to the other two sites (Table 1). The *Posidonia* homolog of *Arabidopsis ZTL* exhibited a peak of expression at the end of the night, which resembled the pattern of oscillation observed in *Z. marina* but not in *C. nodosa*^38^.

**Table 1:** Results of Generalized Additive Mixed Models (GAMMs) on gene expression levels. ^^^0.05 < p < 0.1, *p < 0.05, **p < 0.01, ***p < 0.001.

	*PoCCA1*	*PoRVE1*	*PoPRR5-2*	*PoGI*	*PoLUX1*	*PoELF3*	*PoPRR5-1*	*PoZTL*
Parametric coefficient								
Intercept	0.06 (0.12)	− 0.04 (0.11)	0.06 (0.09)	− 0.04 (0.15)	0.04 (0.11)	0.03 (0.08)	0.13 (0.15)	0.05 (0.10)
Lux	− 0.03 (0.05)	− 0.01 (0.05)	− 0.02 (0.04)	0.03 (0.06)	− 0.01 (0.04)	− 0.01 (0.03)	− 0.08 (0.06)	− 0.01 (0.04)
Approximate significance of smooth terms								
	edf	edf	edf	edf	edf	edf	edf	edf
S (Time): Maroccone	4.293***	4.478***	4.225***	2.743**	1.000	3.358***	4.244***	2.252**
S (Time): Scalinata	4.586***	4.706***	4.294***	4.229***	3.975**	4.382*	4.500***	1.000
S (Time): Rotonda	4.354***	4.280***	3.843**	4.079***	2.682*	1.000	4.278***	3.154
R^2^ adj	0.697	0.564	0.468	0.488	0.153	0.191	0.639	0.106

	*PoLHCA4*	*PoLHCB 4.2*	*PoCAB-6A*	*PoCAB-151*	*PoPSBS*	*PoSEND33*	*PoZEP*	*PoPSAG*
Parametric coefficient								
Intercept	0.003 (0.06)	− 0.02 (0.09)	0.01 (0.08)	0.003 (0.09)	− 0.03 (0.07)	− 0.04 (0.08)	− 0.004 (0.05)	− 0.01 (0.08)
Lux	0.001 (0.03)	0.005 (0.04)	− 0.01 (0.03)	− 0.004 (0.04)	− 0.01 (0.03)	0.02 (0.03)	0.003 (0.02)	0.01 (0.03)
Approximate significance of smooth terms								
	edf	edf	edf	edf	edf	edf	edf	edf
S (Time): Maroccone	3.940***	1.000^^^	3.719**	2.448**	1.000^^^	1.000^^^	1.000*	2.622***
S (Time): Scalinata	4.352***	3.687***	3.783***	2.844*	4.215***	4.522***	3.531***	4.110***
S (Time): Rotonda	4.427***	1.993^^^	1.000	1.000	4.484***	4.559***	1.516	3.558***
R^2^ adj	0.538	0.237	0.22	0.133	0.526	0.522	0.154	0.443

	*PoCO*	*PoSSU5B*	*PoADH*					
Parametric coefficient								
Intercept	0.05 (0.18)	0.04 (0.08)	0.11 (0.13)					
Lux	− 0.02 (0.08)	− 0.03 (0.03)	− 0.05 (0.05)					
Approximate significance of smooth terms								
	edf	edf	edf					
S (Time): Maroccone	2.093^^^	2.441**	3.342***					
S (Time): Scalinata	3.026*	3.155***	2.848***					
S (Time): Rotonda	1.000	2.965**	1.000**					
R^2^ adj	0.082	0.299	0.34					

Among the putative evening-loop genes, the transcriptional levels of *PoELF3* in plants exposed to dim night lighting (i.e., at Scalinata) appeared comparable in comparison to dark conditions (i.e., at Maroccone), while the rhythmic expression completely disappeared with intense ALAN (i.e., at Rotonda). A loss of rhythmic expression in presence of dim night light in Scalinata was observed in case of *PoZTL* (Fig. 3B).

### Lit night effects on gene expression of light-dependent processes in *Posidonia*

**Figure 4:**
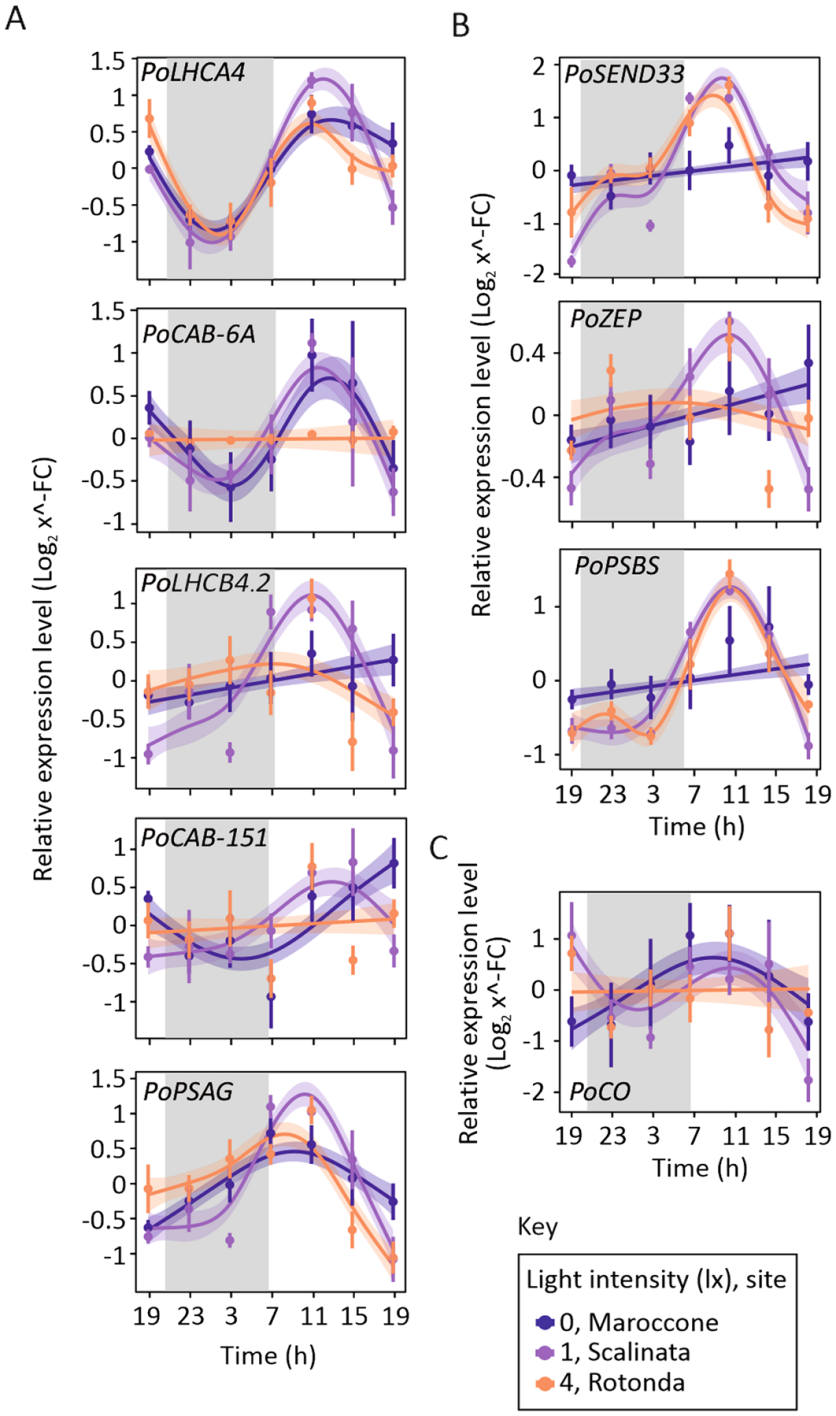
Light-dependent genes in relationship with artificial light at night in *Posidonia*. Relative expression level (Log_2_ x^−FC^) of genes of the light-harvesting complex (*PoLHCA4*, *PoLHCB4.2*, *PoCAB-6A*, *PoPSAG* and *PoCAB-151*) (**A**), photosynthesis and photo-protection (*PoSEND33, PoPSBS* and *PoZEP*) (**B**) and flowering (*PoCO*) (**C**), measured in leaves tip, at Maroccone (dark blue), Scalinata (purple) and Rotonda (yellow). Samples were collected every 4 h, starting from seven o’clock in the evening for a total period of 24 h. Data are presented as mean ± SE (number of replicates: *n* = 10) and analysed by Generalized Additive Mixed Models (GAMM) for significance.

We observed that the expression profile of light-dependent genes in a natural night setting (Maroccone) significantly fitted into a mathematical model describing an oscillatory curve only for *PoLHCA4, PoCAB-6A* and *PoPSAG* (Fig. 4A,B, Table 1). Rhythmic oscillation disappeared at the most lit site (Rotonda) in case of *PoCAB-6A* (Table 1). Unexpectedly, a significant oscillatory pattern in *PoSEND33* and *PoPSBS* appeared in Scalinata and Rotonda, where the transcript levels in Maroccone were almost constant throughout the day. Throughout 24 h of monitoring, an oscillatory expression of *PoCO* was observed both in Maroccone and Scalinata but not at Rotonda, where there are intense night light conditions (but the R^2^ adjusted value for the model was very low in this case) (Fig. 4C, Table 1).

**Figure 5:**
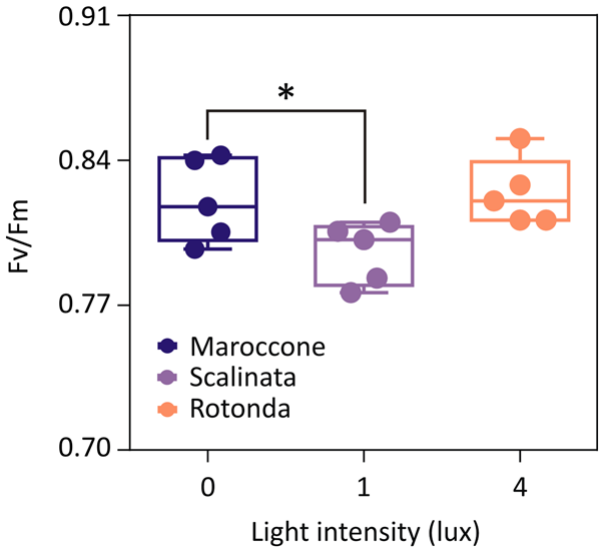
Maximum photosystem II efficiency in *Posidonia* under different nocturnal light conditions. Box plot depicts photosystem II (PSII) efficiency on dark-adapted *Posidonia* leaves (F_v_/F_m_ values), in Maroccone (dark blue), Scalinata (purple) and Rotonda (yellow), where different light intensities at night are indicated in x axis for each site. Data (number of replicates: *n* = 5) were analysed for significance by a linear model (*lm* function of the package *stats* in R).

We then asked whether the observed effects on light-dependent genes might have consequences on in situ photosystem II (PSII) maximum efficiency of *Posidonia*. Data collected in situ on dark-adapted leaves showed a significant decrease in F_v_/F_m_ values measured on plants at Scalinata (artificial night light intensity: 1 lx) in comparison to the control dark conditions in Maroccone (naturally dark condition) (Fig. 5, Table 2). However, we could not detect differences between the darkest and most illuminated site (at night).

**Table 2:** Results of linear models on maximum photosystem II efficiency and growth rate. *p < 0.05, ***p < 0.001.

	Maximum photosynthetic efficiency	Growth rate
Intercept	0.82 (0.01)***	22.06 (2.24)***
Scalinata vs. Maroccone	− 0.03 (0.01)*	− 3.41 (3.03)
Rotonda vs. Maroccone	0.004 (0.01)	− 6.17 (2.85)*
R^2^ adj	0.308	0.13

### *Posidonia* growth is decreased by light at night

**Figure 6:**
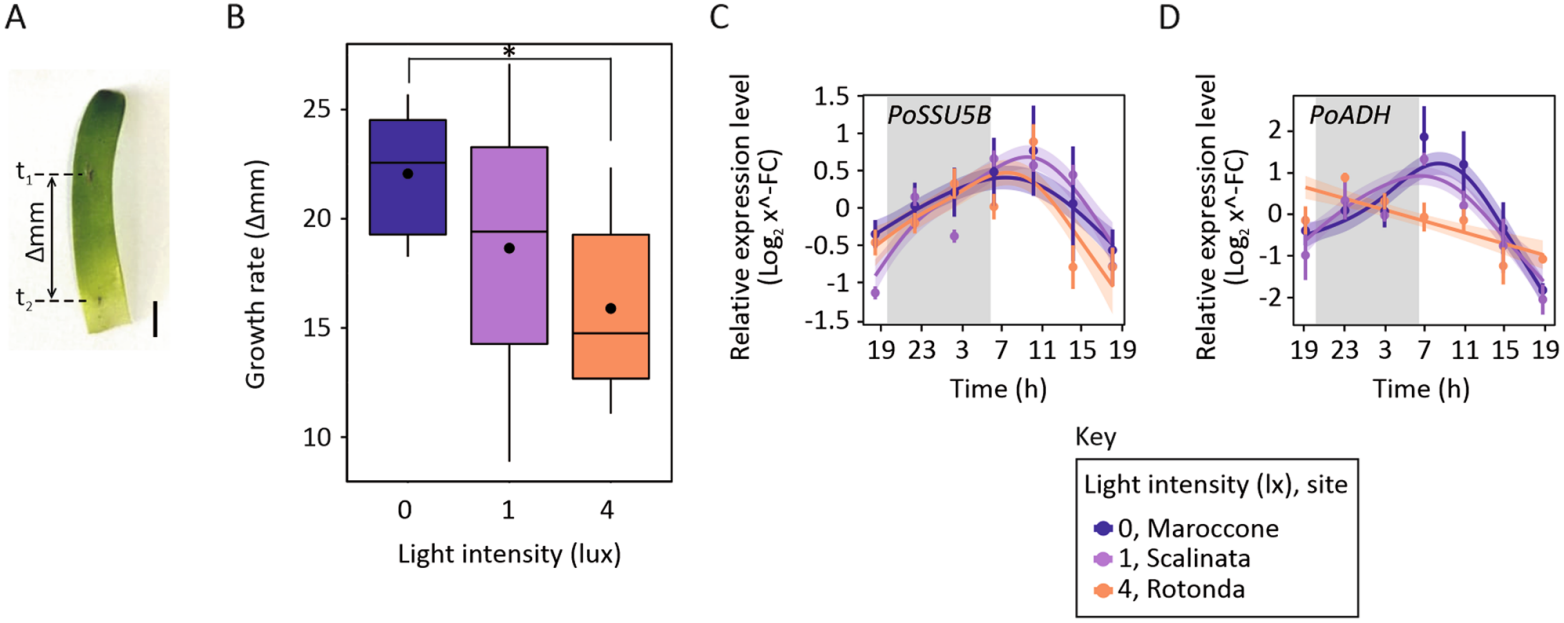
ALAN effect on *Posidonia* leaf growth. (**A**) Example of a young *Posidonia* leaf selected for measurements of elongation, over a period of 14 days. Position of first and second measurements are marked on leaf and labelled as t_1_ and t_2_, respectively. Bar = 2 cm. (**B**) Box plots representing the growth rate (elongation in millimetres over 14 days) in *Posidonia* leaves collected from Maroccone (dark blue), Scalinata (purple) and Rotonda (orange). Measurements were taken as described in (**A**) and data (*n* = 5 for Maroccone, *n* = 6 for Scalinata and Rotonda) were analysed for significance by linear regression. Relative expression levels (Log_2_ x^−FC^) of *PoSSU5B* (**C**) and *PoADH* (**D**) measured in *Posidonia* leaf tips. Samples were collected form Maroccone (dark blue), Scalinata (purple) and Rotonda (orange), every 4 h, starting from seven o’clock in the evening for a total period of 24 h. Data are presented as mean ± SE (number of replicates: *n* = 10) and analysed by Generalized Additive Mixed Models (GAMM) for significance.

We decided to test whether ALAN impacts *Posidonia* physiology when growth conditions are restored to dark nights. Therefore, we measured growth rates of *Posidonia* plants collected from the same three sites where we collected samples for gene expression analyses and measured chlorophyll fluorescence. To this end, the plants were collected from the three sites making sure that they retained a sufficient portion of their rhizome to enable survival, and they were subsequently installed and acclimatised at the bottom of tanks (aquaria) containing sea water from the Maroccone site. We aimed to collect plants when temperature, photoperiod and moon phase were similar to those at the time of our first sampling, for gene expression analyses. This was only possible 2 years later, in summer 2019. We confirmed that values of ALAN and sky brightness during new moon nights were comparable between 2017 and 2019. Indeed, the source of nocturnal urban lighting remained constant during the 2 years, both in terms of spectrum and intensity. The plants were grown for 2 weeks to measure the elongation rate of leaves produced after the transplant. In a controlled environment, with absence of any source of ALAN, plants harvested from Rotonda grew significantly less (with about 35% reduction) than those from Maroccone (Fig. 6A,B, Table 2). In addition to this analysis, we measured the expression of a photosynthesis-related and a stress-related gene in our sample set previously analysed for circadian genes. The transcript abundance of *PoSSU5B*, encoding for RuBisCO small subunit, was not significantly affected at Rotonda (Fig. 6C, Table 1). Intense night lighting negatively affected the gene expression of *PoADH*, resulting in a loss of rhythmicity at Rotonda (Fig. 6D, Table 1).

## Discussion

Our understanding of the impact of ALAN on marine organisms, and primary producers, in particular, is still in its infancy, which is at odds with the global spread of night light pollution along the coastline^8^. In this study we investigated the molecular and physiological response of a key seagrass species, *Posidonia oceanica*, along the NW Mediterranean coastline. ALAN influenced the expression of light-responsive and putative circadian cycling genes at dusk/night in *Posidonia*. Regretfully, the timeframe of 24 h in which it was possible to reliably collect samples did not allow us to validate their *bone fide* association with the plant circadian clock. Nonetheless, we could measure daily fluctuations in gene expression which match those observed for their closest homologs in terrestrial angiosperms^58,59^. The different expression patterns in sites that differ for nocturnal lighting suggested a perturbation of the plant’s clock output. ALAN conditions were associated to reduced growth of the seagrass leaves under controlled conditions.

Light is one of the most important environmental cues to entrain the circadian clock, especially in a marine environment where temperature fluctuations are attenuated in comparison to terrestrial systems. Due to the entrainment of circadian rhythms with the photoperiod, we initially focussed on genes that potentially participate in the internal clock of *Posidonia*. Through a phylogenetic analysis, based on protein sequence similarity across public databases, we identified the closest homologs of *Arabidopsis* circadian proteins in *Posidonia*. We also evaluated the relatedness of these sequences to their closest homologs in species that represent key steps of plant evolution in aquatic and terrestrial habitats (Fig. 2). Sequence similarity among CCA1, GI and ZTL orthologs correlated with the 140–150 million years old separation of angiosperm between monocot and dicot species^60,61^, although this was less clear in the case of the other five genes considered. Instead, we could not find a correlation with the habitats, suggesting that their colonization occurred after the divergence of these sequences in the clades described above and that no differentiation in these coding sequences contributed to the adaptation to terrestrial or aquatic environments^62^.

By monitoring the fluctuation of these putative circadian-clock genes in *Posidonia* for a period of 24 h, we observed that the genes *PoELF3*, *PoLUX1* and *PoZTL* lost or altered their rhythmicity in the presence of dimly bright nights (characterized, in this study, by 1–4 lx) (Fig. 3B). In *Arabidopsis*, the transcription factors ELF3 and LUX1, together with ELF4, constitute a complex that acts during the evening phase of the circadian rhythm, known as evening complex^63^. In particular, ELF3 and ZTL participate to blue light entrainment of the circadian clock both in *Arabidopsis* and rice, where they control circadian outputs as well as feedback regulation of light perception^64–67^. The orthologous blue light receptor ZTL represents an interesting case in *Posidonia*, where its RNA levels fluctuate on a 24 h period, with a peak at the end of the night phase, as observed also in the seagrass *Z. marina* (Fig. 3B)^38^. This is different from what observed in *Arabidopsis*, where ZTL abundance is instead controlled at the post-translational level^68^, and suggests the existence of a feedback mechanism that sensitizes cells to blue light under dark conditions. Since blue light is expected to penetrate the water column the farthest^8^, we speculated that the effect of dim nocturnal light on *PoELF3* and *PoZTL* expression supports their conserved function in blue light signalling. Conversely, ALAN did not affect the fluctuation of the putative morning-loop genes considered in this study.

Prompted by the observed impact of ALAN on the daily rhythmicity of putative circadian clock genes expression, we investigated whether physiological processes known to be synchronized with day length by the circadian rhythm, were also affected under ALAN conditions. The rhythmic abundance of components of the photosynthetic apparatus has been attested from marine algae to flowering plants, and it includes proteins involved in different aspects of the process: oxygen evolution, light harvesting, electron transport and CO_2_ fixation, suggesting a selective pressure in order to preserve this advantageous trait during plant evolution^32,69^. Among the *Posidonia* photosynthetic genes tested in this study, only *PoCAB-6A* was influenced by night lightning, displaying a loss of rhythmicity at the most lit site (4 lx, Rotonda) (Fig. 4A). Previously, *PoCAB-6A* expression has been shown to increase at the end of the day in *Posidonia* plants growing in deep waters^45^, suggesting that the loss of rhythmicity of *PoCAB-6A* in Rotonda could be associated with the misperception of the night period at this site.

Unexpectedly, in case of ALAN (i.e., at Scalinata and Rotonda sites), we attested the acquisition of an oscillatory pattern by *PoSEND33* and *PoPSBS* expression (Fig. 4B). Since *PSBS* is known for its photoacclimation and photo-protective action on the photosystem II^70^, we speculate that the perturbation of the plant’s internal clock might have caused their recruitment to mitigate the repercussions of nocturnal stress on photosynthesis during the day. In land plants, synchronisation of the internal circadian rhythm with the external day–night cycle allows that both carbon fixation during the day, coupled with photosynthesis, and starch consumption during the night occur at the best conditions to optimize plant growth and development^32^. The asynchrony between external and internal clocks has been shown to impair growth and reduce fitness^71–73^. Although we did not observe an effect on maximum photosynthetic efficiency by ALAN at the most lit site, *Posidonia* plants exposed to nocturnal lighting of 4 lx exhibited reduced growth when transferred into controlled growth conditions (aquaria) in the absence of any source of nocturnal lighting, in our laboratory (Fig. 6B). We concluded that *Posidonia* plants adapted to grow under specific and local conditions that characterise their native sites. Since the three sites are sufficiently close to each other, and mainly distinct by ALAN, these results, although preliminary, suggest that high intensity ALAN can exert a long-lasting effect on plant physiology.

The few studies that have investigated the influence of ALAN on terrestrial plants reported temporally variable, species-specific and light source dependent outcomes. In particular, observed effects on growth-related variables were either positive^24,26^ or negative^25^, reflecting the complexity of species-specific processes and mechanisms involved in plant growth. In the model plant *Arabidopsis*, sugar and starch consumption, as well as their allocation throughout the plant, are under the control of the circadian clock; in particular, sugar consumption is regulated antagonistically by the morning protein CCA1 and the evening complex ELF3–ELF4–LUX^74^. Due to the observed effect of ALAN on *PoELF3–PoLUX* pattern of expression, we hypothesise that, similarly to *Arabidopsis*, clock-controlled metabolic pathways might be responsible for the observed growth reduction in the seagrass *Posidonia oceanica*. *PoADH* could have a possible role in this, as we observed that intense nocturnal lighting negatively affected its expression (Fig. 6D). Alcohol dehydrogenase plays an important role in fermentative metabolism which is actuated in response to various abiotic and abiotic stresses, as low oxygen conditions, cold, drought, salt stress and pathogen infection^75–79^. The loss of *PoADH* periodicity observed at the most lit site (Rotonda), could indicate a metabolic impairment and a more susceptibility to other stressors (e.g. cold temperatures, chemical pollutants and pathogens), in response to ALAN.

Flowering is one of the many physiological processes which are under control of the circadian clock^33^. Although the molecular regulation of flowering in *Posidonia* has not been characterised yet, and its occurrence in the Northern Mediterranean is far more variable and rarer than in southern populations, so far only heating has been correlated as an environmental stressor able to alter flowering time^55,80^. In our dataset, we observed that the best *Posidonia* homologous of the *Arabidopsis CONSTANS* was unlikely to be affected by ALAN at any of the study sites (see very low R^2^ value for this analysis in Table 1) (Fig. 4C). However, it should be noted that our experiments were not carried out within the flowering season, starting from September–October, therefore further analyses should be conducted specifically in this period to evaluate whether ALAN affects this aspect of *Posidonia* phenology.

Studies investigating the potential role of ALAN on terrestrial plants highlighted effects on both growth-related and phenological variables in response to different intensities of light, including values of about 20 lx (such as those experienced relatively far from streetlamps), but not as dim as around 1–5 lx^27^. Organisms colonizing shallow coastal areas are usually exposed to a range of artificial light of smaller intensities in comparison to terrestrial species^81,82^. However, they are also adapted to low values of natural light^1^, which makes the potential effect of gradients of dim artificial light on marine autotrophs of scientific concern. Interestingly, a change in photosynthetic biomass have been observed in a planktonic cyanobacteria (*Microcystis aeruginosa*) under an irradiance of about 6.6 lx at night (80 nmol m^2^ s^1^)^83^. Our results showed a relation between an increase in intensity of dim artificial light and the pattern of expression of genes putatively related to the circadian clock in a marine angiosperm colonizing shallow coastal waters. In particular, they suggest an impairment in the mechanisms related to the control of gene expression during the day/night cycle (see *PoELF3*, *PoZTL* and *PoCAB-6A*, Figs. 3 and 4), including the perception of shorter blue wavelengths (see *PoLUX1*, Fig. 3), that are those penetrating deeper underwater and to which many marine species are particularly sensitive. The impairment of the expression pattern of genes regulated by the putative evening-loop genes could be associated with the reduced growth of the seagrass leaves. It is worth noting here that although the selected sites did not apparently differ in any other key biotic or abiotic factors (i.e., depth, wave-exposure, substratum type and inclination, grazing pressure) for the clock itself, other stressors potentially related to the gradient of proximity to the harbour city of Leghorn, might have influenced our results^84,85^, which cannot be, thus, considered as a definitive test of a cause-effect relationship between ALAN and the variables analysed in this study.

Artificial light pollution is currently impinging on more than 20% of world’s coasts, and this percentage is set to increase with the growth rate of coastal human population. The emergent interest on potential impacts of ALAN on marine environments is showing the wide variety of effects related to the disruption of circadian rhythms on different organisms and habitats, from the molecular to the community level. Here we present evidence on the molecular and physiological effects of light pollution on a model seagrass species, *Posidonia oceanica*, which provides habitat and food for invertebrates, fish, mammals, birds and sea turtles, nutrient recycling and sediment stabilization^20^. Seagrasses are key ecosystem engineers in both subtidal and intertidal coastal areas worldwide. A variety of human-related stressors have been recognized as causes of the loss of coastal seagrass meadows and the services they provide, with a rate of disappearance comparable to those of mangrove forests and coral reefs^86^. As ALAN frequently co-occurs with a number of human-related disturbances in urban areas, our results pose key questions about the potential for light pollution to cumulate additively or to interact synergistically or antagonistically with these stressors^87,88^, in order to develop more efficient strategies to preserve seagrass ecosystems in coastal areas.

## Supplementary Information

Supplementary Information 1.

Supplementary Information 2.

Supplementary Information 3.

Supplementary Information 4.

Supplementary Information 5.

Supplementary Information 6.

Supplementary Information 7.

Supplementary Information 8.

Supplementary Information 9.

Supplementary Table S1.

Supplementary Table S3.

## Data Availability

All data generated or analysed in this study are included in this manuscript and its supplementary information files.
