# Supplementary material for: Dim artificial light at night alters gene expression rhythms and growth in a key seagrass species (Posidonia oceanica)
Source: Sci Rep. 2023 Jun 30;13:10620. doi: 10.1038/s41598-023-37261-3 (PMC10313690; doi:10.1038/s41598-023-37261-3)
Supplement: Supplementary file 1 — Supplementary Information 1. [file 41598_2023_37261_MOESM1_ESM.pdf]

```
Consensus                    -------------MEXXSSGEDLXVKXRKPYTITKQRERWTEEEHNRFLEALKLYGRAWQRIEEHVGTKT
PO030913_(PoCCA1)            ------MGFRGVPMETCSSGEELTVKARKPYTITKQRERWTDEEHNRFLEALKLYGRAWQRIEEHIGTKT  64
Zosma76g00390.1_(ZmCCA1)     MMFIDLNHLNLTMDEASSFGEDFNVKARKPYTITKQRERWTEEEHNRFLQALKLYGRAWQHIQEYVATKT  70
XP_010241051.1_(NnCCA1)      -------------MDVYSSGEDLIIKTRKPYTITKQRERWTEEEHNKFLEALRLYGRAWQRIEEHIGTKT  57
Os08t0157600_(OsCCA1)        -------------MEINSSGEEAVVKVRKPYTITKQRERWTEAEHNRFLEALKLYGRAWQRIEEHVGTKT  57
AT2G46830.1_(AtCCA1)         -------------METNSSGEDLVIKTRKPYTITKQRERWTEEEHNRFIEALRLYGRAWQKIEEHVATKT  57

Consensus                    AVQIRSHAQKFFSKLEKEALSKGVXXGQAXDIDIPPPRPKRKPXNPYPRKTCXGTIXSSKEGXXD---XX
PO030913_(PoCCA1)            AVQIRSHAQKFFSKLEKEALSKGVPLGQALDIDIPPPRPKRKPSNPYPRKACMGTIPSSKEGNDESLLPS  134
Zosma76g00390.1_(ZmCCA1)     AVQIRSHAQKFFSKLEKEALSKGVVPGQTINIDIPPPRPKRKPTNPYPRKTCMGTISSSKEGNDKT--SS  138
XP_010241051.1_(NnCCA1)      AVQIRSHAQKFFSKLEKEALSKGIPLGQAIDIDIPPPRPKRKPSNPYPRKTGTGAPTSSPAGVKD---PK  124
Os08t0157600_(OsCCA1)        AVQIRSHAQKFFTKLEKEAINNGTSPGQAHDIDIPPPRPKRKPNSPYPRKSCLSSETSTREVQND-----  122
AT2G46830.1_(AtCCA1)         AVQIRSHAQKFFSKVEKEAEAKGVAMGQALDIAIPPPRPKRKPNNPYPRKTGSGTILMSKTGVND-----  122

Consensus                    XXXSX-----XXXXLXLNXXXXEXAXXXEXSXXXXE-XSEXXNCSEVXTLFXXXPCASXXSXXKSX---
PO030913_(PoCCA1)            VISSC-----QNTALHLKNGACLEKPVDTESSERAKE-VSENGNRLEVLTLFQDAPCASVSSVKKNH---  195
Zosma76g00390.1_(ZmCCA1)     IFSSC-----QNKT-QLSNGLSLENPARSESSEGTEQ-ASENCDCSEVFTLFHNDPCASCTSSVKNC---  198
XP_010241051.1_(NnCCA1)      QLTSGSSLYPGKQMLDLESDPQPEGAAEAEKSARSKE-TMNEENFSEMITLFQEAPCVSMTSSSKSSIAA  193
Os08t0157600_(OsCCA1)        ----------KATISNMTNNSTAQMAGDAALEKLQRKEISEKGSCSEVLNLFREVPSASFSSVNKSS---  179
AT2G46830.1_(AtCCA1)         ----------GKESLGSEKVSHPEMANEDRQQSKPEEKTLQEDNCSDCFTHQYLSAASSM---NKSCIET  179

Consensus                    ----SNPXXXRXFXPXXXEXXXXXXXEXDXXSXXAXXNXX------XDXXDXDXXRLXGXXXXSXXXXSX
PO030913_(PoCCA1)            ----ATPPSFRKFVPFLKEINHK--LESDKASLTANENEELTQCT-ADCTDLDIGRIKRLHTNSQ-TKVA  257
Zosma76g00390.1_(ZmCCA1)     ----GNPSSIKKFIPILEDINHTSEMIKDRNSQIAIDNKEAMQYTDNDC-DVDIGRLEGIKKSNLNTNSA  263
XP_010241051.1_(NnCCA1)      PLVLSQPCTLRESTPSMKEMKDRGVIEESYLTVKAKKNKK------PGTADADHTDLNGV---SEPPNSG  254
Os08t0157600_(OsCCA1)        ----SNHGASRGLEPTKTEVKDVVILERDSISNGAGKDAK-------DINDQEMERLNGIHISSKPDHSH  238
AT2G46830.1_(AtCCA1)         ----SNASTFREFLPSREEGSQNNRVRKESNS-----------------DLNAKSLE------------  215

Consensus                    XXXXXX-----------XXKXXXXXXXXSNXXXQXXXXXSXHXXXHVXX---XXXGXXXX--XSXXT---
PO030913_(PoCCA1)            PMKMSP-----------GHKQPHEVGILSNGELQGTRSNSKHVAFHSVEENNENSGHGANFKPSNVT---  313
Zosma76g00390.1_(ZmCCA1)     SMEKKA-----------KHKKPD--EATSNCLMQDTNERSKKQKCCVQS-------------NSQI----  303
XP_010241051.1_(NnCCA1)      ESCLNSEDQQVKGERKDGRKESEKLDPQSKNEMQTNQNYPRHVPVHVVD---GISGPCMQIPSPDMTYPA  321
Os08t0157600_(OsCCA1)        ENCLDT--------------SSQQFKPKSNSVETTYVDWSAAKASHYQMDRNGVTGFQAT--GTEG----  288
AT2G46830.1_(AtCCA1)         -------------------------NGNEQGPQTYPMHIPVLVPL-------------GSSIT---  240

Consensus                    ----------SXXXXXXXXX-------PXXXXPXXPXXPEXXXXXXXXSFXXXXPXF-PPFTXXHXNQDX
PO030913_(PoCCA1)            -------ATSSQAEAPVYSI-------PPFTNPSLSATPEYSTHPPLSSFHPELPIF-PPFTLFHNNQEA  368
Zosma76g00390.1_(ZmCCA1)     ----------SQSDIHVCHI-------PELINESMPAKPEHHTKST-TSFNQEHPSS-PPFTFFHGNQDV  354
XP_010241051.1_(NnCCA1)      FIIHQLGGIHGNPASVIHQVGGAHGN-PKFMDPTASIINEHQSNASRSSFHQPLPTFPPPFTPLCNNQDS  390
Os08t0157600_(OsCCA1)        ----------SHPDQTSDQMGGASGTMNQCIHPTLPVDPKFDGNAAAQPFPHNYAAF-APMMQCHCNQDA  347
AT2G46830.1_(AtCCA1)         ---------------------------SSLSHP--PSEPDSHPHTVAGD---------------------  260

Consensus                    YRSXXNXSSTFSNXIXSTLLQNPAAHXAAXLAASFWPXXXGXXPXSXXXXXXXXXXXXXXXXXPNMAAIA
PO030913_(PoCCA1)            YRSLHNISSTFSNFIMSTLLENPAAHSAACLASSFWPSVDGEIINSASEALNSGILQRQVNASPNMAAIA  438
Zosma76g00390.1_(ZmCCA1)     YRSLINMSSSFSNCILFCLQQNPSAHVAANLAASVWHCLEGDGMNSTAKVVDGGNYPNNAYTAPNMVAIA  424
XP_010241051.1_(NnCCA1)      YRSFINISSTFSSLVVSTLLQNPAAHAAASLAASFWPCTNADAPLESPGTV-GEFQARHMSPSPSLAAIA  459
Os08t0157600_(OsCCA1)        YRSFANMSSTFSSMLVSTLLSNPAIHAAARLAASYWPTVDGNTPDPNQENLSESAQGSHAGSPPNMASIV  417
AT2G46830.1_(AtCCA1)         YQS-------FPNHIMSTLLQTPALYTAATFASSFWP------PDSSGGS------PVPGNSPPNLAAMA  311
```

```
Consensus                   AATVAAASAWWATHGLLPLCXPPXSXGFXXAPXXXXXXPXXXXXXXXXXXXXKDXXXXXXQXXXXQDXX-
PO030913_(PoCCA1)           AATVAAATAWWATHGLFPFCHPPLSTGFSFASTPSATGPTVNNRSPEEREARKD---DRAQLHRFQDEH-   504
Zosma76g00390.1_(ZmCCA1)    SATVAAATAWWATHGLHTFPHPH-STDLNFA----GVAPIITNPVSEGREVRKD---DSVQFQAQQDER-   485
XP_010241051.1_(NnCCA1)     AATVAAASAWWAAHGLLPLC-PPLHPGFTCIPPYTAATPSTDTVQPPVIN--KEKMENTLQDPPWKDQ--   524
Os08t0157600_(OsCCA1)       TATVAAASAWWATQGLLPLFPPPIAFPFVPAPSAPFSTADVQRAQEKDIDCPMDNAQKELQETRKQDNFE   487
AT2G46830.1_(AtCCA1)        AATVAAASAWWAANGLLPLCAPLSSGGFTSHPPSTF-GPSCDVEYTKASTLQHGSVQSREQEHSEASKA-   379

Consensus                   ----ILDSEXXEXXXXXXSXSKSXXXSSSD--XXXXXXGX------------XXXGX-----KXVDRSS
PO030913_(PoCCA1)           ----ILDPESPDASVMQHSSSKSLPLTSSD--S------D------------ERLGG-------------   537
Zosma76g00390.1_(ZmCCA1)    ----ILDSESPQTAVLQCSTSKS--TSSSD--CGESKRGD------------EVIGGNTTKSDRKVNCYS   535
XP_010241051.1_(NnCCA1)     ----QLDPEFSEALETRRLDSKSPPLSSSDSESGGVRSNNELKVQVQKPVLVNGFLDSKIKTRKQVDRSS   590
Os08t0157600_(OsCCA1)       AMKVIVSSETDESGKGEVSLHTELKISPADKADTKPAAGAETS---------DVFGN-----KKKQDRSS   543
AT2G46830.1_(AtCCA1)        --RSSLDSEDVENKSKPVCHEQPSATPESD------AKGS------------DGAGD-----RKQVDRSS   424

Consensus                   CGSNTPSSSXXXXXXAXXXXEX----XXXXXXXXXXXXXXXXEXNXRRXRXSXXXXDSWKEVSEEGRLAF
PO030913_(PoCCA1)           -------------------------------------------------------------------IEL   540
Zosma76g00390.1_(ZmCCA1)    YGSSKLSSTEKYTNVLLKNTESQTLEQVMKSNKLE-NQTILSEANGQRIKGSMVVNDSWKEVSEEGRLAF   604
XP_010241051.1_(NnCCA1)     CGSNTPSGSEVETD-ALKKHEK----EEEDSKEPDLSHPSA-EPNNRRSRNTSNTNESWKEVSEEGRLAF   654
Os08t0157600_(OsCCA1)       CGSNTPSSSDIEADNAPENQEK----ANDKAKQASCSNSSAGDNNHRRFRSSASTSDSWKEVSEEGRLAF   609
AT2G46830.1_(AtCCA1)        CGSNTPSSSDDVEADASERQEDGTNGEVKETNE-DTNKPQTSESNARRSRISSNITDPWKSVSDEGRIAF   493

Consensus                   XALFSREVLPQSFSXXXXXXX-------KEXXX-XEXDXXXXXXLDLXXXX--XXXDXXXXXXN------
PO030913_(PoCCA1)           NSSYARE-------------------------------------LDLKH---------------------   552
Zosma76g00390.1_(ZmCCA1)    EALFSREVLPQSFSLTGLTAMGMTPRNLKMGNV-KAGDAKRKVSADVVA-------DNDVDMGN------   660
XP_010241051.1_(NnCCA1)     QALFSREVLPQSFSPPHDSKEPPKCTNEKEKQKSDEKDKDDKLQLDLCCKTWEASPERPGTKKNELLISS   724
Os08t0157600_(OsCCA1)       DALFSRERLPQSFSPPQVEGS-------KEISK-EEEDEVTTVTVDLNKNA--AIIDQELDTADE-----   664
AT2G46830.1_(AtCCA1)        QALFSREVLPQSFTYREEHRE-------EEQQQ-QEQRYPMALDLNFTAQL--TPVDDQEEKRN------   547

Consensus                   -----XXXXXXXL-XXXKLXSR-RTGFKPYKRCSXEAKEXRXXXX-X--XXXEKGX-KRXRLEXEASTX
PO030913_(PoCCA1)           -----------------------DTGFHDY------------------------GN----KAESEKKTR   570
Zosma76g00390.1_(ZmCCA1)    ------SRMKCKLNGNLNLE---RSGFKPYKRCSMDAKKGTTSQG----QGDEKGSRKRLRLEGETTAR   716
XP_010241051.1_(NnCCA1)     GSTTETGLLITEL-GHAKLKSR-RTGFKPYKRCSVEAKENRVANAYS--QGEEKGP-KRIRLEGEASTS   788
Os08t0157600_(OsCCA1)       ----PRASFPNEL-SNLKLKSR-RTGFKPYKRCSVEAKENRV----P--ASDEVGT-KRIRLESEAST-   719
AT2G46830.1_(AtCCA1)        -----TGFLGIGL-DASKLMSRGRTGFKPYKRCSMEAKESRILNNNPIIHVEQKDP-KRMRLETQAST-   608
```

Consensus Threshold: >50%

Compare to: the consensus

Amino acids that match the reference are marked with yellow highlighting.

Created: 12 Apr 2023

Last Modified: 12 Apr 2023
