# Supplementary material for: Dim artificial light at night alters gene expression rhythms and growth in a key seagrass species (Posidonia oceanica)
Source: Sci Rep. 2023 Jun 30;13:10620. doi: 10.1038/s41598-023-37261-3 (PMC10313690; doi:10.1038/s41598-023-37261-3)
Supplement: Supplementary file 2 — Supplementary Information 2. [file 41598_2023_37261_MOESM2_ESM.pdf]

```
Consensus                          --------------XXXXXXXXXXXXXXXXXARXCDXCXSXPAXXXCRADSAFLCXXCDXXVHXANXXAS
Mapoly0110s0028_(MpCO-like)        ------------------------------MPKPCDACQSSSAVVYCRADAAFLCAGCDTKVHGANKLAQ   40
SELMODRAFT_185898_(SmCO-like)      ------------------------------MTKPCDACQSGNAVIYCRADAAFLCCSCDNKVHCANKLAS   40
PO020219_(PoCO-like_1)             --------------MASEAVEREGKSYWGMGARECDSCRASPALLFCRADSAFLCGACDSKVHGANKLAS   56
PO000075_(PoCO-like_2)             ------------MAGRGTELLAEKKSYWAMAARECDSCRSTPALLFCRTHSAFLCGPCDAGVHAVTAVSS   58
Zosma123g00020.1_(ZmCO-like1)      -----------------MTAEDGKKSYKGERGRECDYCKISPAILFCHPDSAFLCSKCDSSVHEANKLAS   53
Zosma218g00180.1_(ZmCO-like2)      ------------------------------MARECDFCKVSPAFLFCRADSAFLCSHCDSNIHGANKLAS   40
XP_010257847.1_(NnCO-like1)        -------------MMLKEEGSSGGS--RGGWARMCDSCRSAACAVFCRADSAYLCAGCDARIHAVNQVAS   55
XP_010261698.1_(NnCO-like2)        -------------MMIKEEGGSGGTGGGGGWARMCDSCCSAPCAVYCRADSAYLCAGCDARIH--DQVVS   55
LOC_Os06g16370.1_(OsCO/HD1)        MNYNFGGNVFDQEVGVGGEGGGGGEGSGCPWARPCDGCRAAPSVVYCRADAAYLCASCDARVHAANRVAS   70
AT5G15840_(AtCO)                   --------------MLKQESNDIGSGENN-RARPCDTCRSNACTVYCHADSAYLCMSCDAQVHSANRVAS   55

Consensus                          R-HERVWXCEVCEXAPAAFTCKADAAALCXXCDXDXHSANPLARRHXRVPIXPFXXXXXXXXXXXXXXXX
Mapoly0110s0028_(MpCO-like)        R-HERVWVCEVCEVAPAVVTCKADAAALCVACDTDIHSANPLARRHERTPVTPFYECPGQSIKVAH---I  106
SELMODRAFT_185898_(SmCO-like)      R-HERVLVCEVCEHAPAAVTCKADAAALCVTCDSDIHSANPLARRHERVPITPFVDSSDGGAAPPPAPPI  109
PO020219_(PoCO-like_1)             R-HERVWMCEVCEQAPAAVTCKADAAALCVSCDSDIHSANPLARRHERVPIVPFFDSTTKNAAAKPPMSS  125
PO000075_(PoCO-like_2)             SPHERVWMCEVCEQAPAAFTCKADAATLCAPCDADVHSANPLARRHDRAHVVPFFHSPANTTANPNPNTA  128
Zosma123g00020.1_(ZmCO-like1)      R-HDRVQMCDVCENAPAAFTCKADSASLCVTCDSEVHSANPLARRHDRVPVVPFSK-PSQIMNDVHGDED  121
Zosma218g00180.1_(ZmCO-like2)      R-HERVWICEVCEQAPAAFTCKADAAVLCATCDADVHSANLLSVRHERVPITPFFDSPSPNMTRKGRTTG  109
XP_010257847.1_(NnCO-like1)        R-HERVWVCEACERAPAAFTCKADAAALCTTCDADIHSANPLARRHHRVPILPIAGCLYGPSPTNPGRRV  124
XP_010261698.1_(NnCO-like2)        Q-HERVWICEVCERAPAAFTCKADAAALCTTCDADIHSANPLARRHHRVPILPISGCLYGPSGMNPGRPV  124
LOC_Os06g16370.1_(OsCO/HD1)        R-HERVRVCEACERAPAALACRADAAALCVACDVQVHSANPL------------------PAITIPATSV  121
AT5G15840_(AtCO)                   R-HKRVRVCESCERAPAAFLCEADDASLCTACDSEVHSANPLARRHQRVPILPISGNSFSSMTTTHHQSE  124

Consensus                          XXXXXXX-------------XXXXXXXXDXXXXXE---------------XXXEXASWLLXXX-------
Mapoly0110s0028_(MpCO-like)        SALVAPQ-------------GGQGRDDDADLDADE-------------HSVAEEAASWLLPNPKPLTENN  150
SELMODRAFT_185898_(SmCO-like)      L-------------------HDTGNANHDDEEESS----------------AAEAASWLLPQP-------  137
PO020219_(PoCO-like_1)             GPLLRKQQ------------DEDFEDDDDKAEEDE---------HEEEAEAEAEAESWLLPKE-------  167
PO000075_(PoCO-like_2)             IDFLLKR-------------TDDFYEEKEMEGPEM-----------------------------------  150
Zosma123g00020.1_(ZmCO-like1)      --------------------DENGYDESDGRNVKE---------------EEKRMNDFYYY---------  147
Zosma218g00180.1_(ZmCO-like2)      --LIRKNRCQGDHHDVGDVDEEEVEDDDDDNEEEEECNNEDGNKEESDAVAEAEAASWLIPND-------  170
XP_010257847.1_(NnCO-like1)        RPDINIE-------------DGFMTQEGDEDIDEE---------------DEDETASWLLLNP-------  159
XP_010261698.1_(NnCO-like2)        RPEINIE-------------DEFMTQEADEEDEDE-------------------AASWLLLNP-------  155
LOC_Os06g16370.1_(OsCO/HD1)        LAEAVVA-------------TATVLGDK-----------------------DEEVDSWLLLSK-------  148
AT5G15840_(AtCO)                   KTMTDPE-------------KRLVVDQEEGEEGDK---------------DAKEVASWLFPNS-------  159

Consensus                          ---X---------------------XXXXX---------------------XX--------XXXXGXLF-
Mapoly0110s0028_(MpCO-like)        ILGDDKGSMGQGEMGSLVNDDGDDDDLEPSSFLVPSGGFSPNSTLSHKSDGMSMKHHKSKVEANAGDLF-  219
SELMODRAFT_185898_(SmCO-like)      ---NNLAKSDGEKLGGGVESTDFYSTLKPS--------APPPLRIEKLLLKSQ--------AAANFDLF-  187
PO020219_(PoCO-like_1)             ---N---------------------DVKPL--------------------------------LVDQYFF-  180
PO000075_(PoCO-like_2)             -------------------------KMKVD---------------------LH--------DHHDQYLF-  165
Zosma123g00020.1_(ZmCO-like1)      -------------------------EMKPT--------------------------------TEGGYFF-  159
Zosma218g00180.1_(ZmCO-like2)      ---G----------GGVHSNVEIKYEMKPS---------------------TE--------RMAEQYYF-  197
XP_010257847.1_(NnCO-like1)        ---G---------------------KNDNN-------------------------------QNNNGFLLG  174
XP_010261698.1_(NnCO-like2)        ---V---------------------KNNNQ--------------------------------NNSGLLFG  169
LOC_Os06g16370.1_(OsCO/HD1)        ---D---------------------SDNNNNNNNNNDN------DNNDNNNSN--------SSNNGMYF-  179
AT5G15840_(AtCO)                   ---D---------------------KNNNN--------------------------------QNNGLLF-  172

Consensus                          XXVDXYLDL-DYXXXXDX----------------------XXXXXXXXXXXXXDSVVP------------
Mapoly0110s0028_(MpCO-like)        SDVDPYLDL-DYATVVG-----------------------------ANPNLGADSLVP------------  247
SELMODRAFT_185898_(SmCO-like)      SDEDSYLDM-DFLGAL---------------------------------HSVTDSLVP------------  211
PO020219_(PoCO-like_1)             QDVDPYLDL-DYAASMD---------------------------PRFHQ----DSVVPVQSRGV---AVG  215
PO000075_(PoCO-like_2)             SAVDPYLDL-EYGSSVEN--------------------------TRFHHH--ADSVVP------------  194
Zosma123g00020.1_(ZmCO-like1)      SDVDSYLDL-DYAASMD-----------------------QNCHPHQHHHMTADSVVP------------  193
Zosma218g00180.1_(ZmCO-like2)      PEVDPYLDL-DYAASMD---------------------------PKFYRSAAADSIVPVQNRNITAPAAG  239
XP_010257847.1_(NnCO-like1)        GEVDEYLDLVDYSSCTDNQY-N-----------------QQQHYSVQHKNDGSDSVVP------------  214
XP_010261698.1_(NnCO-like2)        GEVDEYLDLVDYNSCTENQY-ND-------------HYYQQNQCSVQQKNEGSDSVVP------------  213
LOC_Os06g16370.1_(OsCO/HD1)        GEVDEYFDLVGYNSYYDNRIENNQDRQYGMHEQQEQQQQQQEMQKEFAEKEGSECVVP------------  237
AT5G15840_(AtCO)                   --SDEYLNLVDYNSSMDYKFTGE-------------YSQHQQNCSVPQTSYGGDRVVP------------  215
```

Sequence	Alignment	Position
**Consensus**	---XVXXXXXXXXXPXXXXXXX-----XXXDXXXS-XXXXS-YXXSXXHSXSXSSXEXGVVPDXXXXXXX	
Mapoly0110s0028_(MpCO-like)	----VHSDHPAHSSPSMSTGSG------SFEGEAVCKAGYG-YESSLTHSMSSSSLDVGIVPDTNALSDI	306
SELMODRAFT_185898_(SmCO-like)	--IHTTGGALHSSSPVGSNADS-----YDLDVHDK-SPPHA-YCPGL--SLSASSIDVGVVPDASLSDIS	270
PO020219_(PoCO-like_1)	FGIGVAAVHHHPPPPFIAATVP--DGSYDIDFARSKPHSYP-HAPSLSRSVSSS--EVGVVPDGSAMTDV	280
PO000075_(PoCO-like_2)	---AVQNISVPPPPPAHDHHVHFFGGGYDLDFMNS-NQTCS-YAPSLGHSVSS---EVGVVPEESTATDM	256
Zosma123g00020.1_(ZmCO-like1)	-GIAVGVAASFQPPPSFFSTVS--------DYSYS---PYS-TAASFTHSVSSS--EVGVVPNSGSTTTE	248
Zosma218g00180.1_(ZmCO-like2)	MAVGVAAMHHHHPPPFISNISD---RSYDIDITRSSNHSYS-YAPSVSHSMSSS--ETGVVPDGAMASAS	303
XP_010257847.1_(NnCO-like1)	----VQCLAVKEEQ--QQQNLH-----LDMDYDAS-KPGFS-Y----SHSVSLSSMDASVVPDAMMNDIS	267
XP_010261698.1_(NnCO-like2)	----IQCLAVKEN----QHNLQ-----LEMDYEAS-KPGFS-YTASLSHSVSFSSIDASVVPDGIMNDIS	268
LOC_Os06g16370.1_(OsCO/HD1)	----SQITMLSEQQHSGYGVVG-------ADQAASMTAGVSAYTDSISNSISFSSMEAGIVPDSTVIDMP	296
AT5G15840_(AtCO)	----LKLEESRGHQCHNQQNFQ-----FNIKYGSS-GTHYN-DNGSINHNAYISSMETGVVPESTACVTT	274

Sequence	Alignment	Position
**Consensus**	XXXXXXXXGXXX-----XXXXXXXXX--XXXXXXMDREARVLRYREKRKXRKFEKTIRYASRKAYAETRP	
Mapoly0110s0028_(MpCO-like)	STPYDGSQGHGF-----EFPPRMLHM--AQVEPPMAREARVMRYKEKRKNRKFEKTIRYASRKAYAESRP	369
SELMODRAFT_185898_(SmCO-like)	TPQSRPTSSSVFGSGEAQAAAAPLHH--ATPLEPIAREARVLRYREKRKNRKFEKTIRYASRKAYAETRP	338
PO020219_(PoCO-like_1)	SNPYAGAVNQAS-----MAA--------AMAAVGMDREARVLRYKEKRKNRRFAKTIRYASRKAYAETRP	337
PO000075_(PoCO-like_2)	TSNSYDQYGLTV-----NQT----AV--ATVATGTDREARVMRYREKKKNRKFEKTVRYASRKAYAETRP	315
Zosma123g00020.1_(ZmCO-like1)	----------------------------ISAAVGLDRKARVLRYREKRKNRRFEKTIRYASRKAYAEIRP	290
Zosma218g00180.1_(ZmCO-like2)	----------------------------ASAVVGMDREARVMRYREKRKTRRFEKTIRYASRKAYAETRP	345
XP_010257847.1_(NnCO-like1)	NSHSRPPKGTID-----LFSS-PLAM--PPQFTPMDREAKVLRYREKRKTRKFEKTIRYASRKAYAETRP	329
XP_010261698.1_(NnCO-like2)	NSHSRPPKGTID-----LFSSPPLPM--PPQFTPMDREAKVLRYREKRKTRKFEKTIRYASRKAYAETRP	331
LOC_Os06g16370.1_(OsCO/HD1)	NSRILTPAGAIN-----LFSGPSLQM--SLHFSSMDREARVLRYREKKKARKFEKTIRYETRKAYAEARP	359
AT5G15840_(AtCO)	ASHPRTPKGTVE-----QQPDPASQMITVTQLSPMDREARVLRYREKRKTRKFEKTIRYASRKAYAEIRP	339

Sequence	Alignment	Position
**Consensus**	RIKGRFAKRTXX----XXX---VDXXXX-------XXXXXDXG-YGVVPSF	
Mapoly0110s0028_(MpCO-like)	RIKGRFAKRNPT----DMS---LQDMYP---------NVPDSG-FGVVPAF	403
SELMODRAFT_185898_(SmCO-like)	RIKGRFAKRGEM------------DSYD-------------AS-FGVVPSF	363
PO020219_(PoCO-like_1)	RIKGRFAKRTELEGHGDVI---VDSLYGSPAAAAAAGLMVDPG-YGVVPSF	384
PO000075_(PoCO-like_2)	RVKGRFVKRTELDCEGDADAAVVDSIYA-SSATAVAALMVDAG-YGVVPSF	364
Zosma123g00020.1_(ZmCO-like1)	RIKGRFAKQTMI---IGMD---VDDENN---------NAIDSG-YGVVPSF	325
Zosma218g00180.1_(ZmCO-like2)	RIKGRFAKRTEM--EDVMG---DDDMYPPSQGNATASLIVDRPNYGLVPSF	391
XP_010257847.1_(NnCO-like1)	RIKGRFAKRTDV----EVE---VEQMFS-------TTVLAESG-YGVVPSF	365
XP_010261698.1_(NnCO-like2)	RIKGRFAKRTDV----EVE---VDQMFS-------TTVLAESG-YGIVPSF	367
LOC_Os06g16370.1_(OsCO/HD1)	RIKGRFAKRSDV----QIE---VDQMFS-------TAALSDGS-YGTVPWF	395
AT5G15840_(AtCO)	RVNGRFAKR-EI----EAE----EQGFN-------TMLMYNTG-YGIVPSF	373

Consensus Threshold: >50%

Compare to: the consensus

Amino acids that match the reference are marked with yellow highlighting.

Created: 12 Apr 2023

Last Modified: 12 Apr 2023
