# Supplementary material for: Dim artificial light at night alters gene expression rhythms and growth in a key seagrass species (Posidonia oceanica)
Source: Sci Rep. 2023 Jun 30;13:10620. doi: 10.1038/s41598-023-37261-3 (PMC10313690; doi:10.1038/s41598-023-37261-3)
Supplement: Supplementary file 3 — Supplementary Information 3. [file 41598_2023_37261_MOESM3_ESM.pdf]

```
Consensus                        ---MKXGX-------X---------XXKXXGPLFPRLHVNDAXK-GGPRAPP-RNKMALYEQLXXPSXRF-
Mapoly0014s0139.1_(MpELF3)       MMGTKDGKGDGANHRGLTGSGAGANGVETGALFPRLHVKETKR-VGPRAPP-RNKMALYEQFTFPSYRFV    68
SELMODRAFT_415241_(SmELF3)       ---MEDK-----------------NAPPGGALFPRLHVKETKN-AGPRAPP-RNKMALYEQLTIPSHRF-    47
PO041070_(PoELF3)                ---MKRGR-----EEG--------DGKVMSALFPRLHVNGADT-GGPRAPP-RNKMSLYEQLSVPIQRS-    51
Zosma3g01990.1_(ZmELF3)          -----------------------------MFPRLQVKDADKGGGPRPPP-RNKMALYEQLSVPSKRF-    37
XP_010244364.1_(NnELF3-1)        ---MKGGK-------D--------EEKIMGPLFPRLHVNDADK-GGPRAPP-RNKMALYEQLSIPSQRF-    49
XP_010267078.1_(NnELF3-2)        ---MKRGR-------D--------EEKITGPLFPRLHVNDTEK-GGPRAPP-RNKMALYELLSIPSQRF-    49
XP_010267081.1_(NnELF3-3)        ---MKRVG-------D--------EEKIMGPLFPRLHVNDTEK-GGLREPPRRKMTLYEQLNISSQGL-    50
Os06t0142600_(OsELF3-1)          -MATRGGGGGGGKEA---------KGKVMGPLFPRLHVNDAAKGGGPRAPP-RNKMALYEQFTVPSHRF-    59
Os01t0566100_(OsELF3-2)          ---MRGGGGGKEVEE---------RGKVMGPLFPRLHVNDAAKGGGPRAPP-RNKMALYEQFTVPSHRF-    57
AT2G25930.1_(AtELF3)             ---MKRGK-------D--------EEKILEPMFPRLHVNDADK-GGPRAPP-RNKMALYEQLSIPSQRF-    49

Consensus                        -------XXXX---XXXXXXXXXXXXX----------------XXXSXSXSQXXGXXRXXXXP------
Mapoly0014s0139.1_(MpELF3)       PPAVMLPTSGLS--AGSSPYSPHVHSNGEKMALYEQFTFPQHRLVQPSTSFPSSGLPSRSCSSSLQQSSS   136
SELMODRAFT_415241_(SmELF3)       -------QQQQSDGAPPQPFVPQ-VCK----------------FEPMISP----ALNRGYYFP------    82
PO041070_(PoELF3)                -------SNS--------------RVCL---------------TTSS----EECGQERGLFPP------    74
Zosma3g01990.1_(ZmELF3)          -------N----------------HAAA---------------------------------------    42
XP_010244364.1_(NnELF3-1)        -------NSGL---APTLPLPPNNASN----------------LVPSTSSSQGGAHERSVFSQ------    86
XP_010267078.1_(NnELF3-2)        -------NPRL---APTLPLPPNNSCT----------------SVSSISSSQSGGHERSVLSP------    86
XP_010267081.1_(NnELF3-3)        -------NSSL---APTLSLLPDNACT----------------SVPSISLSQGGGHERSVFSP------    87
Os06t0142600_(OsELF3-1)          -------SGGGGG-GGVGGSPAHSTSA----------------ASQSQSQSQVYGRDSSLFQP------    98
Os01t0566100_(OsELF3-2)          -------SGG----GGALASARGSLAR----------------STSAASQSQVYGCDMPLFEP------    93
AT2G25930.1_(AtELF3)             -------GD-----HGTMNSRSNNTST----------------LVHPGPSSQPCGVERNLSVQ------    84

Consensus                        -----XX-VXXX--XXXXXXXXXXXXXX-XXX-XGXXXXXXRXSXXXXXXXPXXXXXXXXXXXXX----
Mapoly0014s0139.1_(MpELF3)       NDGSRYY-CPPPYGSLGMPYMAMNVPHQAGQPVVNGSATSTVVDSESEIQRTPSTTATASGGVSSHGVGF   205
SELMODRAFT_415241_(SmELF3)       -----YYMVPSP------------------------------------VAFQPMNV---VLGQHSQ----   104
PO041070_(PoELF3)                -----HN-VQSK--LPSSSFDKVHSHFSN--TMNGNSVGMLERRSSKHTSHVPSYVSRLLRSAADC----   130
Zosma3g01990.1_(ZmELF3)          -----------------------------------------------------------ASIARC----   48
XP_010244364.1_(NnELF3-1)        -----IY-VSPP--TP-------HSRSSDGANL-NGTVAECDRKSTKNANYRISNATRNLLSTNDC----   136
XP_010267078.1_(NnELF3-2)        -----FY-LQPP--TSTHSAGKLHSSSSDGANL-NGTMAEFDRKSTKNVNHRPSNATTGLLSTSDR----   143
XP_010267081.1_(NnELF3-3)        -----FY-VPPP--TPTHSSEKLH-PCFDGVNL-SGAMAEFDRKSTTNTNNRPSNVTMGLLSTNDR----   143
Os06t0142600_(OsELF3-1)          -----FN-VPSN--RPGHSTEKINSDKIN-KKI-SGS-----RKELGMLSSQTKGMDIYASRSTAE----   149
Os01t0566100_(OsELF3-2)          -----FN-VPSN--GPGQSVEKMNSNSVN--RQINGS-----RKDSGMLSTQPKGIDKYGSGSRAE----   144
AT2G25930.1_(AtELF3)             -----HL-------------------------------------DSSAANQATEKFVSQMSFMENVR----   109

Consensus                        XSXXXXXXXXXXXXXXXXXX-XDXXXVPXXXXSXXXXX------------SXXX-PXXXXXX-XXPXX
Mapoly0014s0139.1_(MpELF3)       GSFRPSSHANLRTSSSKKGGRINDDCTVPIYTAPKSSTGFPKVYSEGSDWSSGGPWQPGIDLTKACQPMP   275
SELMODRAFT_415241_(SmELF3)       SVANETEQRELQQVPGDGGSN-RQLARIPS-LHSRAGSG-----------KTKTRAPRVD----DVAAV   156
PO041070_(PoELF3)                SYFYPDNYTNSET-CGNRCEE-KGSL-----IHANRSPK-----------TDMV-IPSL-----SSQLQ   175
Zosma3g01990.1_(ZmELF3)          SS----------------LSA-DKLLILP------------------------------------------   60
XP_010244364.1_(NnELF3-1)        NSCHRPDFSNLKNYCGDKLED-EDDLRVPTYIHSGVASC-----------SNKD-LPNPNGER-CTPLS   191
XP_010267078.1_(NnELF3-2)        SSCHSHAFSNLRK---RIPGD-EDDLRVPVFVHSQVSPC-----------SDKD-IPNTSRER-FSPFI   195
XP_010267081.1_(NnELF3-3)        NSCCSHALSNSRK---QKPGD-EDDLRAPAFVHSRVSLC-----------SDKD-LPNTSRER-FSPFI   195
Os06t0142600_(OsELF3-1)          -APQRRAENTIKSSSGKRLAD-DDEFMVPSVFNSRFPQY-----------STQE-NAGV-QDQ-STPLV   202
Os01t0566100_(OsELF3-2)          CAPQQRVEKGIKSSSGRKLAD-DDEFIVPSVFSARFPQY-----------STKE-RAGVQEE--STPLV   198
AT2G25930.1_(AtELF3)             SSAQHDQRKMVR--------E-EEDFAVPVYINSRRSQS-----------HGRT-KSGIEKEK-HTPMV   156

Consensus                        XXXXXXS-XXXXXXXPXKXX--XXXXXXXXXXNXXXXXXXXXXXXDXXSXXXXRXXXXXXXXXXXXXXX
Mapoly0014s0139.1_(MpELF3)       VSYDYQVNSSGPSNSRGDCREESVTGVTGEHRETKIVYSERGVGEVTELPDGTSREAYKEKQVSSLRNRH   345
SELMODRAFT_415241_(SmELF3)       PTYPSK-----PAAVPARSSKQEHFASASVAPSNASTSATKSQRQVEVRETTQARKDSR-----------   210
PO041070_(PoELF3)                SSH---------DKVPGKC----------------------RSADLQSKGHQINNDDQDVA-------   205
Zosma3g01990.1_(ZmELF3)          ----------------------------------------------------QKKNTDGNL--------   69
XP_010244364.1_(NnELF3-1)        PTHPSPS-KLAPGNRPLKAL--AATGSSSMQPQSTCDKQQK-TSTRDMKSRVHVRNQNEENLKECVTSRD   257
XP_010267078.1_(NnELF3-2)        STNSDRS-TVAEDNSPLKTA--AATCNSSMQPQNACDKSQKPTRTTDMKSKQHVRNQFKENLKETVTGKN   262
XP_010267081.1_(NnELF3-3)        STYPGHS-TVATDNSPLKTA--AATCSSPMQPQNACDKNQKPTRTTDMRSK-HVRNHFEENLKETVTSIH   261
Os06t0142600_(OsELF3-1)          AANPHKS-PSTVSKSSTKCY-------------NTVSKKLERIHVSDVKSRTPLKDKEMEAAQTSKNVEV   258
Os01t0566100_(OsELF3-2)          ALSPHKS-PPAVSKSPTKCY-------------NTVSKNLERINVSDVKSRGSQKDKETGPAQTLKNVEV   254
AT2G25930.1_(AtELF3)             APSSHHSIRFQEVNQTGS------------KQNVC---------LATCSKPEVRDQVKANARS------   198
```

```
Consensus                      XXXX-------XXXXXXXXXXXXXXXXXXXXXXXXX-XXXXXX-XXXXXXXXXXXXXXXXGSXXXXXXXX
Mapoly0014s0139.1_(MpELF3)     SHFSAFRVRGADTCDQPHRRKNSQYFDDTSEADGLRSCGTGNGECLTVLEDDEDSASKHRKSSTKTRATK  415
SELMODRAFT_415241_(SmELF3)     --------------------------PRNRAPGDLG--------------------DQENGS--------  226
PO041070_(PoELF3)              -----------------LRRRIERYAP-ELESGKKT-TFSLNIPNISLVDECASKAVNNHNESL--NSCK  254
Zosma3g01990.1_(ZmELF3)        --------------------------------GNLD----------------------------------  73
XP_010244364.1_(NnELF3-1)      SAEK-------DAIHSSTGEKNAKFLNRAIASENQI-LESSQADDFRRSNNNVSRAYEEQRSILLPENCV  319
XP_010267078.1_(NnELF3-2)      HAEN-------STSISSMGERIAEPLKHDKTSGNQE-LQNGQV-GSRRLCDADAERHQENGSAMFPKICG  323
XP_010267081.1_(NnELF3-3)      RAEN-------SASVSSMGENIVEPLNNDKTSENQE-LQNSQVDDSRRLCDADEETHQKIGSTLFPEKYG  323
Os06t0142600_(OsELF3-1)        EKSSSF-----HASKDMFESRHAKVYPKMDKTG--I-INDS---DEPHGGNSGHQATSRNGGSMKFQNPP  317
Os01t0566100_(OsELF3-2)        EHFSSF-----EASKDMFGSKHAKVCPKTGTINDLD---------EPHLENSEHQATSRNGSSVKFQNPP  310
AT2G25930.1_(AtELF3)           -----------GGFVISLDVSVTEEIDLEKSASSHDRVNDYNA---SLRQESRNRLYRDGGKTRLKDTDN  254

Consensus                      XXXXXX--XXXXXXXXXXXXXXXXXXXXXXXXXXSXRXXXRXXXX-------XXXXXXXXXXXXXXXXXX
Mapoly0014s0139.1_(MpELF3)     DSEVSDSRRDQPQPASQGLGKKEKKLETEKARKPSMSTECRSNTPGSSDDNVENPSDCEVSEHTQPALWS  485
SELMODRAFT_415241_(SmELF3)     --EVRVM----------------------ASSSPSHSYESNEDNG-----RIDNQSDEELEGRCSPEEVE  267
PO041070_(PoELF3)              KLQKEIC--ES--------SKHNSPRCAEAAE---KRDDSRVGSGLGQKNLCGNHPEDINTSKTSRGDV-  310
Zosma3g01990.1_(ZmELF3)        --------------------------------GASEDAENRGQDG--------------------AGNAE  91
XP_010244364.1_(NnELF3-1)      SGDAVLV--ELVGVEKGNISREKAVPFSRASPGNSDRSPKRAEND-------RDYHEDMAHESLQVGDAD  380
XP_010267078.1_(NnELF3-2)      DADVHLV--EPNEVEKENISRERNESCSEASPVNSHRNPERAENG-------GHYHEEMVHESLQVGDVD  384
XP_010267081.1_(NnELF3-3)      NTDAHLV--ESIEVEKENISRERNEPCSEASPGNSHRNPERDENG-------GKYHEEMVHESLQVGDVD  384
Os06t0142600_(OsELF3-1)        MRRNEIS--SNPSS--ENTDRHYNLPQGGIEETGTKRKRLLEQHD-------AEKSDD-VSRLLEQHDAE  375
Os01t0566100_(OsELF3-2)        VRRNTIS--AKPSPGIENTNGHCNLPQGGLKEAGTKRKRLEAQDN-----------------------AE  355
AT2G25930.1_(AtELF3)           GAESHLA--------------------TENHSQEGHGSPEDIDND-------REYSKSRACASLQQINEE  297

Consensus                      XXDD-XSXXSXVDS-XXGXXISPDDXVGXIGXKXFWKARRAIXNQQXXFAXQVFELHRLIKVQXLIAXSP
Mapoly0014s0139.1_(MpELF3)     DSEE--SDATMLEA-DPSHKITPKDLINAVGQQEFWTARKILQRQQMIFASQVFELHCLIKVQQLLVGED  552
SELMODRAFT_415241_(SmELF3)     ASDD--SATSVVENNAAPSAITSKEIMSAVGDEEFWKMRKAMQRQQTIFQKQLFELHRLTKVQHLMANS-  334
PO041070_(PoELF3)              -GDE-ASETSILDS-IPGIDMSPDDVVGVVGPKHFWKARRAIIDQQRIFTIQVFELHRLIKVQKLIAALP  377
Zosma3g01990.1_(ZmELF3)        KHDEGMSENSILDSDLPGFNISPDDVVRAIGPKYFWKARRAIVNQQRVFALQVFELHRLIKVQKIIAGSP  161
XP_010244364.1_(NnELF3-1)      KNDD-ASETSMVDS-ISGLDISPDDVVGVIGPKHFWKARRAIVNQQKVFAVQVFELHRLIKVQRLIAGSP  448
XP_010267078.1_(NnELF3-2)      GNDD-ASESSMVDS-VLGADISPDDAVGVIGKKRFWKARRAIVNQQKLFAVQVFELHRLIEVQRLIAGSP  452
XP_010267081.1_(NnELF3-3)      RNSD-ASKCSMVDS-VLGMDISPNDVVGVIGIKHFWNARRTIVNQQKVFAAQVFELHRLIKVQRLIAGSP  452
Os06t0142600_(OsELF3-1)        NIDD-VSDSS-VEC-ITGWEISPDKIVGAIGTKHFWKARRAIMNQQRVFAVQVFELHKLVKVQKLIAASP  442
Os01t0566100_(OsELF3-2)        KIDD-LSDSS-VEC-ITAWEISPDEIVGAIGAKHFWKARRAIIK--------------------------  396
AT2G25930.1_(AtELF3)           ASDD-VSDDSMVDS-ISSIDVSPDDVVGILGQKRFWRARKAIANQQRVFAVQLFELHRLIKVQKLIAASP  365

Consensus                      XLXXXXXXXXXXXXXXXXXXXXLXXXXXXXXXXX---XXXXXXXXX-P-XXXXXXXXXXTXXXXXXXXXX
Mapoly0014s0139.1_(MpELF3)     EEDWEEDDQQATPSPPEVVPEKLQGKKTGAEKPSARPGVEDLESKG-PLQQPDSTCQSSTSKRAFYQTCT  621
SELMODRAFT_415241_(SmELF3)     -----------KISDPSKTQDEKTDKRVGSEPTH---CGEAPKTSK-PGPKPDQAPAPKTPSQT------  383
PO041070_(PoELF3)              HLLLEINPCFRSPKL---PADNLQSENTIKSQPQ---HMKQKDDSV-S-VNNIRQCPKENVKR--LLPQE  437
Zosma3g01990.1_(ZmELF3)        SLCLNKKKKNSNGNDSSKLQLHSLKKKVNGD------GIQRILPR--P-PQEEDGCNKNTPTPPPPPAY-  221
XP_010244364.1_(NnELF3-1)      HLLFDNNPYLGKPSLKVSPAKNLTSEYALKSPPG---GVKHKTDAQKP-KQDAECAAENTVGEPTLPSLS  514
XP_010267078.1_(NnELF3-2)      HLLLDGNPCLGKPPLKESPVKKLTSKYVIKSPP----CVKKKVDSQNP-NQNAEGAAENTLRKPT--PLS  515
XP_010267081.1_(NnELF3-3)      HLV-------------------------------------------------------------------  455
Os06t0142600_(OsELF3-1)        HVLIESDPCLGNALLGSK--NKLVEENLKAQP-----LLVATIDDVEPSLQQPEVSKENTEDSP-PSPHD  504
Os01t0566100_(OsELF3-2)        ----------------------------------------------------------------------  396
AT2G25930.1_(AtELF3)           DLLLDEISFLGKVSAKSYPVKKLLPSEFLVKPPLPHVVVKQRGDSEKTDQHKMESSAENVVGRLSNQGHH  435

Consensus                      XXX-----XQX-XXXXPXXXXXXXXPXXX---XXXXXXXX---PXXN--QWLXPXMXPXEGXXYKPXXGP
Mapoly0014s0139.1_(MpELF3)     PDPQPGVESGPAIPVVPSAGTSGT--------SGAWGYPPYAKSGAN--GWFGALPAVNGAYMYQPYPGP  681
SELMODRAFT_415241_(SmELF3)     ---------QP-LSTTPYAYTTHPRPIQA---ANTINYAT-SHPAYN--QWYAPLP-----FQF-PFQQP  431
PO041070_(PoELF3)              TGA-----IKV-IQNGPYSGYPPPVPVASVNRPSTWCY--H--SSVN--QWLVPVMTPSEGMTYKPFMGM  495
Zosma3g01990.1_(ZmELF3)        --------IQP-FPNGCYPGNLSMMPMMT---SG---------PTGN--QWLIPVMSPTEGLIYKPYMSP  268
XP_010244364.1_(NnELF3-1)      TGVNKGLVSQN-SSHGPHSGNPS---------SGPWCFHP---PPGN--QWLVPIMSPSEGLIYKPYVGP  569
XP_010267078.1_(NnELF3-2)      NGVNKGLVSQQ-SCHGPHSGNPPLAPMATDNKSGPWYFYP---PPGN--RWLVPIMSPSEGLVHKPVVGP  579
XP_010267081.1_(NnELF3-3)      ----------------------------------------------------------------------  455
Os06t0142600_(OsELF3-1)        TGLGSGQRDQA-ATNGVSKSNRRATPVASDNKQNNWGVQLQ--PPQN--QWLVPVMSPLEGLVYKPYSGP  569
Os01t0566100_(OsELF3-2)        ----------------------------------------------------------------------  396
AT2G25930.1_(AtELF3)           ---------QQ-SNYMPFANNPPASPA-----PNGYCFPPQPPPSGNHQQWLIPVMSPSEGLIYKPHPGM  490
```

```
Consensus                      XPX-----XXXFXXPXX------XXXXPXXXXXXX-X-------------XMXXXYXXXXXHQX-XXG
Mapoly0014s0139.1_(MpELF3)     YPQPQAHSMGAFNGSYRP-----VICAP---TPMDVSGMSTYGMPGYEQRPDLMQDMYRYNTLHQQWQAA  743
SELMODRAFT_415241_(SmELF3)     FPV----TAQVFNTPYYPAPLYGGGAAPPFLPPPDPR-------------QFMSPPIQLQPWPQH--PG  481
PO041070_(PoELF3)              YPS-----TAGFMSPIF------GGFHP--------S-------------PHLNPAYGVPTLHQQNIG  532
Zosma3g01990.1_(ZmELF3)        CLPPSTNTAAGFIPPPPP-----VGYVPHPLPIAG----------------DFMNPAYRIN------PT  310
XP_010244364.1_(NnELF3-1)      CPP-----TAGFMAPVY------GGRGPLSLPPMA-G-------------DFMNTAYGVPASHQQ-GIG  612
XP_010267078.1_(NnELF3-2)      CPL-----TAGFMAPVY------GDCGPPSLSPMA-G-------------DFMSTAYGIPASHQQ-GIG  622
XP_010267081.1_(NnELF3-3)      ----------------------------------------------------------------------  455
Os06t0142600_(OsELF3-1)        CPP-----AGSILAPFY------ANCTPLSLPSTA-G-------------DFMNSAYGVPMPHQPQHMG  613
Os01t0566100_(OsELF3-2)        ----------------------------------------------------------------------  396
AT2G25930.1_(AtELF3)           AHT-------GHYGGYY------------------G-------------HYMPTPMVMPQYH----PG  516
```

```
Consensus                      XXXXX---PXXXXXXX-PXGX--P-----------------XXXPX--XXXXXXXXXX-----------P
Mapoly0014s0139.1_(MpELF3)     MYCNSVAQDPNASWYSTPPVPYMDPSRMPMRWYDEMASSHSSAHPG--FHRQPHQSSGPSLPTDQPMVYP  811
SELMODRAFT_415241_(SmELF3)     SLYSDP---AALQW-----MGFVNP------------------PGGATYRPPAAESS----------L  513
PO041070_(PoELF3)              MHYSVP---PATSNCLPTPYGV--P---------------GADPV--ISVSAAVQPN----------P  568
Zosma3g01990.1_(ZmELF3)        AVC------PPG--FFTTPYGLVPI---------------MNNTNEVINRPTTSNIN------------  344
XP_010244364.1_(NnELF3-1)      VIPGAP---PVGQTYFP-PYGV--P---------------VLSPG--ISASAVEQRN----------P  647
XP_010267078.1_(NnELF3-2)      IFPGAL---PVGQTYFP-SYGV--P---------------VLNPG--IFASSVDQTN----------P  657
XP_010267081.1_(NnELF3-3)      ----------------------------------------------------------------------  455
Os06t0142600_(OsELF3-1)        A-PGPP---SMPMNYFP-PFSI--P---------------VMNPT--APAPVVEQGR---------HP  648
Os01t0566100_(OsELF3-2)        ----------------------------------------------------------------------  396
AT2G25930.1_(AtELF3)           M--GFP---PPGNGYFP-PYGMMPT---------------IMNPY--CSSQQQQQQQ----------P  551
```

```
Consensus                      XXXXXXXXXXXXXXXXXXX-------------------XXXXXXXX----XXXXXXXXXXXXXXXXX-XX
Mapoly0014s0139.1_(MpELF3)     MHPQWPEAVSGQVQGREGDRFRAQASWPLQGRSAEQGFQRPVTYPAQEVGAPGSQVAEGRWGNGWEASEG  881
SELMODRAFT_415241_(SmELF3)     QASSIHDDHQSR-----KD-------------------RGIH--------LRSQGSSSGFSLIQRADSS  550
PO041070_(PoELF3)              MVGSGINEKTQQCSP--IS-------------------FNIN----------------SGSAWEF-QI  598
Zosma3g01990.1_(ZmELF3)        --------TSNSCSSIKGK-------------------------KNKRSKNSNNEAHNLSST  373
XP_010244364.1_(NnELF3-1)      MAGSHPNGHSERLSTGDAN-------------------FKMHYQSSYN--NSNQKSETISCHIWKF-QT  694
XP_010267078.1_(NnELF3-2)      LVGVWPHGHAESFSSGDAY-------------------FNLHHHSSYN--NSNHNSESISCCTWKF-QM  704
XP_010267081.1_(NnELF3-3)      ----------------------------------------------------------------------  455
Os06t0142600_(OsELF3-1)        SMPQ-PYGNFEQQSWISCN-----------------MSHPSG---------------IWRF-HA  678
Os01t0566100_(OsELF3-2)        ----------------------------------------------------------------------  396
AT2G25930.1_(AtELF3)           NEQMNQFGHPGNLQNTQQQ-----------------QQRSDNEPAP----QQQQQPTKSYPRARKSRQG  599
```

```
Consensus                      SXXSXXXXX--------X-------------------XXXPXXXXXXXXXXXXXXXX----XXXXPXXX
Mapoly0014s0139.1_(MpELF3)     KSPQDSNGRAVEVPAASQYSTGEVPDSRVHKRGRETVPEARSPADRDRQRNSVEETNSGDQRRDALPLFP  951
SELMODRAFT_415241_(SmELF3)     SPAPAPSPSPAAENRRQQ-------------------HQEPRQKLVGRFCGEKQSGGESEHGVSPYSR  599
PO041070_(PoELF3)              SKDSELQGS--------R-------------------ASTPGEKVQ--------EV----RDTLPLFL  627
Zosma3g01990.1_(ZmELF3)        SSTSE-----------------------------KTPPPESEERSG-----------NGLLSFKS  398
XP_010244364.1_(NnELF3-1)      SKDSELQGS--------T-------------------ASSPCERSQGSGSGNVAEG----RDALPLFP  731
XP_010267078.1_(NnELF3-2)      SKDSEPQGS--------T-------------------ASSPCERTRSTGANNIAEG----RDPLPLFP  741
XP_010267081.1_(NnELF3-3)      ----------------------------------------------------------------------  455
Os06t0142600_(OsELF3-1)        SRDSEAQASS-----------------------ASSPFDRFQCSG-----------SGPVSAFP  708
Os01t0566100_(OsELF3-2)        ----------------------------------------------------------------------  396
AT2G25930.1_(AtELF3)           STGSSPSGP--------Q-------------------GISGSKSFRPFAAVDEDSN----INNAPEQT  636
```

```
Consensus                      XXXXXXXXXXXXXS---XXXXXXXXVIKVVPHNXXXAXESAARIFXSIQXERXXXX----
Mapoly0014s0139.1_(MpELF3)     LATSANAGEKQKSSSHSFPG----VIKVVPRAMVATAESAAEILLSIQKQRQQ------  1000
SELMODRAFT_415241_(SmELF3)     PSGFQSVKNSSSSSKEQRSSQGSHKAIKVTPRAAPVTAESAAEILHSIQKERPS------  653
PO041070_(PoELF3)              TATFTEVSDEHMQT---HCSDHRTQVIKVVSHNPVSATESAVRIFPSIQKERLQHYST--  682
Zosma3g01990.1_(ZmELF3)        MASTVEEDTEHTRSSTHHSDRQQAQVIKVVPHNKRSATESAARIFQSIQEERQLYE----  454
XP_010244364.1_(NnELF3-1)      MAPAVQVQDQVPKS---HSSGQQTRVIKVVPHNRRSATESAVRIFQSIQKERQQYDSV--  786
XP_010267078.1_(NnELF3-2)      MAPAVQVQNPVLQS---RSSDQQTRVIRVVPHNRRSATESAARIFQSIQKERQKYDSV--  796
XP_010267081.1_(NnELF3-3)      ----------------------------------AFHSNMEQPRKYL----  468
Os06t0142600_(OsELF3-1)        TVSAQNNQPQPSYS----SRDNQTNVIKVVPHNSRTASESAARIFRSIQMERQRDD----  760
Os01t0566100_(OsELF3-2)        ------------------------------------------------------------  396
AT2G25930.1_(AtELF3)           MTTTTTT-TRTTVTQTTRDGGGVTRVIKVVPHNAKLASENAARIFQSIQEERKRYDSSKP  695
```

Consensus Threshold: >50%

Compare to: the consensus

Amino acids that match the reference are marked with yellow highlighting.

Created: 12 Apr 2023

Last Modified: 12 Apr 2023
