# Supplementary material for: Dim artificial light at night alters gene expression rhythms and growth in a key seagrass species (Posidonia oceanica)
Source: Sci Rep. 2023 Jun 30;13:10620. doi: 10.1038/s41598-023-37261-3 (PMC10313690; doi:10.1038/s41598-023-37261-3)
Supplement: Supplementary file 4 — Supplementary Information 4. [file 41598_2023_37261_MOESM4_ESM.pdf]

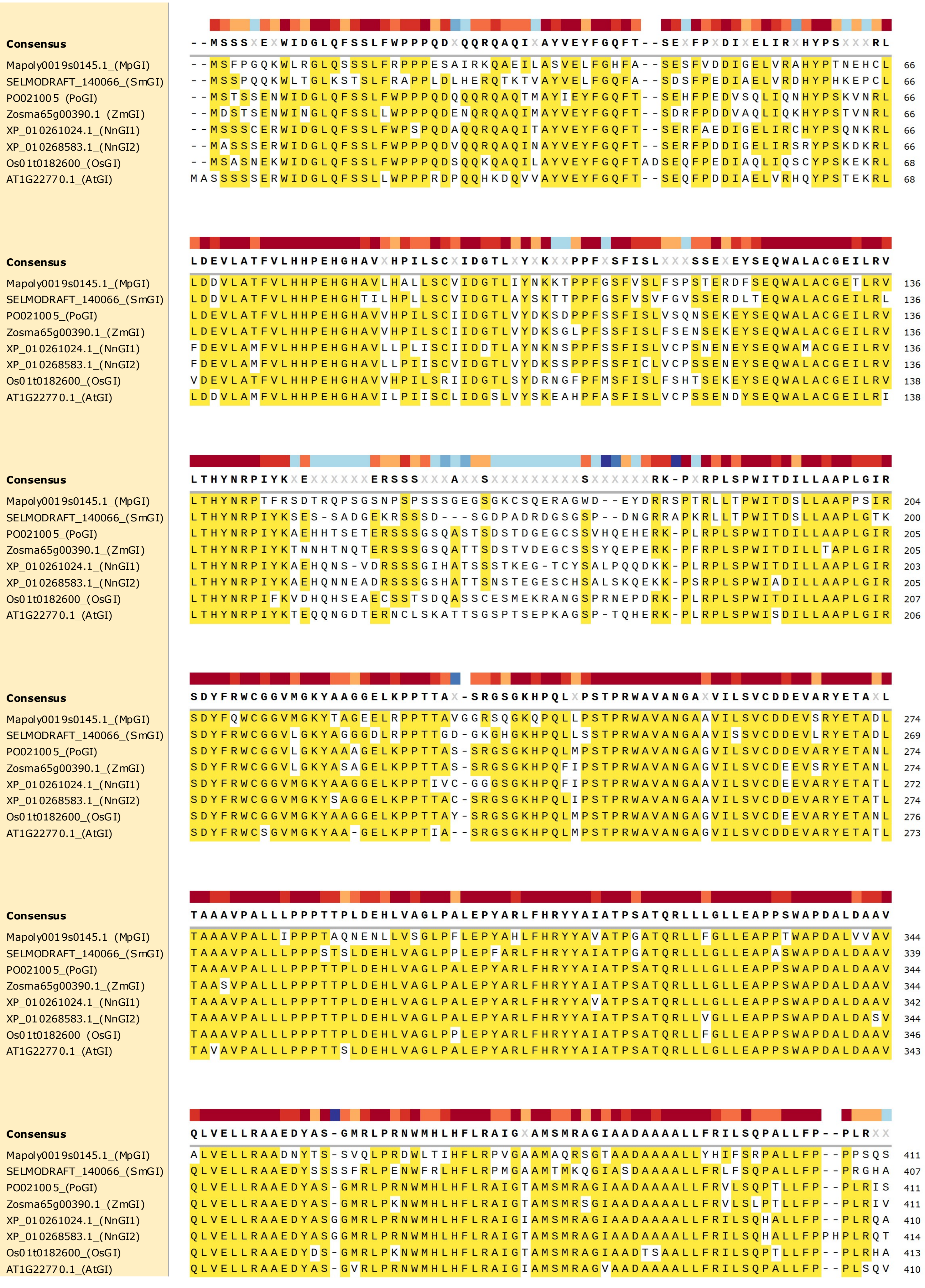

Consensus --MSSSXEXWIDGLQFSSLFWPPPQDXQQRQAQIXAYVEYFGQFT--SEXFPXDIXELIRXHYPSXXXRL
Mapoly0019s0145.1_(MpGI) --MSFPGQKWLRGLQSSSLFRPPPESAIRKQAEILASVELFGHFA--SESFVDDIGELVRAHYPTNEHCL 66
SELMODRAFT_140066_(SmGI) --MSSPQQKWLTGLKSTSLFRAPPLDLHERQTKTVAYVELFGQFA--SDSFPEDIAELVRDHYPHKEPCL 66
PO021005_(PoGI) --MSTSSENWIDGLQFSSLFWPPPQDQQQRQAQTMAYIEYFGQFT--SEHFPEDVSQLIQNHYPSKVNRL 66
Zosma65g00390.1_(ZmGI) --MDSTSENWINGLQFSSLLWPPPQDENQRQAQIMAYVEYFGQFT--SDRFPDDVAQLIQKHYPSTVNRL 66
XP_010261024.1_(NnGI1) --MSSSCERWIDGLQFSSLFWPSPQDAQQRQAQITAYVEYFGQFT--SERFAEDIGELIRCHYPSQNKRL 66
XP_010268583.1_(NnGI2) --MASSSERWIDGLQFSSLFWPPPQDVQQRQAQINAYVEYFGQFT--SERFPDDIGELIRSRYPSKDKRL 66
Os01t0182600_(OsGI) --MSASNEKWIDGLQFSSLFWPPPQDSQQKQAQILAYVEYFGQFTADSEQFPEDIAQLIQSCYPSKEKRL 68
AT1G22770.1_(AtGI) MASSSSSERWIDGLQFSSLLWPPPRDPQQHKDQVVAYVEYFGQFT--SEQFPDDIAELVRHQYPSTEKRL 68
Consensus LDEVLATFVLHHPEHGHAVXHPILSCXIDGTLXYXKXXPPFXSFISLXXXSSEXEYSEQWALACGEILRV
Mapoly0019s0145.1_(MpGI) LDDVLATFVLHHPEHGHAVLHALLSCVIDGTLIYNKKTPPFGSFVSLFSPSTERDFSEQWALACGETLRV 136
SELMODRAFT_140066_(SmGI) LDDVLATFVLHHPEHGHTILHPLLSCVIDGTLAYSKTTPPFGSFVSVFGVSSERDLTEQWALACGEILRL 136
PO021005_(PoGI) LDEVLATFVLHHPEHGHAVVHPILSCIIDGTLVYDKSDPPFSSFISLVSQNSEKEYSEQWALACGEILRV 136
Zosma65g00390.1_(ZmGI) LDEVLATFVLHHPEHGHAVVHPILSCIIDGTLVYDKSGLPFSSFISLFSENSEKEYSEQWALACGEILRV 136
XP_010261024.1_(NnGI1) FDEVLAMFVLHHPEHGHAVLLPLISCIIDDTLAYNKNSPPFSSFISLVCPSNENEYSEQWAMACGEILRV 136
XP_010268583.1_(NnGI2) FDEVLAMFVLHHPEHGHAVLLPIISCVIDGTLVYDKSSPPFSSFICLVCPSSENEYSEQWALACGEILRV 136
Os01t0182600_(OsGI) VDEVLATFVLHHPEHGHAVVHPILSRIIDGTLSYDRNGFPFMSFISLFSHTSEKEYSEQWALACGEILRV 138
AT1G22770.1_(AtGI) LDDVLAMFVLHHPEHGHAVILPIISCLIDGSLVYSKEAHPFASFISLVCPSSENDYSEQWALACGEILRI 138
Consensus LTHYNRPIYKXEXXXXXXERSSSXXXAXXSXXXXXXXXXSXXXXXXRK-PXRPLSPWITDILLAAPLGIR
Mapoly0019s0145.1_(MpGI) LTHYNRPTFRSDTRQPSGSNPSPSSSGEGSGKCSQERAGWD--EYDRRSPTRLLTPWITDSLLAAPPSIR 204
SELMODRAFT_140066_(SmGI) LTHYNRPIYKSES-SADGEKRSSSD---SGDPADRDGSGSP--DNGRRAPKRLLTPWITDSLLAAPLGTK 200
PO021005_(PoGI) LTHYNRPIYKAEHHTSETERSSSGSQASTSDSTDGEGCSSVHQEHERK-PLRPLSPWITDILLAAPLGIR 205
Zosma65g00390.1_(ZmGI) LTHYNRPIYKTNNHTNQTERSSSGSQATTSDSTVDEGCSSSYQEPERK-PFRPLSPWITDILLTAPLGIR 205
XP_010261024.1_(NnGI1) LTHYNRPIYKAEHQNS-VDRSSSGIHATSSSTKEG-TCYSALPQQDKK-PLRPLSPWITDILLAAPLGIR 203
XP_010268583.1_(NnGI2) LTHYNRPIYKAEHQNNEADRSSSGSHATTSNSTEGESCHSALSKQEKK-PSRPLSPWIADILLAAPLGIR 205
Os01t0182600_(OsGI) LTHYNRPIFKVDHQHSEAECSSTSDQASSCESMEKRANGSPRNEPDRK-PLRPLSPWITDILLAAPLGIR 207
AT1G22770.1_(AtGI) LTHYNRPIYKTEQQNGDTERNCLSKATTSGSPTSEPKAGSP-TQHERK-PLRPLSPWISDILLAAPLGIR 206
Consensus SDYFRWCGGVMGKYAAGGELKPPTTAX-SRGSGKHPQLXPSTPRWAVANGAXVILSVCDDEVARYETAXL
Mapoly0019s0145.1_(MpGI) SDYFQWCGGVMGKYTAGEELRPPTTAVGGRSQGKQPQLLPSTPRWAVANGAAVILSVCDDEVSRYETADL 274
SELMODRAFT_140066_(SmGI) SDYFRWCGGVLGKYAGGGDLRPPTTGD-GKGHGKHPQLLSSTPRWAVANGAAVISSVCDDEVLRYETADL 269
PO021005_(PoGI) SDYFRWCGGVLGKYAAAGELKPPTTAS-SRGSGKHPQLMPSTPRWAVANGAGVILSVCDDEVARYETANL 274
Zosma65g00390.1_(ZmGI) SDYFRWCGGVLGKYASAGELKPPTTAS-SRGSGKHPQFIPSTPRWAVANGAGVILSVCDEEVSRYETANL 274
XP_010261024.1_(NnGI1) SDYFRWCGGVMGKYAAGGELKPPTIVC-GGGSGKHPQFIPSTPRWAVANGAAVILSVCDEEVARYETATL 272
XP_010268583.1_(NnGI2) SDYFRWCGGVMGKYSAGGELKPPTTAC-SRGSGKHPQLIPSTPRWAVANGAAVILSVCDDEVARYETATL 274
Os01t0182600_(OsGI) SDYFRWCGGVMGKYAAGGELKPPTTAY-SRGSGKHPQLMPSTPRWAVANGAGVILSVCDEEVARYETANL 276
AT1G22770.1_(AtGI) SDYFRWCSGVMGKYAA-GELKPPTIA--SRGSGKHPQLMPSTPRWAVANGAGVILSVCDDEVARYETATL 273
Consensus TAAAVPALLLPPPTTPLDEHLVAGLPALEPYARLFHRYYAIATPSATQRLLLGLLEAPPSWAPDALDAAV
Mapoly0019s0145.1_(MpGI) TAAAVPALLIPPPTAQNENLLVSGLPFLEPYAHLFHRYYAVATPGATQRLLFGLLEAPPTWAPDALVVAV 344
SELMODRAFT_140066_(SmGI) TAAAVPALLLPPPSTSLDEHLVAGLPPLEPFARLFHRYYAIATPGATQRLLLGLLEAPASWAPDALDAAV 339
PO021005_(PoGI) TAAAVPALLLPPPTTPLDEHLVAGLPALEPYARLFHRYYAIATPSATQRLLLGLLEAPPSWAPDALDAAV 344
Zosma65g00390.1_(ZmGI) TAASVPALLLPPPTTPLDEHLVAGLPALEPYARLFHRYYAIATPSATQRLLLGLLEAPPSWAPDALDAAV 344
XP_010261024.1_(NnGI1) TAAAVPALLLPPPTTPLDEHLVAGLPALEPYARLFHRYYAVATPSATQRLLLGLLEAPPSWAPDALDAAV 342
XP_010268583.1_(NnGI2) TAAAVPALLLPPPTTPLDEHLVAGLPALEPYARLFHRYYAIATPSATQRLLVGLLEAPPSWAPDALDASV 344
Os01t0182600_(OsGI) TAAAVPALLLPPPTTPLDEHLVAGLPPLEPYARLFHRYYAIATPSATQRLLFGLLEAPPSWAPDALDAAV 346
AT1G22770.1_(AtGI) TAVAVPALLLPPPTTSLDEHLVAGLPALEPYARLFHRYYAIATPSATQRLLLGLLEAPPSWAPDALDAAV 343
Consensus QLVELLRAAEDYAS-GMRLPRNWMHLHFLRAIGXAMSMRAGIAADAAAALLFRILSQPALLFP--PLRXX
Mapoly0019s0145.1_(MpGI) ALVELLRAADNYTS-SVQLPRDWLTIHFLRPVGAAMAQRSGTAADAAAALLYHIFSRPALLFP--PPSQS 411
SELMODRAFT_140066_(SmGI) QLVELLRAAEDYSSSSFRLPENWFRLHFLRPMGAAMTMKQGIASDAAAALLFRLFSQPALLFP--PRGHA 407
PO021005_(PoGI) QLVELLRAAEDYAS-GMRLPRNWMHLHFLRAIGTAMSMRAGIAADAAAALLFRVLSQPTLLFP--PLRIS 411
Zosma65g00390.1_(ZmGI) QLVELLRAAEDYAS-GMRLPKNWMHLHFLRAIGTAMSMRSGIAADAAAALLFRVLSLPTLLFP--PLRIV 411
XP_010261024.1_(NnGI1) QLVELLRAAEDYASGGMRLPRNWMHLHFLRAIGIAMSMRAGIAADAAAALLFRILSQHALLFP--PLRQA 410
XP_010268583.1_(NnGI2) QLVELLRAAEDYASGGMRLPRNWMHLHFLRAIGTAMSMRAGIAADAAAALLFRILSQHALLFPPHPLRQT 414
Os01t0182600_(OsGI) QLVELLRAAEDYDS-GMRLPKNWMHLHFLRAIGTAMSMRAGIAADTSAALLFRILSQPTLLFP--PLRHA 413
AT1G22770.1_(AtGI) QLVELLRAAEDYAS-GVRLPRNWMHLHFLRAIGIAMSMRAGVAADAAAALLFRILSQPALLFP--PLSQV 410

```
Consensus                    EGXEXXHXPLGXYXSX------XXKQXEXPAAEATIEATAQGIASMLCAHGPEVEWRICTIWEAAYGLXP
Mapoly0019s0145.1_(MpGI)     QGLFSTQTLL--YGTS--SLAASREEAKAAAQESEVATASGLAALLTGHGIDVECHICAIWEAAYGLRS   477
SELMODRAFT_140066_(SmGI)     QGAQVVQ-PL--YGPPIRIDVLFHAQMEALATQVNEEATAKGVASLMRDHGRDVEWRICVLWEAAYGLIP   474
PO021005_(PoGI)              EGAEIQHGSVGGCVSP------SNKQSEAPCAEATVEATAQGIASMLCAHGPEVEWRICTIWEAAYGLLP   475
Zosma65g00390.1_(ZmGI)       DGDDDKQEST--CSPS--------KQIDATRAEENIEATAQGIASMLCAHGPEVEWRICTIWEAAYGLLP   471
XP_010261024.1_(NnGI1)       EGVEVQHEPLGGYISC------HRKQIEVPGAEATIEATAQGIASMFCAHGPEVEWRICTIWEVAYGLIP   474
XP_010268583.1_(NnGI2)       EGVEVQHEPLGSYISC------YGKQIEVPAAEATIEATAQGIASMFCAHGPEVEWRICTIWEAAYGLIP   478
Os01t0182600_(OsGI)          EGVELHHEPLGGYVSS------YKRQLEVPASEATIDATAQGIASMLCAHGPDVEWRICTIWEAAYGLLP   477
AT1G22770.1_(AtGI)           EGVEIQHAPIGGYSSN------YRKQIEVPAAEATIEATAQGIASMLCAHGPEVEWRICTIWEAAYGLIP   474
```

```
Consensus                    LSSSAVDLPEIVVATPLQPPXLSWNLYXPLLKVLEYLPRGSPSEACLMRIFVATVEAILRRTFPXXXSXE
Mapoly0019s0145.1_(MpGI)     LTPSSVDLPDLVLSTPLQPPVLSWNLLRALFRILSYLPPESPSQACLKRIFSATIEAILQRTFPLD-EVK   546
SELMODRAFT_140066_(SmGI)     LDKSVVDLPEMVIATPLQPPLLSWTLFRPFLRVLEHVPKGCQSQTCLRRIFSATVDAILRRTFPLD-DWK   543
PO021005_(PoGI)              LSSSAVDLPEIVVATPLQPPALSWNLYLPLLKVLEYLPRGSPSEACLMRIFVATVEAILQRTFPPESSLE   545
Zosma65g00390.1_(ZmGI)       LSSSAVDLPEIVVATPLQPPSLSWSLYRPLLKVLEYLPRGSPSEACLMRIFVATVEAILRRTFPLD-SLP   540
XP_010261024.1_(NnGI1)       LSSSAVDLPEIIVATPLQPPVLSWNLYLPLLKVLEYLPRGSPSEACLMRIFVATVEAILRRTFPPKSSRE   544
XP_010268583.1_(NnGI2)       LSSSAVDLPEIIVATPVQPPVLSWNLYLPLLKVLEYLPRGSPSEACLMRIFVATVEAILRRTFPPESSRE   548
Os01t0182600_(OsGI)          LSSSAVDLPEIVVAAPLQPPTLSWSLYLPLLKVFEYLPRGSPSEACLMRIFVATVEAILRRTFPSE-TSE   546
AT1G22770.1_(AtGI)           LNSSAVDLPEIIVATPLQPPILSWNLYIPLLKVLEYLPRGSPSEACLMKIFVATVETILSRTFPPESSRE   544
```

```
Consensus                    QXRKXXX----XXXXXSXXKNLAXAELRTMVHSLFLESXASXDLASRLLFVVLTVCVSHEAXPX-GSKRX
Mapoly0019s0145.1_(MpGI)     QQKDGTIVGLQAGGGGAGAKSVGMGELRAMLHCLFTESFLSPELAAHLLSEALSLCLSHDASRQLERRKA   616
SELMODRAFT_140066_(SmGI)     EQKNGNF----RSASGSGVDPAGMAELRALVHCLFTEAFLGPALASQLLSDALTVCLSHDTLRQ-GN---   605
PO021005_(PoGI)              QTRKQKT----HGGVWSTTKNLAAAELRTMVHSLFLESCASMDLASRLLFVVLTVCVSHEALPN-GSKRT   610
Zosma65g00390.1_(ZmGI)       DQRKKI------QGSWS-------TELRTMIHSLFLESSVSTDLASRLIFVVLTVCVSYEVLPN-GSKRT   597
XP_010261024.1_(NnGI1)       QIRKSRF----LLNIGSASKNLAVAELRTMVHSLFLESRASIDLASRLLFVVLTVCVSHEAQPN-GSKRP   609
XP_010268583.1_(NnGI2)       QIRKSRF----LFDYGSASKNLAVAELRTMVHSLFLESCASIDLASRLLFVVLTVCVNHEAQPS-GSKRP   613
Os01t0182600_(OsGI)          QSRKPRS----------QSKNLAVAELRTMIHSLFVESCASMDLASRLLFVVLTVCVSHQALPG-GSKRP   605
AT1G22770.1_(AtGI)           LTRKARS----SFTTRSATKNLAMSELRAMVHALFLESCAGVELASRLLFVVLTVCVSHEAQSS-GSKRP   609
```

```
Consensus                    XGXDXX-XSXXXXEXXXXXNXXXXXXXXRXX----KXQGPVAXFDSYVLAAVCALACELQLXPX------
Mapoly0019s0145.1_(MpGI)     TGKDGG-SSLTMDED---IYGGKEIFRTSTIDVSGKQRGAVATFDSYVIAAVCALACEVQFFPFSPAYFT   682
SELMODRAFT_140066_(SmGI)     -GSDS--------------------SKKRSTHSSNKDRGAVASFDSYLIAAVCALACEVQLCTF------   648
PO021005_(PoGI)              TGDIN--SSDDMTEELHGLNGKQT-IKKRTR----KKQGPVAAFDSYVLAAVCALACELQLFPL------   667
Zosma65g00390.1_(ZmGI)       TGDAARKSSDDIPEE---VNGGTS-VKIRSR----KKQGPVTVFDSFVLAAVCALSCELQLFPL------   653
XP_010261024.1_(NnGI1)       RCDDIY-PTNEVTEDSQVINEKNGEVRTRKV----KRQGPVAAFDSYVLAAICALACELQLCPL------   668
XP_010268583.1_(NnGI2)       RSDDIY-PSNEVTEDFQVTNEKNGEVRTRKV----KRQGPVAAFDSYVLAAVCALACELQLYPL------   672
Os01t0182600_(OsGI)          TGSDNH-SSEEVTNDSRLTNG-----RNRCK----KRQGPVATFDSYVLAAVCALSCELQLFPF------   659
AT1G22770.1_(AtGI)           RSEYAS-TTENIEANQPVSNNQTANRKSRNV----KGQGPVAAFDSYVLAAVCALACEVQLYPM------   668
```

```
Consensus                    IXKXGX--XSNXXXXXXIXKXXXXNG------XSXXXXXGXXXAIXHTRRILXILEALFSLKPSSVGTSW
Mapoly0019s0145.1_(MpGI)     IMKPPSVVMTRPDADGQVSSSGARDGQRSLSLLSNRFPIGVANVLEHTKRLLGLLELLLGVSPSFAGTGS   752
SELMODRAFT_140066_(SmGI)     SAADGT--AFN----------------------------GVTNSAYQARRLMSVLEGLLVVEPFSPGVGP   688
PO021005_(PoGI)              IMKNGN--CSNSKDLVRISKAANENG------VSDDFDSGVGSAVCHTRRILGILEALFSLKPSSVGTSW   729
Zosma65g00390.1_(ZmGI)       IMKKG-----------RISKPKNSN------------ENSVGSAISHTRRILRILEAHFSLKPSSIGTSW   700
XP_010261024.1_(NnGI1)       ILKSDK--RSNFKDASSTPKHEKVNG------SSNDLRNGMGAAISHTHRILRILEALFSLKPSSVGTSW   730
XP_010268583.1_(NnGI2)       LSKTGK--CSDCKDAPIIVKPEKVNG------SSNDLWNGMGAAINHTRRILRILEALFSLKPSSVGTSW   734
Os01t0182600_(OsGI)          ISKNGN--HSNLKDSIKIVIPGKTTG------ISNELHNSISSAILHTRRILGILEALFSLKPSSVGTSW   721
AT1G22770.1_(AtGI)           ISGGGN--FSNSAVAGTITKPVKING------SSKEYGAGIDSAISHTRRILAILEALFSLKPSSVGTPW   730
```

```
Consensus                    SY---SSNEIVAAAMVAAHVSELFRRSKACXHALSXLMRCKWDXEIXXRASSLYXLIDXHGKXVASIVXK
Mapoly0019s0145.1_(MpGI)     SSSRTSTNEILGQAVGAAHMSDLLGHSRACLHSLTGIMRCKWDPGICLKASAVLSAIESNGDLVAIATHG   822
SELMODRAFT_140066_(SmGI)     NTN--SPNDLVEAAIVAAHISRLLGRSRACTHALTAIVRCKWDPGVSSKAASILALVDGNDKAVEAVFNY   756
PO021005_(PoGI)              SY---SSNEIVAAAMVAAHVSELFRRSKACMNALSVLMRCKWDTEICTRASSLYHLIDFHGKTVASIVDK   796
Zosma65g00390.1_(ZmGI)       GC---SSNEIVAAAMVAAHVSELFRQSKACMNSLTILMRCKWDPEISNRSSSLYHLIDFHGKAVASIVDK   767
XP_010261024.1_(NnGI1)       SY---SSNEIVAAAMVAAHVSELFRRSKVCMHALSILMQCKWDNEIYTRASSLYNLIDIHGKAVASIAVK   797
XP_010268583.1_(NnGI2)       SY---SSNEIVAAAMVAAHVSELFRRSKPCTHALSLLMRCKWDNEICNRASSLYNLIDIHGKAVASIVIK   801
Os01t0182600_(OsGI)          SY---SSNEIVAAAMVAAHVSELFRRSRPCLNALSALKQCKWDAEISTRASSLYHLIDLHGKTVTSIVNK   788
AT1G22770.1_(AtGI)           SY---SSSEIVAAAMVAAHISELFRRSKALTHALSGLMRCKWDKEIHKRASSLYNLIDVHSKVVASIVDK   797
```

```
Consensus                      AEPLEAXLXXXPXXK---DXXXXXXXXXXXXXXXXXXXXXXXXSXXXXXXXXXXXXXX-XXXXXXXXXXN
Mapoly0019s0145.1_(MpGI)       RNGFGGPDCTIGIKR---DLEQAWNTSIEDQFKPSQLQKLSRVSGLPGNLRGGSSEKG-------PNIPN  882
SELMODRAFT_140066_(SmGI)       ADKLSGDE-----KR---------------------TLSSKQAQKIISGFTKDGVKD-------------  787
PO021005_(PoGI)                AEPLEAHLVHVPLWK---DASVYLNGSK----RQLQGFQKEEIPALHSNDRNGSASSGTLSKSEKALLLN  859
Zosma65g00390.1_(ZmGI)         AEPLEAHLVQVPLWKVTSDNRGRNSNKRHIDSTNFGGLEKKQNSALQNGRRDGSCNSG-MCRDKTELLLN  836
XP_010261024.1_(NnGI1)         AEPLAAHLVQAPLWK---DSSVCNTGRKQVNYSKTNCFKSESSSTSWPEGDNDSTHSKSFLKCEKASLSN  864
XP_010268583.1_(NnGI2)         AEPLEANLVQAPVWK---DASVCTNGRKKVNCRNTNYHDSGSSSTKQCESEKDPIHSNNFLKCEKESLSN  868
Os01t0182600_(OsGI)            AEPLEAHLTLTPVKK---DEPPI--EEKNINSSDGGALEKKDAS--RSHRKNGFARP--LLKCAEDVILN  849
AT1G22770.1_(AtGI)             AEPLEAYLKNTPVQK---DSVTCLNWKQENTCASTTCFDTAVTSASRTEMNPRGNHK--YARH-----SD  857

Consensus                      XXXXXXXXKXIASXXXDASDLANFLTXDRXXGXNCSXQXLLRSVLXXKXELCFSVVSLLWHKLIAXPEXQ
Mapoly0019s0145.1_(MpGI)       G--YGPVGEARGSFPLEASDVASLLCKDRRNGRNGTLDTSLKSVLVGKQHLAIGATALLCQRLILAPDIP  950
SELMODRAFT_140066_(SmGI)       -----------ASLSLNASDVTNLLC--GCNGVSTTVSDLLKAVLKQKRDLAVAIVPLLWQRLMSAEELP  844
PO021005_(PoGI)                DIMLNRSEKNIASLPVDASDLANFLTMDRHIGFNCSAQALLRSVLHDKNELCFSVVSLLWHKLIAAPETQ  929
Zosma65g00390.1_(ZmGI)         S---SASKKNIARLAEDASDLANFLIRDRHIGLNCSAQAFLRSVLQDRNKLCFSVVSLLWHKLIATPETE  903
XP_010261024.1_(NnGI1)         DRIVNTMAKSIENFSMDASDLAHFLTMDRHIGLNCSAKVLLRSVLAEKQELCFSVVSLLWHKLIASPETQ  934
XP_010268583.1_(NnGI2)         DGMIDTMGKSIASFSKDALDLANFLTMDRHIGFNCSAQVLLRSVLAEKQELCFSVVSLLWHKLIATPETQ  938
Os01t0182600_(OsGI)            GDVASTSGKAIASLQVEASDLANFLTMDRNGGYRGS-QTLLRSVLSEKQELCFSVVSLLWQKLIASPEMQ  918
AT1G22770.1_(AtGI)             EGSGRPSEKGIKDFLLDASDLANFLTADRLAGFYCGTQKLLRSVLAEKPELSFSVVSLLWHKLIAAPEIQ  927

Consensus                      XXAESTSAXQGWRQVVDALCNVVXAXPXKAXXAIVLQAERXLQPWIARDDEQGQK-MWRINQRIVKLIXE
Mapoly0019s0145.1_(MpGI)       NAIEGTSAERGWGQVVEALCNISAAFPGKVARFVVSQAEAELRPWTIKDDGPSQQSLWRINTRVVCLLSD  1020
SELMODRAFT_140066_(SmGI)       TSKEGTSAKQGWRQVVDAVCNVVLTYPEKATSVVLLQAERGIQPWIIGDG--GEE-KWRMNTRIVFLLSE  911
PO021005_(PoGI)                MTAESTSAQQGWRQVVDALCNVVLALPAKASAAIVLQAERDLQPWIARDDEQGQK-MWTINQRIVKLIVE  998
Zosma65g00390.1_(ZmGI)         MCAESTSAQQGWSQVVDALCNVVAAFPTKATAAIVLQAERDLQPWIARDDEQGQK-IWMVNQRIVKLIAE  972
XP_010261024.1_(NnGI1)         PSAESTSAHQGWRQVIDALCNVVSASPAKASTAIVLQAERELQPWIARDDEQGQK-MWRINQRIVKLIAE  1003
XP_010268583.1_(NnGI2)         PSAESTSAHQGWRQVVDALCNVVSASPAKASTAIVLQAERELQPWIARDDEQGQK-MWRINQRIVKLIVE  1007
Os01t0182600_(OsGI)            MSAESTSAHQGWRKVVDALCDIVSASPTKASAAIVLQAEKDLQPWIARDDEQGQK-MWRVNQRIVKLIAE  987
AT1G22770.1_(AtGI)             PTAESTSAQQGWRQVVDALCNVVSATPAKAAAAVVLQAERELQPWIAKDDEEGQK-MWKINQRIVKVLVE  996

Consensus                      LMRNHDXPEXLVILASASDLLLRATDGMLVDGEACTLPQLELLEXXARAXXXXXXWGXSGXXVADGLSNL
Mapoly0019s0145.1_(MpGI)       LLRLTHLPEIVKVVVHDGALLRRATDGMTLDGEACKLPQLELLEAAALAAQAALKWEEPNEEVADQLFSL  1090
SELMODRAFT_140066_(SmGI)       LLRLND-PQVLGLIANAGTLLYQATDGMSVDGEPCTLPQLELLEAIAMAIKSLCAWKVS----SRGLLIL  976
PO021005_(PoGI)                LMRNHESPEALVILASASDLLLRATDGMLVDGEACTLPQLELLEVTARAVQLVLDWGVSGLAVADGLSNL  1068
Zosma65g00390.1_(ZmGI)         LMRNHDTPEALVILSSASDLLLRATDGMLVDGEACTLPQLELLEVAASGIKLVLEWGQYGFAVADGLSNL  1042
XP_010261024.1_(NnGI1)         LMRNHDSPESLVILASASDLLLRATDGMLVDGEACTLPQLELLEATARAVQLVVEWGESGSAVADGLSNL  1073
XP_010268583.1_(NnGI2)         LIRNHDNPESLVILASASDLLLRATDGMLVDGEACTLPQLEVISQENR----------------------  1055
Os01t0182600_(OsGI)            LMRNHDSPEALVILASASDLLLRATDGMLVDGEACTLPQLELLEVTARAVHLIVEWGDSGVSVADGLSNL  1057
AT1G22770.1_(AtGI)             LMRNHDRPESLVILASASDLLLRATDGMLVDGEACTLPQLELLEATARAIQPVLAWGPSGLAVVDGLSNL  1066

Consensus                      LKCRLPATXRCLSHPSAHVRALSXSVLRDILXXX--XXXXXXXXXXXXXXXXXXXXXXXXXXXXIXWXXX
Mapoly0019s0145.1_(MpGI)       LRERLPATVRCLSHNSTHVRAMSVARLRDMLYME--SLRSSSSRKSSEGSEREEAN------GTGNWRRY  1152
SELMODRAFT_140066_(SmGI)       LKERLPAIVRCLSHDSPRIRASSASLLREIVSTD--VLRAS--YPGDKAGA---------------WLED  1027
PO021005_(PoGI)                LKCRLPATIRCLSHPSAHVRALSTSVLRDILHSR--PIKPN-NLKQDDMKSICEPSYQCLNVGIIDWHEN  1135
Zosma65g00390.1_(ZmGI)         LKCRLPATIQCLSHPSAHVRALSTSVLRDIM-----PIQLGIKFKKEAIKN------------SIDWQEN  1095
XP_010261024.1_(NnGI1)         LKCRLPATVHCLSRPSAHVRALSVSVLRDVLYIG--SNRSN--SKQEGIQAIHGTRYPYLSLGIIDWHED  1139
XP_010268583.1_(NnGI2)         --CY------CFCYE-----------FKALLHMW--------------------------------WLLG  1074
Os01t0182600_(OsGI)            LKCRLSTTIRCLSHPSAHVRALSMSVLRDILNSG--QINSSKLIQGEHRNGIQSPTYQCLAASIINWQAD  1125
AT1G22770.1_(AtGI)             LKCRLPATIRCLSHPSAHVRALSTSVLRDIMNQSSIPIKVTPKLPTTEKNGMNSPSYRFFNAASIDWKAD  1136

Consensus                      XEXCXXWEAXSRXAXGXXXXFLXXAAXXLGCXXXXX-------
Mapoly0019s0145.1_(MpGI)       VEQCVVWEVHCRRAGGMSISLLANAASALGCPIPV--------  1187
SELMODRAFT_140066_(SmGI)       VEQSIAWETHYRRAEGLSESFLASAAIALGCKLPPS-------  1063
PO021005_(PoGI)                IEKCLKWEARSRIATGLTLAFLTTAAKDLSVPLPSE-FYSAAS  1177
Zosma65g00390.1_(ZmGI)         IDGCFKWEAKSRIETGLTLVFLTAAAKELNLPLPTESIYAAES  1138
XP_010261024.1_(NnGI1)         IEKCLKCEAQSRHASGMNITFLGAAAKELGCTIST--------  1174
XP_010268583.1_(NnGI2)         ANDCIFY-------------F---------C------------  1083
Os01t0182600_(OsGI)            VERCIEWEAHSRRATGLTLAFLTAAAKELGCPLTC--------  1160
AT1G22770.1_(AtGI)             IQNCLNWEAHSLLSTTMPTQFLDTAARELGCTISLSQ------  1173
```

Consensus Threshold: >50%

Compare to: the consensus

Amino acids that match the reference are marked with yellow highlighting.

Created: 12 Apr 2023

Last Modified: 12 Apr 2023
