# Supplementary material for: Dim artificial light at night alters gene expression rhythms and growth in a key seagrass species (Posidonia oceanica)
Source: Sci Rep. 2023 Jun 30;13:10620. doi: 10.1038/s41598-023-37261-3 (PMC10313690; doi:10.1038/s41598-023-37261-3)
Supplement: Supplementary file 5 — Supplementary Information 5. [file 41598_2023_37261_MOESM5_ESM.pdf]

```
Consensus                              MXXXXXXXXXXXX---XDXXRVXEWEXGLPXAD--DLTPLSQXLIPPXLAXAFSITPEPX-XXXXDVXXA
Mapoly0101s0068.1_(MpLUX)              MAAMK-----------DDNNRVREWEAGLPSAS--DLTPLHHSLITRVLARAFCIPTNATPPSAADVLQA   57
XP_024525426.1_(...without_first_aa    MAAV------------KENDRVEEWEEGLPTPE--ELTPLNQSLITPELASAFSISQEAA-KSSSDVLHA   56
PO025399_(PoLUX)                       MADQQPHP--------EDSSRVVEWTEGLPTADDGDLTPLSQPLITPELASAFSVTPEPN-RTILDVHRA   61
Zosma88g00080.1_(ZmLUX)                MSQFRQPMEDE-----DVENRVLEWEEGLPSAD--DLTPLFQNMLPPQLAFAFSVTTKPV-KTYQDVNLA   62
XP_010256853.1_(NnLUX1)                MGDEVHTNEYENGIDGGDGERVLEWEAGLPSAD--DLTPLSQPLIPPEIASAFSITPVPC-PSMLDVNHA   67
XP_010262563.1_(NnLUX2)                MGEEVQTTPFDDGLSCGDEARVQEWEVGLPTAD--DLTPLSQSLIPVELASAFSITPEPC-RSIIDVNRA   67
Os01t0971800_(OsPCL1/LUX)              MGEEA---PEEYELGGGEDERVMEWETGLPGAD--ELTPLSQPLVPAGLAAAFRIPPEPG-RTLLDVHRA   64
AT3G46640.3_(AtLUX)                    MGEEVQMSDYDVS---GDGDRVSEWEMGLPSDE--DLASLSYSLIPPNLAMAFSITPERS-RTIQDVNRA   64

Consensus                              SXXTXSXLRRXXSXSXXXSXXXXXXPXFXXX-----------------XXXXXXXXXXXXXDXXXXXXXG
Mapoly0101s0068.1_(MpLUX)              SKSTVQQLQQKKNTAFTLPAFETYPCYESRTGARAAAAGAANGGGGAYATEESMDDERGGYDLTKRSKDD  127
XP_024525426.1_(...without_first_aa    SIATVTALRRQPSSSPGGV-FESIPAFPVA------------------AEEEIDKNAAAGNGIGGFADAG  107
PO025399_(PoLUX)                       SQETMSPLRWHGSESEKSS-SSALKSFRDD------------------AELDVVDEEEEEKEV-------  105
Zosma88g00080.1_(ZmLUX)                SQKTFFNLRRCQASSNSSSTTTATAAYNLLPS----------------LLPSPFLPPSDIADRGCDVSYS  116
XP_010256853.1_(NnLUX1)                SQNTISILYRQSSLSFSSYHLKSLPSFIED------------------STRDAMVVEGEENDAGCSTKDG  119
XP_010262563.1_(NnLUX2)                SHNTLSSLSRQSSQSISSNHLKSLPSFTED------------------RTRDAMVIEGDDNDAGFSAKDG  119
Os01t0971800_(OsPCL1/LUX)              SAATVSRLRRASSSSSSSF-----PAF---------------------------------------ASKG   90
AT3G46640.3_(AtLUX)                    SETTLSSLRGGSSGPNTSS----------------------------------SNNNVEEEDRVGSSSPG  100

Consensus                              XX-------------------------------------------------------------SXXXXXX
Mapoly0101s0068.1_(MpLUX)              SGRRSSRVTKSPMHGSKGGWDGDKASERDVGVSGSGIGGVGNGNSGPDFGPAYYWPAQGSMPGGPGPYGP  197
XP_024525426.1_(...without_first_aa    AGAGAGAGAAANSSLQPGQAGNSFQFQGTQGFDPARRGEVGVASA----------PMMSNLPYSPYERRP  167
PO025399_(PoLUX)                       ----------------------------------------------------------------------  105
Zosma88g00080.1_(ZmLUX)                ES-------------------------------------------------------------SKKARFT  125
XP_010256853.1_(NnLUX1)                SG-------------------------------------------------------------SRKVRRL  128
XP_010262563.1_(NnLUX2)                SD-------------------------------------------------------------SRKVRRP  128
Os01t0971800_(OsPCL1/LUX)              AG-------------------------------------------------------------T------   93
AT3G46640.3_(AtLUX)                    SD-------------------------------------------------------------SKKQKTS  109

Consensus                              XXX---------------------------XEAXSXXXXXXXXXXXXXXTLKRPRLVWTPQLHKRFVDVV
Mapoly0101s0068.1_(MpLUX)              MGSDQGGYGGMEGGSESTKGASLEKSRKLGDTESEDVDSADCPDGNTARTLKRPRLVWTPQLHKRFVDAV  267
XP_024525426.1_(...without_first_aa    ETSSGGGMGAEDSSNTAKKLRKQSSDLGEEEEADSGGGPENSGEEPAARTLKRPRLVWTPQLHKRFVDAV  237
PO025399_(PoLUX)                       ------------------------------EEKDGVLGGGDENKGGS----KRRRLVWTPQLHKRFVDVV  141
Zosma88g00080.1_(ZmLUX)                DSD---------EIPEDFESNTNSVVCGSGNQENSVDESTGAERGVVGSSSKRPRLVWTPQLHKRFVDVV  186
XP_010256853.1_(NnLUX1)                ENA---------------------------EEADSTLPTENSMEDPSARTLKRPRLVWTPQLHKRFVDVV  171
XP_010262563.1_(NnLUX2)                ESA---------------------------EEADSALRTENSMDDHSARTLKRPRLVWTPQLHKRFVDVV  171
Os01t0971800_(OsPCL1/LUX)              -GA---------------------------DEAESGGGADGGNGNTNNSSSKRARLVWTPQLHKRFVEVV  135
AT3G46640.3_(AtLUX)                    NGDGDDGGGVDP------------------DSAMAAEEGDSGTEDLSGKTLKRPRLVWTPQLHKRFVDVV  161

Consensus                              AHLGIKNAVPKTIMQLMNVEGLTRENVASHLQKYRLYLKRMQGLSN-EGP--SXSDXLFASTPVPX-XLX
Mapoly0101s0068.1_(MpLUX)              GHLGIKNAVPKTIMQLMNVEGLTRENVASHLQKYRLYLKRMQGLSN-EGP--SASDPLFASAPLPPNLTQ  334
XP_024525426.1_(...without_first_aa    AHLGIKNAVPKTIMQLMNVEGLTRENVASHLQKYRLYLKRMQGLSS-EGP--SASDHLFASTPVPP-GLA  303
PO025399_(PoLUX)                       AHLGIKNSVPKTITNLMNVEGLTRENVASHLQKYRLYLKGMHRVSN-EDALLSPSDRLFATTPVPQ-NLR  209
Zosma88g00080.1_(ZmLUX)                AHLGLKNAVPKTIMRLMNVDGLTRENVASHLQKYRLYVNRIQ-----DGS--------------------  231
XP_010256853.1_(NnLUX1)                AHLGIKNAVPKTIMQLMNVEGLTRENVASHLQKYRLYLKRMQGLSN-EGP--STSDHLFASTPVPQ-SLH  237
XP_010262563.1_(NnLUX2)                SHLGIKNAVPKTIMQLMNVEGLTRENVASHLQKYRLYLKRMQGLSNDEGP--SSSDHLFASTPVPQ-SLH  238
Os01t0971800_(OsPCL1/LUX)              AHLGMKNAVPKTIMQLMNVEGLTRENVASHLQKYRLYVKRMQGLSN-EGP--SPSDHIFASTPVPHASLH  202
AT3G46640.3_(AtLUX)                    AHLGIKNAVPKTIMQLMNVEGLTRENVASHLQKYRLYLKRMQGLTN-EGP--SASDKLFSSTPVPPQSFQ  228

Consensus                              XXXXX---XXXXXXXPXPX---XPXXXPXPXXXXXXXXXX----XGXXXXXXXXXXXHG-F-XXHPYXXX
Mapoly0101s0068.1_(MpLUX)              SPHFFTNHPHLRAENGVSGPSFSPPAVPMPRAQQAIPPGV----MGPGVMGPGAVQHFGGF-EHHPYSIG  399
XP_024525426.1_(...without_first_aa    AAAAHFIPGHRDDVVALPF---SP-VVPVPIAGL--GAPH----VGAAFG--PRPPYSG-F-EHHPYGTL  359
PO025399_(PoLUX)                       EGPPP-----VPMSMPVPYGMHPPGVIPMPVIGLQYGNGGHVRVMGAANGQLGDGAWHG-FAAHYPYAVF  273
Zosma88g00080.1_(ZmLUX)                ----------------------------------HRGDDF---------------PFFG-----------  241
XP_010256853.1_(NnLUX1)                EPP-----GHGHMPLPIPY---PASLMPTPVLGMAHGHDH----MGVPVGNPGATTYHG-F-ESHPYNMF  293
XP_010262563.1_(NnLUX2)                EPSPH---GHGHMPLPMPY---PPPMMPIPVLGVTHAHGH----MGIPVGNPAATAFHG-F-ESHPYNMF  296
Os01t0971800_(OsPCL1/LUX)              D------------QVPSPY---HPH---------PHHHSY----NNAAYA-ATVSSYHH-Y-HHANH---  238
AT3G46640.3_(AtLUX)                    DIGGGGG-SSGNVGVPIPGAYGTQQMMQMPVY--AHHMGM----QGYHHQNHNHDPYHQ-N-HRHHHGAG  289
```

```
Consensus                           XXXXXX-XXXXXXXXXXXXXXXXXXXX-----------------
Mapoly0101s0068.1_(MpLUX)           LARSAA-QQRPPMGEHREFMAEFEGRA------RVMRNGLQFAT   436
XP_024525426.1_(...without_first_aa GRSVPQ-RMGGGPGDHREMVMENQGQPGSSPPRRILTLFPTSSH   402
PO025399_(PoLUX)                    RQQRGELPCGSKSGSVFPNPHVASDK------------------   299
Zosma88g00080.1_(ZmLUX)             --------------------------------------------   241
XP_010256853.1_(NnLUX1)             REQQRD-WSGNKFGSVVSYPHVTPNDK-----------------   319
XP_010262563.1_(NnLUX2)             REKQ----------------------------------------   300
Os01t0971800_(OsPCL1/LUX)           --------------------------------------------   238
AT3G46640.3_(AtLUX)                 GNGS---WCLQNFLSFAEFSTAKSQIA------SVQERIIQSIM   324
```

Consensus Threshold: >50%

Compare to: the consensus

Amino acids that match the reference are marked with yellow highlighting.

Created: 12 Apr 2023

Last Modified: 12 Apr 2023
