# Supplementary material for: Dim artificial light at night alters gene expression rhythms and growth in a key seagrass species (Posidonia oceanica)
Source: Sci Rep. 2023 Jun 30;13:10620. doi: 10.1038/s41598-023-37261-3 (PMC10313690; doi:10.1038/s41598-023-37261-3)
Supplement: Supplementary file 6 — Supplementary Information 6. [file 41598_2023_37261_MOESM6_ESM.pdf]

```
Consensus                     ----------------------------------------------------------------------
Mapoly0122s0007.1_(MpPRR)     MTAESSEKEAESRLKEDFSPEVSNTGGQDGDPSGPREGLEGGRNWDNGNSKDRDRDAGFGNGYMNNGFSG  70
XP_002972852_(SmPRR7a)        ----------------------------------------------------------------------
XP_002979868_(SmPRR7b)        ----------------------------------------------------------------------
PO007080_(PoPRR5-2)           ----------------------------------------------------------------------
PO053273_(PoPRR5-1)           ----------------------------------------------------------------------
Zosma4g02070.1_(ZmPRR5)       ----------------------------------------------------------------------
XP_010251440.1_(NnPRR5-1)     ----------------------------------------------------------------------
XP_010262980.1_(NnPRR5-2)     ----------------------------------------------------------------------
XP_010246868.1_(NnPRR5-3)     ----------------------------------------------------------------------
Os11t0157600_(OsPRR59)        ----------------------------------------------------------------------
Os09t0532400_(OsPRR95)        ----------------------------------------------------------------------
AT5G24470.1_(AtPRR5)          -----------------------------------MWQTWPRQPILLDIFSNPNTLSTTVRSWSVRHPLS  35
```

```
Consensus                     ---------------------------------------------XXXXXXXXXXXXXXXXXXXXX----
Mapoly0122s0007.1_(MpPRR)     SSDLNVEAKESRADVEKAARMDIDAEVQGRPDKKKRLPTRDVRVSDTTPSTEEDRDEETEDDFEEGDCVM  140
XP_002972852_(SmPRR7a)        -----------------------------------------------MTMRQAGILDAPNGA--------  15
XP_002979868_(SmPRR7b)        --------------MNKVEGVLVNGTTTTTTPTIASSVSCALRGEASGENGKGAQGSNENGAPPTNNSSD  56
PO007080_(PoPRR5-2)           ---------------------------------------------MDGGVRGGERPPDPFPSSAAG----  21
PO053273_(PoPRR5-1)           ----------------------------------------------------------------------
Zosma4g02070.1_(ZmPRR5)       ----------------------------------------------------------------------
XP_010251440.1_(NnPRR5-1)     ----------------------------------------------MGEFTVSSEDAMEVVLQEEE----  20
XP_010262980.1_(NnPRR5-2)     --------------------------MSSCLLGCWKNMEHRMREEAEVVVAGDDGEEVATGRSMENRVVR  44
XP_010246868.1_(NnPRR5-3)     -------------------------------------MERREGEGGEVVVAGGQAEEVASGREMERLVVR  33
Os11t0157600_(OsPRR59)        --------------------------------------------------MSPDADAAAAAAAGGE----  16
Os09t0532400_(OsPRR95)        --------------------------------------------------MGGGVEERKVVDL-ED----  15
AT5G24470.1_(AtPRR5)          IITVKTFARFFLDIFFSPHYYRKNKVLFFALFSFISPLTNILICFVTVSLSLELSSSSSIIDLGFSKLSV  105
```

```
Consensus                     ----------------------GXXXXXXXXXXXXXXXXXXX---------------------------X
Mapoly0122s0007.1_(MpPRR)     ADAASKHHPQQGPSHPQAEEQPGKGDRSQCQKQQQQEEQQPQAQPKQEPHGQEAREQQQQPPPKAVVVPA  210
XP_002972852_(SmPRR7a)        ----------------------GAGI----GGESGKLSQRG----------------------------E  31
XP_002979868_(SmPRR7b)        DNDSGNDLKNAEPNSKIHAGNQKEEQELQPEGEEKHRHRQQ----------------------------Q  98
PO007080_(PoPRR5-2)           ----------------------GAAGDGNKGGEHQRQQQREM---------------------------I  42
PO053273_(PoPRR5-1)           ------------------------------------MEENGV---------------------------I  7
Zosma4g02070.1_(ZmPRR5)       ---------------------------MERGERNLEGEGRGV---------------------------V  16
XP_010251440.1_(NnPRR5-1)     ----------------------GEGETQEDKKKDSSATM------------------------------V  38
XP_010262980.1_(NnPRR5-2)     DVVAGAVEASSRNRVSNGELNSGGGG----GGGGGSRSREQV---------------------------I  83
XP_010246868.1_(NnPRR5-3)     HVIAGTL---------------GASSRNRDSTTAGGGGGEEQV--------------------------I  62
Os11t0157600_(OsPRR59)        ----------------------GAAA----AGVGTAGEGRGV---------------------------I  33
Os09t0532400_(OsPRR95)        ----------------------GDGE----EGEDAAAVAAGS---------------------------S  32
AT5G24470.1_(AtPRR5)          CVVIMTSSEEVVEVTVVKAPEAGGGKLSRRKIRKKDAGVDGL---------------------------V  148
```

```
Consensus                     XWEXFLPRXXLRVLLVEXDDSTRHIVAALLRKCGYXVXAXADGLKAWEXLKXKXXXXDLVLTEVXMPXLS
Mapoly0122s0007.1_(MpPRR)     GWESFLPRKHIRVLLAEDDDSTRHVVGALLRNCGYEVTSAANGMQAWEMLEDPNNFFDLVLTDVVMPCLS  280
XP_002972852_(SmPRR7a)        GWENLPPYRQLKVLLVEDDDSTRHVVAALLRNCGYQVVPAANGLQAWSLLDDRNREIDLVLTDVVMPGLS  101
XP_002979868_(SmPRR7b)        QAENAFQHHHLKVLLVEDDDSTRHVVAALLRNCGYEVTPAASGLQAWEILETRSSSVDLVLTDLMMPRLS  168
PO007080_(PoPRR5-2)           RWERFLPRRSLRVLLVEHDDSTRHIVTALLRKCNYHVAAVADGLKAWEVLEEKQYRFDLVLTEVEMPSLS  112
PO053273_(PoPRR5-1)           RWERFLPRAFVRVLLVEKDDSTREIVAALLRKCGYRVVLAADGLKAWEMIKEKTQQIDLVLTEVELPHMS  77
Zosma4g02070.1_(ZmPRR5)       RWEMFLPRPFVRVLLVEGDNSTREIVSVLLRKCGYRVSVAADGVKAWETLKRKGHEIDLVLSEVDLPRRS  86
XP_010251440.1_(NnPRR5-1)     KWEEIFPRMFLRVLLVEADDSTRQIIAALLRKCSYRVSAVPDGLKAWEALKGRPHKIDLILTEVELPLIS  108
XP_010262980.1_(NnPRR5-2)     GWERFLPRRFLRVLLVENDDSTRHIVAALLRKCSYQVTAVADGLKAWEVLKEKQYNFDLVLTEVVMPSLS  153
XP_010246868.1_(NnPRR5-3)     RCERFLPRRSLRVLLVESDDSTRHIIAALLRKCSYKVAAVADGLKAWEFLKEKHYNFDLVLTEVVMPSLS  132
Os11t0157600_(OsPRR59)        RWDQILPRRSLRVLLVEHDDSTRQVVTALLRKCGYRVAAVADGMKAWGVMRERAYAFDLVLTEVTMPTLS  103
Os09t0532400_(OsPRR95)        RETRMLPRMPVRVLLAEGDDSTRHIICALLRKCGYRVAAASDGVKAWDILKEKSFNIDLVLTEVELPLMS  102
AT5G24470.1_(AtPRR5)          KWERFLPKIALRVLLVEADDSTRQIIAALLRKCSYRVAAVPDGLKAWEMLKGKPESVDLILTEVDLPSIS  218
```

Sequence	Alignment	Position
Consensus	GXXLLXXIMXXXXCKNIPVIMMSSHDSXXXVFKCMXKGAADFLVKPVRKNELRNLWQHVWRXXXSXS---	
Mapoly0122s0007.1_(MpPRR)	GVGLLTKIMSREAGKRIPVIMMSSHDSLDIVYRCLFKGAADFLVKPVRKNELKNLWQHVWRCQSSSGSGS	350
XP_002972852_(SmPRR7a)	GLGLLSKIMHHKNHQKVPVVMMSSHDSTNVVFKCLTKGAADFLVKPVRKNELKNLWQHAWRKARSSS---	168
XP_002979868_(SmPRR7b)	GIELLGKIMSRDSPKRIPVVMMSCLDSMDVVLKCLSKGAVDFLVKPVRKNELKNLWQHFWRMCLSSS--G	236
PO007080_(PoPRR5-2)	GIGLLSKIMGSELSKNIPVIMMSSHDSVSVVYKCMLKGAVDFLVKPVRRNELRNLWQHVWRKHCSTS---	179
PO053273_(PoPRR5-1)	GFALLTFIMEHESCKSIPVIMMSSHDSMNMVFKCMLKGAADFLVKPIRKNELRNLWQHVWRRYISNG-ES	146
Zosma4g02070.1_(ZmPRR5)	GFCLLTMIMEHESCKNIPVIMMSSNDNINMVFNYMQTGAADFLVKPVRKNELRNLWQHVWRRCIPNS-DS	155
XP_010251440.1_(NnPRR5-1)	GFALLTLIMEHEICKNIPVIMMSSHDSISMVFKCMLRGAADFLIKPIRKNELRNLWQHVWRRQTSTG---	175
XP_010262980.1_(NnPRR5-2)	GIGLLCKIMSNDICENIPVIMMSSHDSIGIVFKCMQKGAADFLVKPVRKNELRNLWQHVWRKYCSSS---	220
XP_010246868.1_(NnPRR5-3)	GIGLLSKIMNNEICKSIPVIMMSSHDSIGIVFKCMQEGASDFLVKPVRKNELRNLWQHVWRKYSSNS---	199
Os11t0157600_(OsPRR59)	GIELLSRIVASDECKNIPVIMMSSQDSIGTVLRCMQKGAVDFLVKPVRKNELRNLWQHVWRRHAMNS---	170
Os09t0532400_(OsPRR95)	GFLLLSTIMEHDACKNIPVIMMSSNDSVSMVFKCMLKGAADFLVKPIRKNELRNLWQHVWRKQLSSGVLD	172
AT5G24470.1_(AtPRR5)	GYALLTLIMEHDICKNIPVIMMSTQDSVNTVYKCMLKGAADYLVKPLRRNELRNLWQHVWRRQTSLA---	285

Sequence	Alignment	Position
Consensus	XGXXXXXXX--XXXXXXXXXXXNXXASNHXXXXXXXXXXXXXXSDXGSDTQSSCTKXXXEXXSXXX--XX	
Mapoly0122s0007.1_(MpPRR)	GGSGSGSGMRKFMQPKSRVGSGNNSGSNDGSEDGSSGLNVRGGSDNGSGTQTVAEHVQTREKQKRD----	416
XP_002972852_(SmPRR7a)	-GSESETGRL------------NKCGGSCGELSSDSGSEEIRISDQ-SDTESSCTKKVAELESPRKSRPR	224
XP_002979868_(SmPRR7b)	SGSGSETG---------KAAGGNNNSQSYGGSEHGSGLNVGGGSDNGSGTQSSWTKKPVEVQSHAT--EE	295
PO007080_(PoPRR5-2)	CAQGQESIN--NNTIKSGDMSGNNAASNHVSAVAGSMSKNKEWCDEGSDTQSSGSKPEQEIQSVAR--PR	245
PO053273_(PoPRR5-1)	GGSDASPGVN-LAKQKCKDNLDNDAASNHSDENLACFERKGECSDKGSDTQNSCTRQGMEAESTCM--QN	213
Zosma4g02070.1_(ZmPRR5)	GGRDLNKNTN-LSQPKYENDTG-KSASIHSSENPLCSMRKVECSDKGSDTQTSCTRQSMEAQSTFP--QN	221
XP_010251440.1_(NnPRR5-1)	SGHGHQEGR--PVQQKVEAISENNAASNHSSDYVARTQKNKQCSEKGSDTQSSCTKPDRQAENAYI--QN	241
XP_010262980.1_(NnPRR5-2)	CGNRSENDN--LNQVRLEAVSDNDAASNHGSANACNKSKMGNSSNKESDTQSSCSKPETENKSLQKIGES	288
XP_010246868.1_(NnPRR5-3)	SGNWYGGGT--PNQVRFEAVSDNDAASNHRNANAGNKSKTGNRSDKESDTQSSCSKPETENEIVRKPGES	267
Os11t0157600_(OsPRR59)	----------------QTNASENNAASNHLSANGGNGSKTGEHSDEESDAQSSGSKREVEIQSAEKLPEV	224
Os09t0532400_(OsPRR95)	VQHTQQED---NLTERHEQKTGVTKAEHVTENVV---HKNMECSEQESDAQSSCTRSELEADSRQT--NN	234
AT5G24470.1_(AtPRR5)	-----------PDSFPWNESVGQQKAEGASANNSNGKRDDHVVSGNGGDAQSSCTRPEMEGESAD-----	339

Sequence	Alignment	Position
Consensus	XXXXXXXXXXX-----------------XXXXXXXXXXXXXXXXXXXXX----XXXXXXXXXXXXXXXXX	
Mapoly0122s0007.1_(MpPRR)	-QEEGQATSLQKASDE------------QMGQDLEMATRRPGVVELQEA------EQEKQLNDSAALDNG	467
XP_002972852_(SmPRR7a)	A---------------------------FTKPKPCPPSRRARRTKDSDT---------------------	246
XP_002979868_(SmPRR7b)	LVLQ------------------------RDKREEIDIDRDCGEVAMAEK-----SRTASQLLGGHLLGAK	336
PO007080_(PoPRR5-2)	VTNDAEHMNSSKVLESKLEREQNHKL--LLETKSMKHNSRGERNDIAEI---REEAISKRKFGEQDFVAE	310
PO053273_(PoPRR5-1)	MQKQNQMNLTS-----------------TSVNSAMRIHGIAECINLKKV---VPIQDEAGGKSATLFPEV	263
Zosma4g02070.1_(ZmPRR5)	AKVQNKMNSTS-----------------FNSKEVLVLHDDTEGKPMGST----VEVSSFNQTNEEHTLGG	270
XP_010251440.1_(NnPRR5-1)	MQDQLQPKRRS-----------------ASPVSDSHIQKHGGCIDQNLL-----VHENRTEGKDQDYVRM	289
XP_010262980.1_(NnPRR5-2)	LEIENKGFPKE--TDLKLEKHEYNMVTESSMLDKGKAEDKSTGVEVKVTLSIQGAASNKRIWEKNVFYAG	356
XP_010246868.1_(NnPRR5-3)	LETENRSFLKE--TNSILEKHEYNMVTASSMHERGKSEDKSTRLEIKVS----KTASNKEVWEKNGIYSG	331
Os11t0157600_(OsPRR59)	VADGGAGSSREHKIQNGFIDGMNTKSHALKGNDDAPSGNACGDSELQVL----STEKNVRSKFLNGITSA	290
Os09t0532400_(OsPRR95)	LLEYKQPMGRH-----------------FSKPDHKNTEKNGGTKIHASN-----DGNLIPRREEDASLRR	282
AT5G24470.1_(AtPRR5)	----------------------------VEVSARDAVQMECAKSQFNET---------------------	360

Sequence	Alignment	Position
Consensus	XXXXXXXXXXXXXXXXXXXXXXXXXXXX-----XXXXEXXDLXGXXXXXXXX--XXXXXXXXXXXXXXXX	
Mapoly0122s0007.1_(MpPRR)	SPSSSNRNDVDQMEEGYNSGAANS---------GGSAKAIDLIGGIACQNNE--DPKEGEDSDGVEGSDR	526
XP_002972852_(SmPRR7a)	---VDDVGARLKPADSPADT-------------DRAMEAVDLIGNIAEPSTA------KAAEEFESEMGA	294
XP_002979868_(SmPRR7b)	STISSSITIFERAPSLAAGGKSGG---------GGEPSCKDLLASIQKQAAF---------EQPGEKLDS	388
PO007080_(PoPRR5-2)	ATETNLNNKEDLNDDTRVPGTFDSNLQI-----KTSNQIIDPIGTVANGECS--YAVMEKDNHREEYQFE	373
PO053273_(PoPRR5-1)	TSCNQAADAGVDYQDSIPPTGFNDNCDQSIW--EKSTEIADLLGAMDNKAGC-----VPESDPITDDSIP	326
Zosma4g02070.1_(ZmPRR5)	NSAKDEINHPNRLSSMRSNDYRQTEGSNWYNKMKPSVEVADLLGAMDSKTKR--ILILNNDTNIFDNTMN	338
XP_010251440.1_(NnPRR5-1)	ITHSDDVAPETQSEDVYINTKTQSGYHEK----VPLREAIDLIGAFDNHRSY------DNTYRTVQEVVS	349
XP_010262980.1_(NnPRR5-2)	SPCREEQPVSLRNKEGAHNDPVSLDQTEGTN--EPSKEVIDFIGAATAQQYS--CSVLGNNGHKEVILSE	422
XP_010246868.1_(NnPRR5-3)	SLCRGEWSTSMRNKEGAANNPESLDQTEGTK--EPPREIINFIGVETSKQCSSPSPVLGNNDNGKVVLCQ	399
Os11t0157600_(OsPRR59)	KVAGQIMDNALRFADSSSLRSS-----------DPGKDLLVVAQTTADRKCK--SSALENNAVMENNLSE	347
Os09t0532400_(OsPRR95)	MTCSNDINCEKASRD------------------------MELVHIIDNQQKN--------NTHMEMDVAR	320
AT5G24470.1_(AtPRR5)	----RLLANELQSKQA---------------------EAIDFMGASFRRTGR---------RNREESVAQ	396

```
Consensus                   XXXXXXXXXXXXXX-----XXXXLELSLXRXXX-XXXXXXXXXXXXXLXHSXXSAFSRYXXXXXXXXXXX
Mapoly0122s0007.1_(MpPRR)   GASNSPVMNDKYASGT---SMPTLELSLKRSRV-PGDDDVEVEERRAVRQSGGSAFSRYSTSGVIIQQHL   592
XP_002972852_(SmPRR7a)      SNSPEVILERDVSSSDPAPTSGNLELALKSPTP-----DHAYEPSYVLNQSGPSAFSRSNDQAAAAAAAA   359
XP_002979868_(SmPRR7b)      KDNSVPSNDS-------------ALELSLKRTRV-KGPDDGDGEQKRLLHHSGGSAFSRYSNNPLLQHNST   445
PO007080_(PoPRR5-2)         AIKSLHRTIGSTSD-----ALLSWELSLRGPHL-NVSSKEDSQERHILKHSAASAFSRYCAGIHFTYQKS   437
PO053273_(PoPRR5-1)         KDSGILIEDNN-----------QLELSLKRYQP-QNCDGTETNGCNMLSHSSYSAFSRYNSRNVLPNFPP   384
Zosma4g02070.1_(ZmPRR5)     NPSSVIDDSD------------QLQLSLKRYQPSRKCIEIESNGSKMLSHSNSSAFSRYSSSKVVQNSRY   396
XP_010251440.1_(NnPRR5-1)   CDTKIKSGLDDDQLG---PTPLLELSLRRSYT-IDPEKQRTDIRHALNHSNFSAFSWYNNRVMQQCVPL   414
XP_010262980.1_(NnPRR5-2)   AAKTSDVKSSISNSG----SLPLWELSLRRSQF-KGQDEEVFQEKHVLNHSDASPFSRYASAQIRTSCPR   487
XP_010246868.1_(NnPRR5-3)   PAKSFDARSRMCTSG----PFPLRELSL--EHV-KHQNGGVFQEKHVLNHSDASPFSRYAGSRIHVSSSR   462
Os11t0157600_(OsPRR59)      NSKGTATGHAESC------PSHFVEINLEKQHHLNGYTNHKLNEKDIFNHSNSSAFSRYGNKRIESSAQR   411
Os09t0532400_(OsPRR95)      ANSRGNDDKCFSI------PAHQLELSLRRSDY-SRLESQEKNERRTLNHSTSSPFSLYNCR---TASST   380
AT5G24470.1_(AtPRR5)        YESRI-----------------ELDLSLRRP---NASENQSSGDRPSLHPSSASAFTRYVHRPLQTQCSA   446
```

```
Consensus                   XXXXXXXXXXXXXXXX----------XXXXXXXXXXXXXXX--------------------XXXXXXXXX
Mapoly0122s0007.1_(MpPRR)   PSGNNLALGTYSMGGAYSLSAGQSGLASSSKTGQLHHSTAFDRCGSFMGSGEASTPHANPPHLPPKGSGQ   662
XP_002972852_(SmPRR7a)      TAAATA--------------------AFFARPEQQHHIVTP-----------------------------   380
XP_002979868_(SmPRR7b)      LSSSIAANLWFGNEKS----------TPAAAADQNHHHANA------------------AAAAAEVLKPP   487
PO007080_(PoPRR5-2)         DSSPSTLPITTKAHGD----------KIESQLSNHIACEEEVPTPTWRLLTTAPENESEGITNLQASTATN   497
PO053273_(PoPRR5-1)         MNCRVGKEDVLGYHCQ----------YQLNQDPPNPTCSQL---------------------------SG   417
Zosma4g02070.1_(ZmPRR5)     ---VPNSSCTPRPHPE----------ATESTMISSSQDRLP-----------------------------   424
XP_010251440.1_(NnPRR5-1)   SASCATEQSERSINSD----------KQLSRQDNAKTSDAS---------------------QQNGISS   452
XP_010262980.1_(NnPRR5-2)   SGSSSANLCVRASDSD----------AAKSHTNSSSNHGNKIPFPLIERLISPNQNGDETLICCHLSSSS   547
XP_010246868.1_(NnPRR5-3)   SGSSSASLCIKTDGSS----------SVKSHINSSSNDGKS-------------------ICYYISSSS   502
Os11t0157600_(OsPRR59)      PFPPSFRVVHQQPVYD----------KNPQSSRVLLSCEHNTRESTVQAQVPLDRSTEGAAILCSSSV--   469
Os09t0532400_(OsPRR95)      INAGDAQACSTSATHI----------DLENKNGDSKTPSQD-----------------------------   411
AT5G24470.1_(AtPRR5)        SPVVTDQRKNVAASQD----------DNIVLMNQYNTSE----------------------PPPNAPR   482
```

```
Consensus                   XXXXXXXXXXXXXXXX------------XXXXXXXXXXXXXXPXPXXXXXFXXXXXXYGXXXXPXFYXX
Mapoly0122s0007.1_(MpPRR)   DLGSGVVGTSGQDGYANSRQIKDEAASASPASAPGIPISRAGVPPPA--IIYDGVCASYGPPMHPSFYSH   730
XP_002972852_(SmPRR7a)      ------------------------------VQYATVLSYDQSLPARG----GATYYYTQPALPPWQHA   414
XP_002979868_(SmPRR7b)      KAEAAAVAAAAA----------------AAAAAASQFKLAASDF----VAFNDIAAAYAQSLHHFFYHG   536
PO007080_(PoPRR5-2)         HEDDGRFATGPS-GEDACEPRTSTEDNNCNLTFPSCQPGLVSHPFPVRGMPFQSLCAGYGTYMQPVFYHD   566
PO053273_(PoPRR5-1)         TMESTVISSSSQD---------------GKAVQCLQTDCTPFPVPA--VPY-SLFAGFGFSLQPIVSRE   468
Zosma4g02070.1_(ZmPRR5)     ---------------------------AQHSCDNLLARFPFSQQSITIQKSASTNSNHFVP---RT   460
XP_010251440.1_(NnPRR5-1)   NNNHESMTSLVRQSGPS-----------ETVFRCSPLGVVHMPVSVRSMTFDDLRSSYGALIPPIFYTQ   510
XP_010262980.1_(NnPRR5-2)   KREDGEPSSSVTLREDTCVGHSSTE----KTVFSHPQLGFIPLPIPVGTTPFHNLCAGYGTIFQPVFYPE   613
XP_010246868.1_(NnPRR5-3)   RNEDGEPSSSVTLREDACVGHSFTE----NIVFPYPQLGFIPLPVPVGAMPFQNLCAGYNTILQPTFYQE   568
Os11t0157600_(OsPRR59)      REDAGTSSSSPR----------------KDSLTHPSYGFIPVPIPVGA----AIPYHYGAIMQPMYYPQ   518
Os09t0532400_(OsPRR95)      ---------------------------KRETNQPPIRVVPFPVPVGGLTFDGQPFWNGAPVASLFYPQ   452
AT5G24470.1_(AtPRR5)        RNDTSFYTGADSPGPPFS-----------NQLNSWPGQSSYPTPTPINNIQFRDPNTAYTSAMAP---AS   538
```

```
Consensus                   XXX-PXX-----------XXXXXXXXXXXXXXXXXXXXXXXXXXX---------------XXXXXXXXXXX
Mapoly0122s0007.1_(MpPRR)   PAA-PPWA-------TGPAHKSNRGEVFDHPPYREHGTHASHHH---------SHHHQPHRSQHHHHHHT   783
XP_002972852_(SmPRR7a)      FSP-PQQQPRLDNPDFVEQQQRQQQHFVEQQQRQQQQQHEEQKH--------RSFQALTNIMAHHHHYHI   475
XP_002979868_(SmPRR7b)      SSP-PAWPPPMLAAPHQQQQQQQQQQQLQHHIQLQQQQQQHHQEQQQDQSQKQQQQQQQQQQQHHHHHHH   605
PO007080_(PoPRR5-2)         SFL-AHG----------SHGPEKE----EASKPVDLNHHLVYNLPSEMLQHCKEGSRASPLEGQKDRMDD   621
PO053273_(PoPRR5-1)         LGP-PPW-----------STKPTIQKAIPVNFPKQSGPKILDSI--------------KSCDSHDQSGKI   512
Zosma4g02070.1_(ZmPRR5)     NGL-DP-------------------------------------------------------SEETE   470
XP_010251440.1_(NnPRR5-1)   SSPIPVW--------GQSSPVNQWELTVPLNSSLHSNPETCN-----------------SRQGYHPHSQN   555
XP_010262980.1_(NnPRR5-2)   TST-PLC-----------GSSEVGKDVLHPSNPYSQDSHIVNDL---------------KHDGSHDCVEK   656
XP_010246868.1_(NnPRR5-3)   TST-LLC----------ESSEMGRQDALHPPNQCVHDNHFTNDF---------------NSDTCHHCVEN   612
Os11t0157600_(OsPRR59)      GAF-MHC----------DSAAINKTAIQHVSCQSNYHENLGK---------------PPQIDEHKQPEEN   562
Os09t0532400_(OsPRR95)      SAP-PIW----------NSKTSTWQDATTQAISLQQNGPKDTDT---------------KQVENVEEQTAR   497
AT5G24470.1_(AtPRR5)        LSP-SPS---------------------------------------------------SVSPHEYSSMF   555
```

```
Consensus                     XXXXXXXXXXXXXXX-------XXXXXX------------XXXXX---------X---------------
Mapoly0122s0007.1_(MpPRR)     NLPQSSVRSMQDDQT-------VQNPGA------------GAPRCGSSNMAGSVP---------------  819
XP_002972852_(SmPRR7a)        EHHHHDKASGSNELE-------TPGCGSTNVAGDGSGGVGGGELHSSLH-----A---------------  518
XP_002979868_(SmPRR7b)        HHHHHQHAQQPQEEDELTVNNNTNTSNNNTTNNANLMRVGSTQQCGSSNVSVKAPKAGVGGGATNNSGGN  675
PO007080_(PoPRR5-2)           EELGQRSLMYTDTEQ-------TKKSCACSHNNTNGCSELGTRSFM--------C---------------  661
PO053273_(PoPRR5-1)           ENCQAEHAHVGNFEF-------TEDLRN--------------------------L---------------  534
Zosma4g02070.1_(ZmPRR5)       KIYQSENAELQNLEC-------IEDP-----------------NNI--------P---------------  493
XP_010251440.1_(NnPRR5-1)     ENYANKHKVHELEEH-------NMESAEHPGD----------------------V---------------  581
XP_010262980.1_(NnPRR5-2)     PHPHRLKHPMEESRD-------NNKSIY------------PRKML---------P---------------  683
XP_010246868.1_(NnPRR5-3)     PHPHRLTHSIKESGD-------NSKPTY------------PRTMF---------P---------------  639
Os11t0157600_(OsPRR59)        HQLHHSRQILRESGE-------PVDLA---------------------------K---------------  583
Os09t0532400_(OsPRR95)        SHLSANRKHLRIEIP-------TDEPRH--------------------------V---------------  519
AT5G24470.1_(AtPRR5)          HPFNSKPEGLQDRDC-------SMDV--------------DERRY---------V---------------  580

Consensus                     -SXXXXXSXXXXXXXXXXX-XXXSGX-------------------------------XXXXXXXXXXXXX
Mapoly0122s0007.1_(MpPRR)     -DGNTGQSGSSNGNNGSVN-GSASGSNN-----------------------------LSNENGQSGVPLP  858
XP_002972852_(SmPRR7a)        -SNNNGGSGNGNGWSSYGNNGSVSGS-------------------------------NHGSNNVYNDSTA  556
XP_002979868_(SmPRR7b)        EEASNGGSSNGCGVNGTNGSVNGSVSGSNNLSANNNNIHKGQGSCSLAAGNGDSGGTNNGSSRGGNATAA  745
PO007080_(PoPRR5-2)           -SDIEQTNKNCACCRNN---LNGNGS--------------------------------NGIDGTTDAAVN  695
PO053273_(PoPRR5-1)           -STVTDQSGSSSINGSNNVNSTGVGS-------------------------------VCNGYSDSMAAQN  572
Zosma4g02070.1_(ZmPRR5)       -SPATCCNRGGSVCNGSVN-IKGSGIAY-----------------------------EDGTL------TK  526
XP_010251440.1_(NnPRR5-1)     -SPATGNSASSSLCNGAVSHLNSSGCGS-----------------------------ICNGSNGNVTPAA  621
XP_010262980.1_(NnPRR5-2)     -TPTDIANQLANCSHGITD--------------------------------------ANGSNDSNRYTVS  714
XP_010246868.1_(NnPRR5-3)     -KPTEMADQLGICNHGI---TDASGS--------------------------------NGSDEDASAAAN  673
Os11t0157600_(OsPRR59)        -AHMERINQSASCSQDI---RKGSGC---------------------------------TGSGETDANTN  616
Os09t0532400_(OsPRR95)        -SPTTGESGSSTVLDSARKTLSGSVCDS-----------------------------SS----NHMIAPT  555
AT5G24470.1_(AtPRR5)          -SSATEHSAIGNHIDQL-----------------------------------------------------  596

Consensus                     XXXXXXXXXXXGXXXXXXXXXX-----XXXXXXXRREAALXKFRLKRKDRCFEKKVRYXSRKKLAEQRPR
Mapoly0122s0007.1_(MpPRR)     PNTNAGCGGNNGESGSGNGAVNAGAVDPEQNRFARREAALNKFRQKRKERCFEKKVRYQSRKRLAEQRPR  928
XP_002972852_(SmPRR7a)        KMSNFGSCCNGGDSNPGSNNCGAPAENAANNSKVRREAALNKFRQKRKERCFEKKVRYQSRKRLAEQRPR  626
XP_002979868_(SmPRR7b)        TGVTNGSNTSNTINEAGGATQV-----PDSARLTRREVALYKFRQKRKERCFEKKVRYQSRKRLAEQRPR  810
PO007080_(PoPRR5-2)           RGMALGNDIENRLQNHERKEL----------NQVRREAALIKFRLKRKDRCFEKKVRYHSRKKLAEQRPR  755
PO053273_(PoPRR5-1)           TIKATGESCNDERVLSQGGIKL-----TQSHRSIEREAALTKFRLKRKDRCFEKKVRYHSRKKLAEQRPR  637
Zosma4g02070.1_(ZmPRR5)       DGIRVTKESDNGNIFCSGEVIVP----IRPNRSIEREAALLKFRLKRKERCFGKKVRYHSRQKLAEQRPR  592
XP_010251440.1_(NnPRR5-1)     AGRTTTESETDECIFIHDGIRG-----INSHRSTEREAALTKFRLKRKERCYEKKVRYQSRKKLAEQRPR  686
XP_010262980.1_(NnPRR5-2)     TGAAVENGNEDGSINFNRKVE---------HDHSRREAALTRFHLKRKDRCFEKKVRYHSRQKLAEQRPR  775
XP_010246868.1_(NnPRR5-3)     TGISVESGNEDGFRNCKGKVLE--------RDCSRREAALIRFRLKRKDRCFEKKVRYHSRQKLAEQRPR  735
Os11t0157600_(OsPRR59)        TVIALESGNESGVQNCSNNVLD--------GDRSRREAALLKFRMKRKDRCFEKKVRYHSRKKLAEQRPR  678
Os09t0532400_(OsPRR95)        ESSNVVPENPDGL-----------------RHLSQREAALNKFRLKRKDRCFEKKVRYQSRKLLAEQRPR  608
AT5G24470.1_(AtPRR5)          ----IEKKNEDGYSLSVGKI----------QQSLQREAALTKFRMKRKDRCYEKKVRYESRKKLAEQRPR  652

Consensus                     VKGQ---------------------FVRQXXXXXXXXXXXX--------------
Mapoly0122s0007.1_(MpPRR)     VRGQ---------------------FVRQTVYDVNTGEAVE--------------  948
XP_002972852_(SmPRR7a)        VRGQ---------------------FVSQAVFDS---------------------  639
XP_002979868_(SmPRR7b)        IKTKINDNNEAPEDHLTSKHQDEEELSRKELLGRDWKNSGGRSEGLAMVDDCELT  865
PO007080_(PoPRR5-2)           IKGQ---------------------FVRQSATTEDD-------------------  770
PO053273_(PoPRR5-1)           VKGQ---------------------FVRQGQPDPKLIEVDSHQSKTMAT------  665
Zosma4g02070.1_(ZmPRR5)       VKGQ---------------------FVRQVQSMEVKSNLNG--------------  612
XP_010251440.1_(NnPRR5-1)     VKGQ---------------------FVRQMPMQPSIC------------------  702
XP_010262980.1_(NnPRR5-2)     LKGQ---------------------FVRQSVAESTMMAAQPDN------------  797
XP_010246868.1_(NnPRR5-3)     LKGQ---------------------FIRQSVAESTAITAEAES------------  757
Os11t0157600_(OsPRR59)        VKGQ---------------------FVSQKLKSAITTEAETD-------------  699
Os09t0532400_(OsPRR95)        VKGQ---------------------FVRQDHGVQGS-------------------  623
AT5G24470.1_(AtPRR5)          IKGQ---------------------FVRQVQSTQAP-------------------  667
```

Consensus Threshold: >50%

Compare to: the consensus

Amino acids that match the reference are marked with yellow highlighting.

Created: 12 Apr 2023

Last Modified: 12 Apr 2023
