# Supplementary material for: Dim artificial light at night alters gene expression rhythms and growth in a key seagrass species (Posidonia oceanica)
Source: Sci Rep. 2023 Jun 30;13:10620. doi: 10.1038/s41598-023-37261-3 (PMC10313690; doi:10.1038/s41598-023-37261-3)
Supplement: Supplementary file 7 — Supplementary Information 7. [file 41598_2023_37261_MOESM7_ESM.pdf]

```
Consensus                      -XXXXXXXXXXXXXXXX-------------------XPXXXDXXXK----KVRKPYTITKSRESWTEXEH
Mapoly0042s0058.1_(MpRVE1)     --MLGVMAAGSPALSGM-------------------GPQGSEEGSK----KVRKPYTITKSRESWTEQEH   45
SELMODRAFT_78045_(SmRVE)       --------MANPALPSD------------------------DAVSK----KIRKPYTITKSRESWTEQEH   34
PO057195_(PoRVE1)              MCVTPADTSTSPTLKDVKMSSTSNN-----------QPKGSSSLGEEHIPKIRKTYTITKQRERWTEEEH   59
Zosma38g00570.1_(ZmRVE8)       --MLPVTTVGKDDGSGSFIAPAPVSGIGLSQNTEFVSDDATTTTTK----KVRKPYTIKKTRESWSEQEH   64
XP_019053625.1_(NnRVE8-1)      -MNVNSTSSNSQSMAAS-------------------TPL--DASGK----KVRKPYTITKSRESWTEEEH   44
XP_019053626.1_(NnRVE8-2)      -MNVNSTSSNSQSMAAS-------------------TPL--DASGK----KVRKPYTITKSRESWTEEEH   44
LOC_Os06g01670.1_(OsRVE8)      -----MSSAPQQLLDSSSPGPGP-------------GPEVEDDGGR----RVRKPYTITKSRESWTDPEH   48
AT3G09600.1_(AtRVE8)           MSSSPSRNPTNAEAPPP-------------------PPTSTDAVAEGSSKKVRKPYTITKSRESWTEEEH   51

Consensus                      DKFLEALQLFDRDWKKIEXFVGSKTVIQIRSHAQKYFLKVQKNGTXXH--------VPPPRPKRKAAXPY
Mapoly0042s0058.1_(MpRVE1)     DKFLEALQLFDRDWKKIEAFVGSKSVIQIRSHAQKYFLKVQKNGTGEH--------VPPPRPKRKSAQPY   107
SELMODRAFT_78045_(SmRVE)       DKFLEALQLFDRDWKKIEAFVGSKTVIQIRSHAQKYFLKVQKNGTGEH--------VPPPRPKRKSAQPY   96
PO057195_(PoRVE1)              NMFLEALKLHGRAWRRIEEHIGTKTAVQIRSHAQKFFSKVVRESSGNNTSSVKPIVIPPPRPKRKPMHPY   129
Zosma38g00570.1_(ZmRVE8)       EKFIESLQLFDRDWKKIEAFVGSKTVIQIRSHAQKYFLKIQKDGTNDH--------IPPPRPKRKAAHPY   126
XP_019053625.1_(NnRVE8-1)      DKFLEALQLFDRDWKKIEDFVGSKTVIQIRSHAQKYFLKVQKNGTVAH--------VPPPRPKRKAAYPY   106
XP_019053626.1_(NnRVE8-2)      DKFLEALQLFDRDWKKIEDFVGSKTVIQIRSHAQKYFLKVQKNGTVAH--------VPPPRPKRKAAYPY   106
LOC_Os06g01670.1_(OsRVE8)      DKFLEALQLFDRDWKKIEAYVGSKTVIQIRSHAQKYFLKVQKNGTGEH--------LPPPRPKRKAAHPY   110
AT3G09600.1_(AtRVE8)           DKFLEALQLFDRDWKKIEDFVGSKTVIQIRSHAQKYFLKVQKNGTLAH--------VPPPRPKRKAAHPY   113

Consensus                      PQKASKXXXXXXX---PXQX---------------------------XXXXXXSXXXXXXXXXXXXXXXP
Mapoly0042s0058.1_(MpRVE1)     PQKA------------PKAV---------------------------------SAVQQPGVLARTLNPQP   132
SELMODRAFT_78045_(SmRVE)       PQKAAKPGKLPI----PNCE--------FHSVFLCSTAPPQRGTPHPPPPDFAYMVPQCNMFVPGVTASP   154
PO057195_(PoRVE1)              PRKFGNTSSKDL----PNLQKPERSISSIQSVSEYETRSPTSVLTTIGSDTMGSSASHIAKHCRSLDSYT   195
Zosma38g00570.1_(ZmRVE8)       PQKASKNDTKSSQFSNPLQAPSYSVVSEQGYSFKRAVVDSSSIQGTPNAITIAPCWSQNTTTPISIDNAP   196
XP_019053625.1_(NnRVE8-1)      PQKASKN---ALL---PLQA---------------------------------SMAYPSTLNCVAPGYSP   137
XP_019053626.1_(NnRVE8-2)      PQKASKN---ALL---PLQA---------------------------------SMAYPSTLNCVAPGYSP   137
LOC_Os06g01670.1_(OsRVE8)      PHKASKRAPQVVL---PQQA---------------------------SHLMEQGCLIPMDISPVARNFNA   150
AT3G09600.1_(AtRVE8)           PQKASKN---AQM---PLQV---------------------------------STSFTTTRNGDMPGYAS   144

Consensus                      ---------------XXXW---------------------------------------------------
Mapoly0042s0058.1_(MpRVE1)     ------------------T---------------------------------------------------   133
SELMODRAFT_78045_(SmRVE)       ---------------VSSWVHHG-----------------------------------------------   162
PO057195_(PoRVE1)              --------TSSSPTGLSLT---------------------------------------------------   206
Zosma38g00570.1_(ZmRVE8)       SWSQNTTIPTISIDDLPSWSQNTTIPTISIDDLPCWSHNTTTPISIDDLPCWSQNTTKPISIDDFPGWSQ   266
XP_019053625.1_(NnRVE8-1)      ------------------W---------------------------------------------------   138
XP_019053626.1_(NnRVE8-2)      ------------------W---------------------------------------------------   138
LOC_Os06g01670.1_(OsRVE8)      ------------NDVFSSW---------------------------------------------------   157
AT3G09600.1_(AtRVE8)           ------------------W---------------------------------------------------   145

Consensus                      -------------------------------------------------------------DXXXX----
Mapoly0042s0058.1_(MpRVE1)     -------------------------------------------------------------QSEPC----   138
SELMODRAFT_78045_(SmRVE)       -------------------------------------------------------------GPQGT----   167
PO057195_(PoRVE1)              -------------------------------------------------------------EQENM----   211
Zosma38g00570.1_(ZmRVE8)       NTTAPISIDDLPISIDDFPGWSQNTTAPISIDDLPISIDDLPISIDDFPCWSQNTTTPISIDDLPCWSQN   336
XP_019053625.1_(NnRVE8-1)      -------------------------------------------------------------DDKSV----   143
XP_019053626.1_(NnRVE8-2)      -------------------------------------------------------------DDKSV----   143
LOC_Os06g01670.1_(OsRVE8)      -------------------------------------------------------------DSALA----   162
AT3G09600.1_(AtRVE8)           -------------------------------------------------------------DDASM----   150

Consensus                      ----------------------------------------------------------------------
Mapoly0042s0058.1_(MpRVE1)     ----------------------------------------------------------------------   138
SELMODRAFT_78045_(SmRVE)       ----------------------------------------------------------------------   167
PO057195_(PoRVE1)              ----------------------------------------------------------------------   211
Zosma38g00570.1_(ZmRVE8)       TTTPISTNNISSGKDFTNDWRHVGSSKNAPNKNFCSSSGKDNQGHVGSSKNAPNKNFCSSSGKDDWGHVG   406
XP_019053625.1_(NnRVE8-1)      ----------------------------------------------------------------------   143
XP_019053626.1_(NnRVE8-2)      ----------------------------------------------------------------------   143
LOC_Os06g01670.1_(OsRVE8)      ----------------------------------------------------------------------   162
AT3G09600.1_(AtRVE8)           ----------------------------------------------------------------------   150
```

```
Consensus                        ---------------------------------XXXXXXX--------RXXXXXXXXXXX----------
Mapoly0042s0058.1_(MpRVE1)       ---------------------------------LP-------------RRGNFQSCSI------------ 150
SELMODRAFT_78045_(SmRVE)         ---------------------------------IPFY-----------KGGSFNQNNSST---------- 183
PO057195_(PoRVE1)                ---------------------------------CQSHPSSIEEGNGSPRSGSTSPTMTTEGKSDMELEVA 248
Zosma38g00570.1_(ZmRVE8)         SSKNAPNKNFYSSSGKDDWGHVGSSKNAPNKNFCSSSGKDDWGHVGSSKNAPNKNFYSSSGKDDWGHVGS 476
XP_019053625.1_(NnRVE8-1)        ---------------------------------LTSTPSN--------RTAPSQDDYTNL---------- 162
XP_019053626.1_(NnRVE8-2)        ---------------------------------LTSTPSN--------RTAPSQDDYTNL---------- 162
LOC_Os06g01670.1_(OsRVE8)        ---------------------------------QSFSPRH--------THGAANNCSSSV---------- 181
AT3G09600.1_(AtRVE8)             ---------------------------------LL----N--------RVISPQHELATL---------- 165

Consensus                        --------------XXXXXXXXXXXXXXXXXXXXXXX-------------------------XSXSXXXX
Mapoly0042s0058.1_(MpRVE1)       --------------------------------------------------------------GSNSGSWQ 158
SELMODRAFT_78045_(SmRVE)         -----------------ANTWAQDPPAQ----------------------------------ANPQRGRK 202
PO057195_(PoRVE1)                LVDAPVTSLKLFGRTVLITDTSKPNSQVVMKETQCLESLPSTGKEETPEEMEKDMQSSTLIQSENVTQQD 318
Zosma38g00570.1_(ZmRVE8)         SKNAPNKNFCSSSGKGLTDDWGYVGSSKNAPNKNCCSSSGKDDWGHVGSSKNAPNKNGCSSSGSPSETWP 546
XP_019053625.1_(NnRVE8-1)        --------------HAIEADVGSKGVARTGNSNFSGI-------------------------GSSSRALP 193
XP_019053626.1_(NnRVE8-2)        ---------------------------------------------------------------------- 162
LOC_Os06g01670.1_(OsRVE8)        --------------------------------------------------------------ESQSGTCP 189
AT3G09600.1_(AtRVE8)             --------------RGAEADIGSKGLLNVSSPSTSGM-------------------------GSSSRTVS 196

Consensus                        XXXXXXXXXXXXXLXXXPDFAEVYXFIGSVFDPX--TX-GHLXKLKEMXPIXXETVLLLMRNLXXNLSXP
Mapoly0042s0058.1_(MpRVE1)       YHSMPPVVPITTDLRATPDFAEVYKFIGNVFDPGMNSN-HNLKKLKEMAPIDRETVLLLMRNLSINLSSP 227
SELMODRAFT_78045_(SmRVE)         TGEKVDRANGIVTLSAAPAFSEVYKFIGSIFDPG--TA-GHLKKLREMAPIDRETVLLLMRNLAINLSSP 269
PO057195_(PoRVE1)                SSVVSGQGAWNPWSCGIPQLFYCLPLQRAQLNAAQNTTAAPPPWWPFCSGLPFPLMHLPNSGPNKHPSEP 388
Zosma38g00570.1_(ZmRVE8)         ISETSEQIRNIPANRAIPDFVQVHQFIGSVLDPD--TEDNHMETLKQMESIDVETVLLLMKNLTVNLENP 614
XP_019053625.1_(NnRVE8-1)        SCDI--QGKRGSLLHGIPDFAEVYSFIGSVFDPD--TK-GHLQKLKEMDPINFETVLLLMRNLTVNLSSP 258
XP_019053626.1_(NnRVE8-2)        -----------HAIEGIPDFAEVYSFIGSVFDPD--TK-GHLQKLKEMDPINFETVLLLMRNLTVNLSSP 218
LOC_Os06g01670.1_(OsRVE8)        TSEAIEQEIMLPTLRAMPDFAQVYNFLGSIFDPE--TS-GHLQRLREMDPIDVETVLLLMKNLSINLTNP 256
AT3G09600.1_(AtRVE8)             GSEIVRKAKQPPVLHGVPDFAEVYNFIGSVFDPE--TR-GHVEKLKEMDPINFETVLLLMRNLTVNLSNP 263

Consensus                        DFE-------------------------------------------------------------------
Mapoly0042s0058.1_(MpRVE1)       EFEERKLFMPAFEANPDAARPDSSDQHLAMLSATQPPSDGGDDDAAFSGQPSSNHQSDSGDSDSQVDPSH 297
SELMODRAFT_78045_(SmRVE)         DFDQRVKF-------------------------------------------------------------- 277
PO057195_(PoRVE1)                DME-----IPDSDNKHPSAEKEPAEEQQPKLDESVPPTNPKPTSE------------------------- 428
Zosma38g00570.1_(ZmRVE8)         DFE------------------------------------------------------------------- 617
XP_019053625.1_(NnRVE8-1)        DFE------------------------------------------------------------------- 261
XP_019053626.1_(NnRVE8-2)        DFE------------------------------------------------------------------- 221
LOC_Os06g01670.1_(OsRVE8)        NFE------------------------------------------------------------------- 259
AT3G09600.1_(AtRVE8)             DLE------------------------------------------------------------------- 266

Consensus                        XXXKXLXSYXXXXXX-------------------------------------------------------
Mapoly0042s0058.1_(MpRVE1)       GQTRVLPSYSDGPMDVAEHRRLTHEGGSRSVDSVLPIAPYPGLTSSRPMTDNSSAGAHRSMGLHSHPHMA 367
SELMODRAFT_78045_(SmRVE)         ADKRFFRSFQRFCFSGLSRDRSCSFQSTKRRKTEW----------------------------------- 312
PO057195_(PoRVE1)                KQVKGFVPYKRCSAE------------------------------------------------------- 443
Zosma38g00570.1_(ZmRVE8)         EHRKLLSYYS-DQMQ------------------------------------------------------- 631
XP_019053625.1_(NnRVE8-1)        PIKKILSSYD-VNMK------------------------------------------------------- 275
XP_019053626.1_(NnRVE8-2)        PIKKILSSYD-VNMK------------------------------------------------------- 235
LOC_Os06g01670.1_(OsRVE8)        AHRKVLASHGYGMDQ------------------------------------------------------- 274
AT3G09600.1_(AtRVE8)             STRKVLLSYDNVTTE------------------------------------------------------- 281

Consensus                        ------------XXXXXXXXXXXXXXXXXXXX----
Mapoly0042s0058.1_(MpRVE1)       FSGRADVGFGMPLPTLTPGVVGEGNIDNWWS----- 398
SELMODRAFT_78045_(SmRVE)         ------------ILTTSNKVVMKKQQETWRKVRKKH 336
PO057195_(PoRVE1)                ------------RESMQTEISEDRDRQRIRLCL--- 464
Zosma38g00570.1_(ZmRVE8)         ------------------------------------ 631
XP_019053625.1_(NnRVE8-1)        ------------MVGRASGGAATNQDNDLHPC---- 295
XP_019053626.1_(NnRVE8-2)        ------------MVGRASGGAATNQDNDLHPC---- 255
LOC_Os06g01670.1_(OsRVE8)        ------------VKHENLGDLGSTHTLHLPFM---- 294
AT3G09600.1_(AtRVE8)             ------------LPSVVSLVKNSTSDKSA------- 298
```

Consensus Threshold: >50%

Compare to: the consensus

Amino acids that match the reference are marked with yellow highlighting.

Created: 12 Apr 2023

Last Modified: 12 Apr 2023
