# Supplementary material for: Dim artificial light at night alters gene expression rhythms and growth in a key seagrass species (Posidonia oceanica)
Source: Sci Rep. 2023 Jun 30;13:10620. doi: 10.1038/s41598-023-37261-3 (PMC10313690; doi:10.1038/s41598-023-37261-3)
Supplement: Supplementary file 8 — Supplementary Information 8. [file 41598_2023_37261_MOESM8_ESM.pdf]

```
Consensus                      MEWDXXXXXXXX-----XXXEE---------------GXXXXXX-GXXXXXXXXXLX-XXPCGXVVTDAL
Mapoly0004s0235.1_(MpFKF)      MDWDTDTDDSDMSLGSVEESEVQEMDRIRICSELRADDESSHKRVDTSHKRLEVLLH-SSPCGLIVTDAL  69
SELMODRAFT_174189_(SmZTL)      --------------------------------------------------------M-LGPCSVVVTDAL  13
PO066488_(PoZTL)               MEWDGDFGEEEE------VEEE---------------GFLLKDA-ADFTFSVGSMLQ-SSSCGFVVSDAL  47
Zosma25g00320.1_(ZmZTL)        MEWDNVLEEEEE-----EEEEE---------------GFLLTDGVGPFGLSIGNMLQ-SGSCGFVVCDST  49
XP_010265437.1_(NnZTL1)        MEWDSDSDGSGD--------DE---------------GFLLNDG-GPLSFTIGSLLQ-TSPCGFVVTDAL  45
XP_010265441.1_(NnZTL2)        ----------------------------------------------------------------------
AT5G57360.2_(AtZTL)            MEWDSGSDLSADDASSLADDEE---------------GGLFPGG-GPIPYPVGNLLH-TAPCGFVVTDAV  53
LOC_Os02g05700_(OsZTL-1)       MEWDSDSEGSGD-----EEEEEEEEEEEGVEVGGGGDGGVGVGVGGGFALAIEGV---LGACGLVVSDAL  62
LOC_Os06g47890_(OsZTL-2)       MEWDSESDGAGSIGAGEEEEEEEEEEEGGFGGG----GGGGGGGGGMFSFAIEGMLRASGPCGLVVTDAL  66

Consensus                      EPDXPIIYVNXXFEXXTGYRAEEVLGRNCRFLQCRGPFAQRRHPLVDSXVVSEIRRCLXXGXEFQGELLN
Mapoly0004s0235.1_(MpFKF)      EPDQPIIYVNTVFEFITGYKAEEILGKNCRFLQCRGPFAQRRHPLVDPTTVATIKRCLTEGVEFRGELLN  139
SELMODRAFT_174189_(SmZTL)      DVDFPIIYVNNIFEFITGYKAEEVLGRNCRFLQFRGPFAQRRHPLVDSATVTEIRRCMREGIEFWGELLN  83
PO066488_(PoZTL)               EPDQPIIYVNATFEKVTGYRAEEILGGNCHFLQCRGPFAQRRHPLVDSVVVSEIRRCLDKGIEFQGDLLN  117
Zosma25g00320.1_(ZmZTL)        EPDSPIIYVNTTFEKITGYRAEEVLGRNCGFLQCRGPYAQRRHPLVDSAVVSEIRRSLDQGIEFEGQLLN  119
XP_010265437.1_(NnZTL1)        EPDHPIIYVNTGFELATGYRAEEVLGRNCRFLQYRGPFAKRRHPLVDSTVVSEIRRCLEDGVEFQGELLN  115
XP_010265441.1_(NnZTL2)        --------------------MGAHHSSSSRFLQYRGPFAKRRHPLVDSTVVSEIRRCLEDGVEFQGELLN  50
AT5G57360.2_(AtZTL)            EPDQPIIYVNTVFEMVTGYRAEEVLGGNCRFLQCRGPFAKRRHPLVDSMVVSEIRKCIDEGIEFQGELLN  123
LOC_Os02g05700_(OsZTL-1)       EPDFPIIYVNRGFEDATGYRAEEVLGRNCRFLQCRGPFAKRRHPLVDTTVVTDIRRCLEEGTVFQGDLLN  132
LOC_Os06g47890_(OsZTL-2)       EPDCPIIYVNCGFEEATGYRAEEVLGRNCRFLQCRGPFAQRRHPLVDAMVVSEIRKCIDNGTEFRGDLLN  136

Consensus                      FRKDGSPLMNXLXLTPIYGDDDTITHVIGIQFFTEANXDLGPLPGSSXKE----XXXXXXXLXXDXX-YR
Mapoly0004s0235.1_(MpFKF)      FRKDGTPLMNKLCMTPIHAEDGTITHVIGIQSFTEAKLDLGPIP---WKD--SLLWSKDQPLKEANA-YK  203
SELMODRAFT_174189_(SmZTL)      FRKDGTPLMNKLCLKPIRGEDGRITHIIGIQSFSEVKLDLGPLPPPLWRNQSSQHWLVRRHLLDDGYSYA  153
PO066488_(PoZTL)               FRKDGSPLLNRLRLTPIYGDDDVITHVIGIQFFTETNVDLGPLPGSSVKE---SFKPSESSLADRPY-YR  183
Zosma25g00320.1_(ZmZTL)        FRKDGSPLMNNLKLAPIYGDDDIISHVIGIQFFTEANVDLGPLVAVSSCF---NKKPCIKSNRDESF-YR  185
XP_010265437.1_(NnZTL1)        FRKDGSPLMNRLRLTPIFGDDDTITHVIGIQFFTEANLDLGPLPGSSAKE----STKASERLHSDLL-CR  180
XP_010265441.1_(NnZTL2)        FRKDGSPLMNRLRLTPIFGDDDTITHVIGIQFFTEANLDLGPLPGSSAKE----STKASERLHSDLL-CR  115
AT5G57360.2_(AtZTL)            FRKDGSPLMNRLRLTPIYGDDDTITHIIGIQFFIETDIDLGPVLGSSTKE-----------KSIDGI-YS  181
LOC_Os02g05700_(OsZTL-1)       FRKDGSPFMAKLQLTPIYGDDETITHYMGMQFFNDSNVDLGPLSVSTTKE-----IVRSTLITPDNT-IR  196
LOC_Os06g47890_(OsZTL-2)       FRKDGSPLMNKLHLTPIYGDDETITHYMGIQFFTNANVDLGPLPGSLTKE-----PVRSTRFTPDNF-FR  200

Consensus                      PXPXGXG--XXXREXCGLXQLSDEVLXQKILSRLXPRDIASVGSVCRRLYXLTKNXDLWRMVCQNAWGSE
Mapoly0004s0235.1_(MpFKF)      SSPVGPG--QSSKDACGLLQLSDEVLTHKIIAYVAPRDVAALGLVCRRLHEITKNDNLWRSVCQNSWGFE  271
SELMODRAFT_174189_(SmZTL)      SSPVGPG--QSSKDRCGILRLSDEVLVQKILAQLTPRDVSSVALVCRRFNEMTKNTDLWRLVCRNAWGLE  221
PO066488_(PoZTL)               PISVGRG--DMPREFCCFWQLSDELLSQKILSRLSPRDVASVGSVCRHLYALTKDDDLWRMVCQNAWGTE  251
Zosma25g00320.1_(ZmZTL)        PLSVSSG-VGGNREFCFLWQLSDEVLSQKIFSKLPPRDIASIGSVCKQLYELTKSDDLWRSVCRNAWGSE  254
XP_010265437.1_(NnZTL1)        PVPIGHG--NVCREICGMLQLSDEVLALKILSRLTPRDIASVGSVCKRLYQLTKNEDLWRMVCQNAWGSE  248
XP_010265441.1_(NnZTL2)        PVPIGHG--NVCREICGMLQLSDEVLALKILSRLTPRDIASVGSVCKRLYQLTKNEDLWRMVCQNAWGSE  183
AT5G57360.2_(AtZTL)            ALAAGER--NVSRGMCGLFQLSDEVVSMKILSRLTPRDVASVSSVCRRLYVLTKNEDLWRRVCQNAWGSE  249
LOC_Os02g05700_(OsZTL-1)       PSPMGKG---FCSEHSDLFLLSDEVLCQKILSRLSPRDIASVNSVCKRLYHLTRNDDLWRMVCQNAWGSE  263
LOC_Os06g47890_(OsZTL-2)       PISTGPGQSNFCREYSSLFQLTDEVLCQSILSRLSPRDIASVSSVCRRLYLLTRNEDLWRMVCQNAWGSE  270

Consensus                      TTXVLETVPGAKRLGWGRLARELTTLEAAAWRKLTVGGAVEPSRCNFSACAVGNRVVLFGGEGVNMQPMN
Mapoly0004s0235.1_(MpFKF)      ATKALESVPGADNLEWGRLARELTTLEAAAWRKLTVGGAVEPSRCNFSACAVGNKLVLFGGEGVNMQPMN  341
SELMODRAFT_174189_(SmZTL)      TTAVLERVHNPRSIDWGMLARELTTLEAAAWRKLKVGGAVEPSRCNFSACAVGNKVVLFGGEGVNMQPMN  291
PO066488_(PoZTL)               TTCELETVPEAKRLGWGRLARELTTLEAVAWRKLTVGGSVEPSRCNFSACAVGNRVVLFGGEGVNMQPMN  321
Zosma25g00320.1_(ZmZTL)        TTTALETFAREKWLGWGRLARELTTLEAATWRKLTVGGSVEPSRCNFSVCAVGSRVVLFGGEGVNMQPMN  324
XP_010265437.1_(NnZTL1)        TTRVLETVPGAKRLGWGRLARELTTLEAAAWRKLTVGGAVEPSRCNFSACAVGNRVVLFGGEGVNMQPMN  318
XP_010265441.1_(NnZTL2)        TTRVLETVPGAKRLGWGRLARELTTLEAAAWRKLTVGGAVEPSRCNFSACAVGNRVVLFGGEGVNMQPMN  253
AT5G57360.2_(AtZTL)            TTRVLETVPGAKRLGWGRLARELTTLEAAAWRKLSVGGSVEPSRCNFSACAVGNRVVLFGGEGVNMQPMN  319
LOC_Os02g05700_(OsZTL-1)       ATQVLETVAGTRSLAWGRLARELTTLEAVTWRKLTVGGAVEPSRCNFSACAAGNRVVLFGGEGVNMQPMN  333
LOC_Os06g47890_(OsZTL-2)       TTRALETVPAAKRLGWGRLARELTTLEAVAWRKLTVGGAVEPSRCNFSACAVGNRVVLFGGEGVNMQPMN  340
```

```
Consensus                        DTFVLDLNASXPEWRHVXVSSPPPGRWGHTLSCLNGSXLVVFGGCGRQGLLNDVFXLDLDAKXPTWREI-
Mapoly0004s0235.1_(MpFKF)        DTFVLDLSVKHPAWQHVNVKSPPPGRWGHTLSCLNKSWLVVFGGCGRDGLLNDVFIVDLDAKQPIWREIG  411
SELMODRAFT_174189_(SmZTL)        DTFVLDLSAACPEWRHVDVGSAPPGRWGHTLSCLNGSWLVVFGGCGRQGLLNDVFVLDLDAKQPSWREV-  360
PO066488_(PoZTL)                 DTFVLDLNASRPEWTHVKVDSPPPGRWGHTLTCLNGSLLVVFGGCGRQGLLNDVFILDLDAKHPTWREI-  390
Zosma25g00320.1_(ZmZTL)          DTFVLDLSSTEPEWRHVNVSSPPPGRWGHTVSCLNDSWLVVFGGCGRQGLLNDVFILDLDAKNPTWREI-  393
XP_010265437.1_(NnZTL1)          DTFVLDLNASNPEWRHVKVSSPPPGRWGHTLSCVNGSLLVVFGGCGRQGLLNDVFVLDLDAQHPTWREI-  387
XP_010265441.1_(NnZTL2)          DTFVLDLNASNPEWRHVKVSSPPPGRWGHTLSCVNGSLLVVFGGCGRQGLLNDVFVLDLDAQHPTWREI-  322
AT5G57360.2_(AtZTL)              DTFVLDLNSDYPEWQHVKVSSPPPGRWGHTLTCVNGSNLVVFGGCGQQGLLNDVFVLNLDAKPPTWREI-  388
LOC_Os02g05700_(OsZTL-1)         DTFVLDLNASKPEWRHINVRSAPPGRWGHTLSCLNGSRLVLFGGCGRQGLLNDVFMLDLDAQQPTWREI-  402
LOC_Os06g47890_(OsZTL-2)         DTFVLDLNASNPEWRHVNVSSAPPGRWGHTLSCLNGSLLVVFGGCGRQGLLNDVFTLDLDAKQPTWREI-  409
```

```
Consensus                        SGLAPPLPRSWHSSCTLDGTKLVVSGGCADSGVLLSDTFLLDXTMEKPVWREIPVSWTPPSRLGHTLSVY
Mapoly0004s0235.1_(MpFKF)        AGAVAPIPRSWHSSCTLDGTKLVVSGGCADSGVLLSDTFLLDLTMEKPMWREIHVSWSPPSRLGHTLCVY  481
SELMODRAFT_174189_(SmZTL)        AGVGPPVPRSWHSSCTLDGTQLVVYGGCADSGVLLSDTYMLDISKEKPMWREIPVAWTPPSRLGHSLSAY  430
PO066488_(PoZTL)                 SGLAPPLPRSWHSSCTLDGTKLVVSGGCADSGVLLSDTFMLDVTMEKPIWREIPVTWTPPSRLGHTLSVY  460
Zosma25g00320.1_(ZmZTL)          SGVAPPLPRSWHSSCTLDGTMLVVSGGCTDSGVLLSDTFTLDVTVDKPVWREIPVAWKPPSRLGHTLSVY  463
XP_010265437.1_(NnZTL1)          SGLAPPLPRSWHSSCTLDGSKLVVSGGCADSGVLLSDTFLLDLSMEKPVWREIPVSWTPPSRLGHTLSVY  457
XP_010265441.1_(NnZTL2)          SGLAPPLPRSWHSSCTLDGSKLVVSGGCADSGVLLSDTFLLDLSMEKPVWREIPVSWTPPSRLGHTLSVY  392
AT5G57360.2_(AtZTL)              SGLAPPLPRSWHSSCTLDGTKLIVSGGCADSGVLLSDTFLLDLSIEKPVWREIPAAWTPPSRLGHTLSVY  458
LOC_Os02g05700_(OsZTL-1)         PGLAPPVPRSWHSSCTLDGTKLVVSGGCADSGVLLSDTYLLDVTMERPVWREIPASWTPPCRLGHSLSVY  472
LOC_Os06g47890_(OsZTL-2)         PGVAPPVPRSWHSSCTLDGTKLVVSGGCADSGVLLSDTYLLDVTMDKPVWREVPASWTPPSRLGHSMSVY  479
```

```
Consensus                        GGRKILMFGGLAKSGPLRLRSSDVFTMDLSEEEPCWRCVTGSGMPGAGNPAGXXPPPRLDHVAVSLPGGR
Mapoly0004s0235.1_(MpFKF)        QGWKVLMFGGLAKSGPLRLRSSDVFTIDLSEEQPKWKYVTGSTLPGGAAPAGTPPPPRLDHVAVSLPGGR  551
SELMODRAFT_174189_(SmZTL)        GGRKILLFGGLAKSGPLRFRSSDAFTIDLGEEEPTWKYVTGSTLPGGANIGGTTPPPRLDHVAVTLPGGR  500
PO066488_(PoZTL)                 DGRKMLMFGGLAKSGPLRLRSNDVYTMDLSEEEPYWRCITGSGMPGAGNPAGMSPPPRLDHVAVSLPDGR  530
Zosma25g00320.1_(ZmZTL)          DGRKILMFGGLAKSGPLRLRSSDVYTMDLSEEEPCWRCITGSGMPGAGNPAGIGPPPRLDHVAVSLPGGR  533
XP_010265437.1_(NnZTL1)          GGRKILMFGGLAKSGSLRFRSSDVFTMDLSEEEPCWRCVTGSAMPGTGNPAGIAPPPRLDHVAVNLPGGR  527
XP_010265441.1_(NnZTL2)          GGRKILMFGGLAKSGSLRFRSSDVFTMDLSEEEPCWRCVTGSAMPGTGNPAGIAPPPRLDHVAVNLPGGR  462
AT5G57360.2_(AtZTL)              GGRKILMFGGLAKSGPLKFRSSDVFTMDLSEEEPCWRCVTGSGMPGAGNPGGVAPPPRLDHVAVNLPGGR  528
LOC_Os02g05700_(OsZTL-1)         DGRKILMFGGLAKSGPLRLRSNDVFTLDLSENKPCWRCITGSGMPGASNPAGVGPPPRLDHVAVSLPGGR  542
LOC_Os06g47890_(OsZTL-2)         GGRKILMFGGLAKSGPLRLRSSDVFTMDLSEEEPCWRCLTGSGMPGAGNPAGAGPPPRLDHVAVSLPGGR  549
```

```
Consensus                        ILIFGGSVAGLHSASQLYLLDPTEEKPTWRILNVPGRPPRFAWGHSTCVVGGTRAIVLGGQTGEEWMLX-
Mapoly0004s0235.1_(MpFKF)        ILIFGGSIAGLHSPAQLFVLDPKEEQSTWRVLNVPGQPPKFAWGHSTCVVGGTRAVVLGGHTGEEWILN-  620
SELMODRAFT_174189_(SmZTL)        ILIFGGSIAGLHSASQIYLLDPSEEKPTWRMLNVPGQKPKFAWGHSTCFVGGTRAVVLGGHTGEDWILN-  569
PO066488_(PoZTL)                 ILIFGGSIAGLHSASQLYLLDPTEEKPTWRIMNIPGRPPRFAWGHSTCVVGGTRALVLGCQTGEEWMLS-  599
Zosma25g00320.1_(ZmZTL)          ILIFGGSVAGLHSASQLFLLDPTEENPTWRIMNVPGRPPRFAWGHSTCVVGGSRAIVLGGQTGEEWMLS-  602
XP_010265437.1_(NnZTL1)          ILIFGGSVAGLHSASQLYLLDPTDEKPTWRILNVPGRPPRFAWGHSTCIVGGTRAIVLGGQTGEEWMLS-  596
XP_010265441.1_(NnZTL2)          ILIFGGSVAGLHSASQLYLLDPTDEKPTWRILNVPGRPPRFAWGHSTCIVGGTRAIVLGGQTGEEWMLS-  531
AT5G57360.2_(AtZTL)              ILIFGGSVAGLHSASQLYLLDPTEDKPTWRILNIPGRPPRFAWGHGTCVVGGTRAIVLGGQTGEEWMLRY  598
LOC_Os02g05700_(OsZTL-1)         ILIFGGSVAGLHSASKLYLLDPTEEKPTWRILNVPGRPPRFAWGHSTCVVGGTKAIVLGGQTGEEWTLT-  611
LOC_Os06g47890_(OsZTL-2)         VLIFGGSVAGLHSASQLYLLDPTEEKPTWRILNVPGRPPRFAWGHSTCVVGGTKAIVLGGQTGEEWMLT-  618
```

```
Consensus                        -ELHELSLASSXX---------------
Mapoly0004s0235.1_(MpFKF)        -ELHELSITHKSLSST------------  635
SELMODRAFT_174189_(SmZTL)        -ELHELSLSSTSQWIRRLQ---------  587
PO066488_(PoZTL)                 -ELYELSLANSVI---------------  611
Zosma25g00320.1_(ZmZTL)          -ELYELSMASF-----------------  612
XP_010265437.1_(NnZTL1)          -ELHELSLASSVM---------------  608
XP_010265441.1_(NnZTL2)          -ELHELSLASSVM---------------  543
AT5G57360.2_(AtZTL)              WSFRGERLSGGTLVLLIFFKSFFFFLPH  626
LOC_Os02g05700_(OsZTL-1)         -ELHELSLVSSLV---------------  623
LOC_Os06g47890_(OsZTL-2)         -EIHELSLASSTV---------------  630
```

Consensus Threshold: >50%

Compare to: the consensus

Amino acids that match the reference are marked with yellow highlighting.

Created: 12 Apr 2023

Last Modified: 12 Apr 2023
