# Supplementary material for: Dim artificial light at night alters gene expression rhythms and growth in a key seagrass species (Posidonia oceanica)
Source: Sci Rep. 2023 Jun 30;13:10620. doi: 10.1038/s41598-023-37261-3 (PMC10313690; doi:10.1038/s41598-023-37261-3)
Supplement: Supplementary file 9 — Supplementary Information 9. [file 41598_2023_37261_MOESM9_ESM.pdf]

Supplementary Material

Title

Dim artificial light at night alters gene expression rhythms and growth in a key seagrass species (*Posidonia oceanica*)

Dalle Carbonare L.[1], Basile A.[1], Rindi L.[2], Bulleri F.[2], Hamedeh H.[1], Iacopino S.[1], Shukla V.[1], Weits D.A.[1], Lombardi L.[2], Sbrana A.[2], Benedetti-Cecchi L.[2], Giuntoli B.[1,2], Licausi F.[2,3] and Maggi E.[2*]

1 Institute of Life Sciences, Scuola Superiore Sant'Anna, Piazza Martiri della Liberta' 56127, Pisa, IT

2 Dipartimento di Biologia, Universita' di Pisa, CoNISMa, Via Luca Ghini 13, 56126 Pisa, IT

3 Department of Plant Sciences, University of Oxford, OX1 3RB, Oxford, UK

*Corresponding author

Dr. Elena Maggi

Dipartimento di Biologia, Universita' di Pisa, Pisa, Italy

E-mail: elena.maggi@unipi.it; phone number: +39 050 2211444

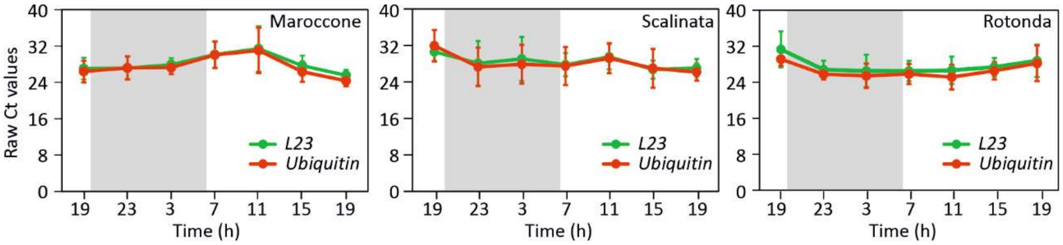

Supplementary Figure S1. Raw Ct values for *60s ribosomal protein L23* (*L23*) (green) and *Ubiquitin* (red) reference genes during the time course sampling in Maroccone, Scalinata and Rotonda sites. Samples were collected every four hours, starting from seven o'clock in the evening for a total period of twenty-four hours. Data are presented as mean ± SD (standard deviation, number of replicates: *n* = 10).

Supplementary Table S1. List of gene names, gene ID and protein sequences used for the phylogenetic analysis.

Supplementary Table S2. List of forward (5'-> 3') and reverse (5'-> 3') primers sequences, correlated with percent efficiency (E) for *Posidonia oceanica* qPCR analysis.

Gene name	Gene code	Forward (5'->3')	Reverse (5'->3')	*E (%)*
PoPRR5-2	*PO007080*	atgttagtgcagttgctggc	ctctcggtctcgctacactt	119
PoELF3	*PO041070*	ccgcaccttctgcttgaaat	gtttcatatgctgcggctga	104
PoLUX	*PO025399*	gggacgatgctgaattggac	ccttgttctcatccccacct	120
PoREV1	*PO057195*	ttgcctaacttgcagaagcc	aaacagaggttggggatcgt	106
PoCCA1	*PO030913*	gcgactccaccaagtttcag	tgcactgtgttagctcctca	101
PoPRR5-1	*PO053273*	gtgatgcaagtccaggtgtg	acattcgccctttctctcga	107
PoZTL	*PO066488*	cagaccagagtggacgcacg	taaaccctgcctgccacagc	127
PoGI	*PO021005*	gctgggttgccagctcttga	agcagcgtcaagggcatctg	100
PoCO	*PO020219*	gttcatcaccgtcctccgcc	gtgtggcttggatctggcga	136
PoADH	*PO008911*	gagagtgttggcgaaggcgt	aggtcgcacatgttgctctcc	109
PoPSBS		Ruocco *et al*., 2012		110
PoCAB6-A		Mazzuca *et al*., 2013		101
PoCAB-151		Mazzuca *et al*., 2013		111
PoSSU5B		Ruocco *et al*., 2012		114
PoLHCA4		Ruocco *et al*., 2012		103
PoLHCB4.2		Mazzuca *et al*., 2013		112
PoZEP		Ruocco *et al*., 2012		102
PoSEND33		Mazzuca *et al*., 2013		110
PoPSAG		Ruocco *et al*., 2012		104

Supplementary Table S3. mRNA relative expression levels for all the genes analysed in this study, indicated as expression fold change (x^-FC), divided by site and sampling time. Night sky brightness for each site is indicated as mag/arcsec^2.

Supplementary Table S4. Maximum quantum efficiency of PSII expressed as Fv/Fm of plants from Maroccone, Scalinata and Rotonda. Fm indicates the maximum fluorescence after the saturation of the light pulse, F0 is the minimum fluorescence after dark adaptation.

Date	Time	Site	Colony	Replicate	Fo	Fm	Fv/Fm	Temperature
13/07/2018	08:20:43	Maroccone	1	1	567	2458	0.769	25
13/07/2018	08:29:06	Maroccone	1	2	233	1432	0.837	24
13/07/2018	08:29:23	Maroccone	1	3	457	2405	0.81	25
13/07/2018	08:41:58	Maroccone	2	1	183	955	0.808	25
13/07/2018	08:42:12	Maroccone	2	2	411	2371	0.827	25
13/07/2018	08:43:50	Maroccone	3	1	156	1341	0.884	25
13/07/2018	08:44:07	Maroccone	3	2	472	2589	0.818	25
13/07/2018	08:44:24	Maroccone	3	3	130	716	0.818	25
13/07/2018	08:56:49	Maroccone	4	1	606	3600	0.832	25
13/07/2018	08:57:08	Maroccone	4	2	495	3169	0.844	25
13/07/2018	08:57:34	Maroccone	4	3	585	3919	0.851	25
13/07/2018	08:57:57	Maroccone	5	1	372	1709	0.782	25
13/07/2018	08:58:18	Maroccone	5	2	132	639	0.793	25
13/07/2018	08:58:33	Maroccone	5	3	552	2997	0.816	25
13/07/2018	09:46:19	Scalinata	1	1	515	2717	0.81	25
13/07/2018	09:46:28	Scalinata	1	2	431	2665	0.838	25
13/07/2018	09:46:37	Scalinata	1	3	600	2602	0.769	25
13/07/2018	09:53:37	Scalinata	2	1	715	3178	0.775	26
13/07/2018	09:53:46	Scalinata	2	2	634	2882	0.78	26
13/07/2018	09:53:55	Scalinata	2	3	617	2717	0.773	26
13/07/2018	09:54:52	Scalinata	3	1	477	2675	0.822	26
13/07/2018	09:55:02	Scalinata	3	2	489	3282	0.851	26
13/07/2018	09:55:15	Scalinata	3	3	527	2171	0.757	26
13/07/2018	10:01:41	Scalinata	4	1	532	2156	0.753	25
13/07/2018	10:01:51	Scalinata	4	2	437	2418	0.819	25
13/07/2018	10:02:00	Scalinata	4	3	501	2242	0.777	25
13/07/2018	10:02:47	Scalinata	5	1	635	3162	0.799	25
13/07/2018	10:02:59	Scalinata	5	2	574	3295	0.826	25
13/07/2018	10:03:13	Scalinata	5	3	641	2918	0.78	25
13/07/2018	10:28:55	Rotonda	1	1	743	3469	0.786	29
13/07/2018	10:31:55	Rotonda	2	1	593	2587	0.771	29
13/07/2018	10:34:55	Rotonda	1	2	221	1331	0.834	29
13/07/2018	10:35:12	Rotonda	1	3	475	2985	0.841	29
13/07/2018	10:43:39	Rotonda	2	2	507	3100	0.836	29
13/07/2018	10:43:48	Rotonda	2	3	410	2356	0.826	29
13/07/2018	10:44:46	Rotonda	3	1	679	3091	0.78	29

13/07/2018	10:44:55	Rotonda	3	2	628	3339	0.812	29
13/07/2018	10:45:07	Rotonda	3	3	375	2362	0.841	29
13/07/2018	10:52:31	Rotonda	4	1	573	2960	0.806	29
13/07/2018	10:52:39	Rotonda	4	2	499	2783	0.821	29
13/07/2018	10:52:49	Rotonda	4	3	443	3096	0.857	29
13/07/2018	10:53:53	Rotonda	5	1	506	3204	0.842	30
13/07/2018	11:01:50	Rotonda	5	2	474	3406	0.861	29
13/07/2018	11:02:14	Rotonda	5	3	498	3275	0.848	29

Supplementary File S1. Protein alignment of CCA1 in *Marchantia polymorpha*, *Selaginella moellendorffii*, *Posidonia oceanica*, *Zostera marina*, *Nelumbo nucifera*, *Oryza sativa* and *Arabidopsis thaliana*. Created with SnapGene software version 6.2.1 (www.snapgene.com).

Supplementary File S2. Protein alignment of C0 in *Marchantia polymorpha*, *Selaginella moellendorffii*, *Posidonia oceanica*, *Zostera marina*, *Nelumbo nucifera*, *Oryza sativa* and *Arabidopsis thaliana*. Created with SnapGene software version 6.2.1 (www.snapgene.com).

Supplementary File S3. Protein alignment of ELF3 in *Marchantia polymorpha*, *Selaginella moellendorffii*, *Posidonia oceanica*, *Zostera marina*, *Nelumbo nucifera*, *Oryza sativa* and *Arabidopsis thaliana*. Created with SnapGene software version 6.2.1 (www.snapgene.com).

Supplementary File S4. Protein alignment of GI in *Marchantia polymorpha*, *Selaginella moellendorffii*, *Posidonia oceanica*, *Zostera marina*, *Nelumbo nucifera*, *Oryza sativa* and *Arabidopsis thaliana*. Created with SnapGene software version 6.2.1 (www.snapgene.com).

Supplementary File S5. Protein alignment of LUX in *Marchantia polymorpha*, *Selaginella moellendorffii*, *Posidonia oceanica*, *Zostera marina*, *Nelumbo nucifera*, *Oryza sativa* and *Arabidopsis thaliana*. Created with SnapGene software version 6.2.1 (www.snapgene.com).

Supplementary File S6. Protein alignment of PRR5 in *Marchantia polymorpha*, *Selaginella moellendorffii*, *Posidonia oceanica*, *Zostera marina*, *Nelumbo nucifera*, *Oryza sativa* and *Arabidopsis thaliana*. Created with SnapGene software version 6.2.1 (www.snapgene.com).

Supplementary File S7. Protein alignment of RVE in *Marchantia polymorpha*, *Selaginella moellendorffii*, *Posidonia oceanica*, *Zostera marina*, *Nelumbo nucifera*, *Oryza sativa* and *Arabidopsis thaliana*. Created with SnapGene software version 6.2.1 (www.snapgene.com).

Supplementary File S8. Protein alignment of ZTL in *Marchantia polymorpha*, *Selaginella moellendorffii*, *Posidonia oceanica*, *Zostera marina*, *Nelumbo nucifera*, *Oryza sativa* and *Arabidopsis thaliana*. Created with SnapGene software version 6.2.1 (www.snapgene.com).
